# Rich Landscape of Colloidal Semiconductor–Metal Hybrid Nanostructures: Synthesis, Synergetic Characteristics, and Emerging Applications

**DOI:** 10.1021/acs.chemrev.2c00770

**Published:** 2023-02-03

**Authors:** Yuval Ben-Shahar, David Stone, Uri Banin

**Affiliations:** † Department of Physical Chemistry, Israel Institute for Biological Research, P.O. Box 19, Ness Ziona74100, Israel; ‡ The Institute of Chemistry and Center for Nanoscience and Nanotechnology, The Hebrew University of Jerusalem, Jerusalem91904, Israel

## Abstract

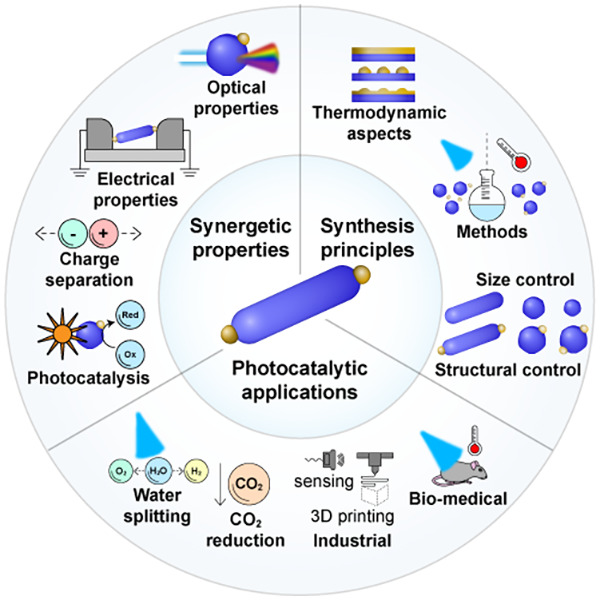

Nanochemistry provides powerful synthetic tools allowing one to combine different materials on a single nanostructure, thus unfolding numerous possibilities to tailor their properties toward diverse functionalities. Herein, we review the progress in the field of semiconductor–metal hybrid nanoparticles (HNPs) focusing on metal–chalcogenides–metal combined systems. The fundamental principles of their synthesis are discussed, leading to a myriad of possible hybrid architectures including Janus zero-dimensional quantum dot-based systems and anisotropic quasi 1D nanorods and quasi-2D platelets. The properties of HNPs are described with particular focus on emergent synergetic characteristics. Of these, the light-induced charge-separation effect across the semiconductor–metal nanojunction is of particular interest as a basis for the utilization of HNPs in photocatalytic applications. The extensive studies on the charge-separation behavior and its dependence on the HNPs structural characteristics, environmental and chemical conditions, and light excitation regime are surveyed. Combining the advanced synthetic control with the charge-separation effect has led to demonstration of various applications of HNPs in different fields. A particular promise lies in their functionality as photocatalysts for a variety of uses, including solar-to-fuel conversion, as a new type of photoinitiator for photopolymerization and 3D printing, and in novel chemical and biomedical uses.

## 1 Introduction

**Scheme 1:**
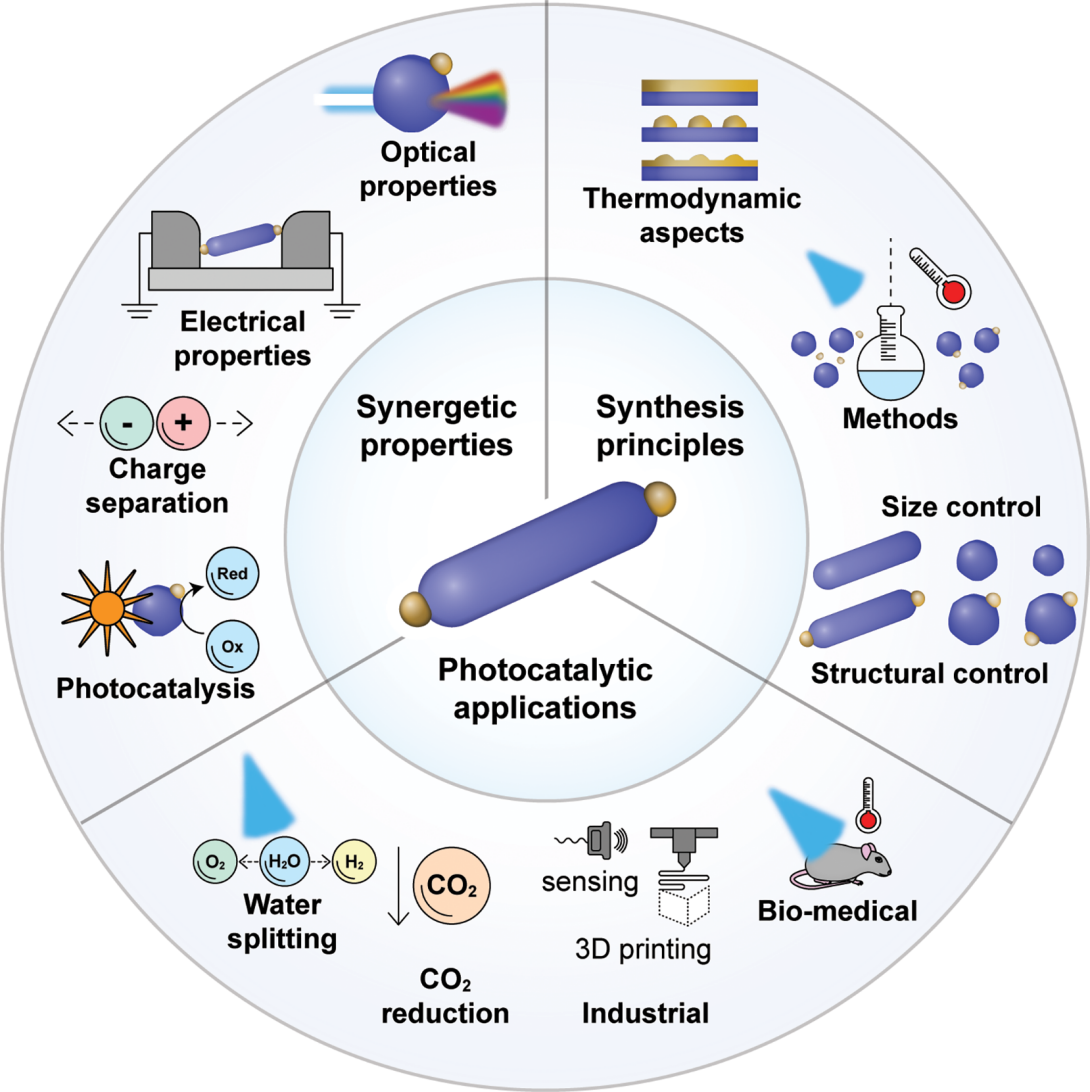
Synergetic Characteristics of Colloidal Semiconductor–Metal HNPs from Synthesis to Emerging Application

Semiconductor–metal hybrid nanoparticles (HNPs) combine two disparate materials on a single nanosystem.^1^ The motivation for such unconventional combination of materials arises, first, from the great quest of expanding the horizons of nanochemistry to form heterogeneous systems. This achievement has risen and continues to strive on the basis of the ongoing significant synthetic advances in forming, on the one hand, highly controlled colloidal semiconductor nanostructures as well as their metal counterparts, on the other hand. Second, such hybrid semiconductor–metal nanoparticles manifest a combination of the original properties of their constituents on a single nanosystem. Moreover, they reveal unique synergistic effects emerging from the nanoscale semiconductor–metal combination, thus providing an outcome greater than the simple sum of its parts.^2,3^ The ongoing developments in synthesis of HNPs along with the in-depth understanding of their combined and synergetic physical and chemical properties enable their functionality in numerous promising applications. This includes utilization of HNPs in optical, electronic, biomedical, and environmental fields.^4−7^ A notable direction is the application of HNPs as efficient photocatalysts for timely challenges including direct solar-to-fuel conversion, waste degradation, and water purification, as novel photopolymerization and photocuring agents, for antimicrobial functions, and in photodynamic therapies. These different aspects of HNPs are illustrated in Scheme 1.

Joining semiconductor and metal segments into a single nanoparticle introduces several fundamental synthetic challenges. The synthesis design needs to overcome the competing homogeneous nucleation of either semiconductor or metal phase that would form separate nanosystems instead of a unified hybrid nanoparticle. Additionally, lattice mismatching can hinder the formation of stable semiconductor–metal junctions. Another factor is the competition with cation exchange reactions and metal diffusion that would generate a nanostructure with altered composition rather than the desired HNP. Last but not least, a basic challenge for the colloidal nanosystems is the tailoring of the surface coating needed both for colloidal chemical stability as well as for chemical and physical passivation, and in HNPs, both the semiconductor and metal nanosurfaces must be considered in tandem. This review presents, in section 2, the vastly rich synthetic approaches and strategies successfully addressing the above challenges and yielding numerous semiconductor–metal HNPs with different compositions and dimensionalities. The focus is primarily on semiconductor nanostructures from the well-developed family of metal–chalcogenides with diverse metals. The extensive investigation and in-depth understanding of metal–chalcogenides fundamental physical properties allowing one to decipher their energy band structure and electronic properties such as excited charge carriers dynamics set the ground for their implementation in hybrid nanosystems. Specifically, studies of metal–chalcogenide-based HNPs, such as CdS and CdSe/CdS in the forms of dots, rods, and platelets, in the context of their photocatalytic application are highly interesting due to their tunable band-gap energy covering the blue to visible to near-infrared region and the lower recombination rates, giving them the ability to efficiently utilize the solar spectrum. Other related systems including metal–oxide nanostructures with metals, metal–metal combinations, etc., are covered in prior other reviews and are not addressed herein.^8−14^

The successful synthesis of semiconductor–metal HNPs has enabled the ongoing study of their emergent properties. The semiconductor nanostructure properties that can be tuned by control of the size and shape via quantum confinement as well as by altering the composition can now be augmented and enhanced by the features of the combined metal nanosegment. This opens up novel possibilities, for example, contact metal points directly grown on the semiconductor nanostructure for facile electrical wiring and as chemical anchor points. Manipulating the semiconductor optical properties allows one to achieve absorption enhancement and emission enhancement or quenching via near-field effects and interactions induced by coupling to the metal. One of the most prominent properties arises from the combination of excitonic semiconductor features with the plasmonic metal response and their couplings that lead to light-induced charge separation from either side of the semiconductor–metal HNPs. This charge separation is affected by several key parameters. Among these parameters are structural effects such as the metal component size and type as was demonstrated in various reports on similar CdS-based HNPs with different metal sites including Au and Pt, all showing size-dependent charge-transfer dynamics. The light-induced charge-separation characteristic constitutes the first step that provides excited charge carriers on both the semiconductor and the metal segments, serving as the basis for their photocatalytic activity toward different redox reaction pathways. Section 3 surveys the emergent combined and synergetic properties of the HNPs including the fundamental basis of such behavior while emphasizing the recent findings.

The advanced synthetic control of the semiconductor–metal HNPs provides the ability to tailor their characteristics according to the requirements of specific photocatalytic applications. A shining example is the ability to match the semiconductor nanostructure gap with a particular illumination source while tailoring the metal segment for a specific chemical reaction. This opens the path for using HNPs as a photocatalyst for water splitting to generate hydrogen fuel from water while tailoring the absorption to the visible range of the solar spectrum. Fundamental studies of charge separation on model systems such as CdS–Au and CdS–Pt nanorods along with advanced investigations of favored working conditions such as excitation regimes and alkaline environments for enhanced photocatalytic performance have been conducted and can set a basis for the future utilization of such and related systems in addressing “real-world” challenges. Additionally, HNPs have been applied as a new type of photoinitiator termed “quantum photoinitiator” for photopolymerization reactions in general and in 3D printing scenarios in particular as was demonstrated by CdS–Au nanorods, which were shown to enable photopolymerization of polyacrylamide. Photodynamic therapies and bioimaging based on HNPs are also of interest in this respect. Remarkably, HNPs such as Au–Cu_2–*x*_S and AuPt–CuS showed antitumor activity followed by a decrease in tumor volume under NIR irradiation in both in vitro and in vivo experiments. Section 4 discusses the emergent photocatalytic applications of chalcogenide-based HNPs.

While two decades of research have led us to an advanced stage in the synthesis of HNPs, understanding their properties, and developing their utilization as photocatalysts, there is a vast landscape of potential further developments and breakthroughs yet to be discovered. The outlook and perspective of such HNPs is presented in the closing section of the review, section 5.

## 2 Design Principles and Synthesis of Hybrid Semiconductor–Metal Nanoparticles

Over the last three decades, the synthetic pathways to prepare semiconductor–metal hybrid nanoparticles have vastly expanded and developed. These developments allowed the formation of various hybrid nanosystems composed of different material combinations.^3,12,14−16^ The specific material combination can dictate the physical, chemical, and optical properties of the resultant hybrid nanosystem. Often, the HNPs structure and morphology is also a consequence of this material combination, in which one substance promotes the other component’s growth or deposition parameters including orientation and selective sites, for example, due to its own structural properties such as facet reactivity, lattice constant, and surface defects.

An early example of HNPs was presented through the growth of metal islands on semiconductor nanoparticles (NPs), such as Au, Ag, Cu, and Pt growth on ZnO nanostructures^17^ or TiO_2_–Au and TiO_2_–Pt composites.^18−20^ However, control over the HNPs structural parameters such as the shape, size, and selective metal deposition became further achievable since the pioneering demonstration of site-selective Au metal growth on the apexes of CdSe nanorod structures.^1^ From that point, the diversity of semiconductors and metals used as building blocks to the formation of HNPs has been enriched extensively. A variety of transition metal chalcogenide have been used for the semiconductor component including CdS, ZnS, ZnSe, Cu_2_S, MoS_2_, MoSe_2,_ WS_2_, and WSe_2_, along with a selection of noble and transition metals including Au, Ag, Pt, Pd, Co, and Ni that have been utilized as the metallic component in the hybrid system.

**Scheme 2:**
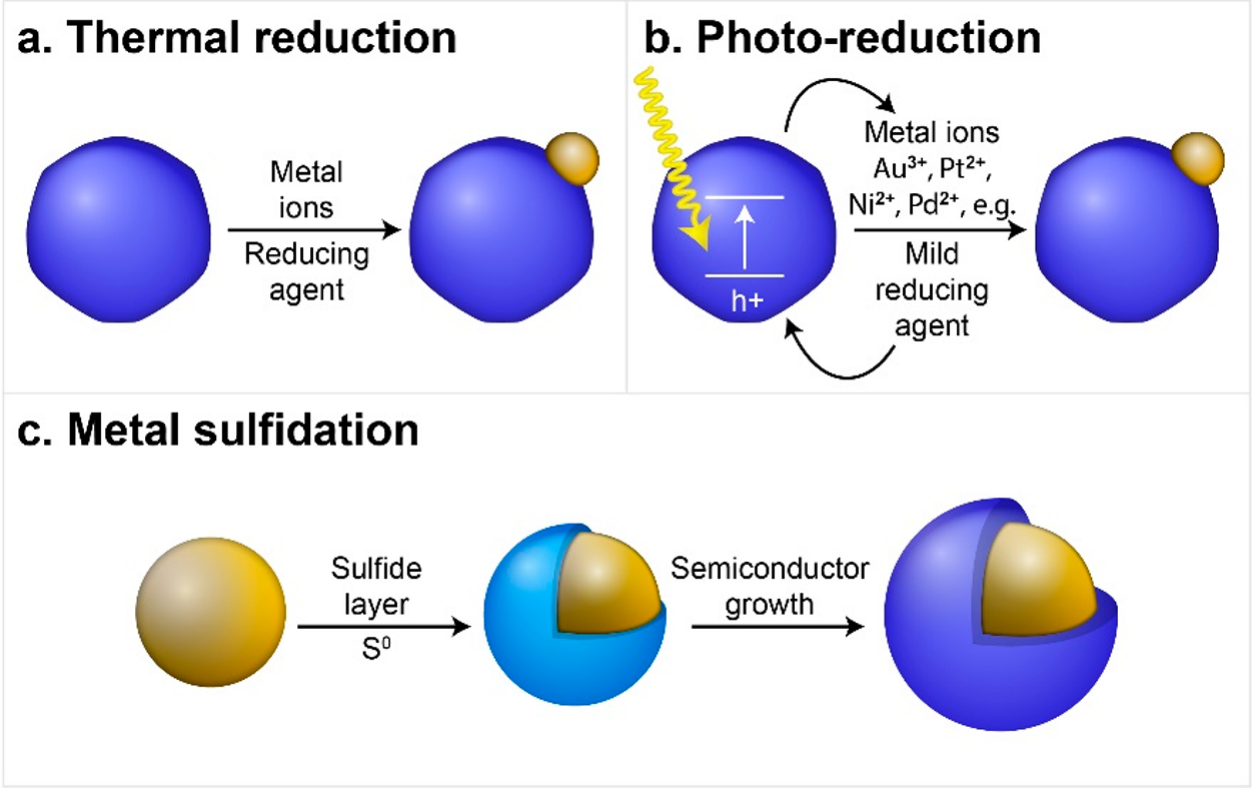
Different Synthetic Methods for Semiconductor–Metal HNPs Formation

In many cases, the desired specific property of HNPs, whether it is structural, physical, or chemical, dictates the synthetic strategy. Therefore, several synthetic routes have been developed exploiting the different characteristics of the two different materials, semiconductor and metal. Although physical deposition methods such as physical vapor deposition (PVD), pulsed laser deposition (PLD), and molecular beam epitaxy (MBE) have been reported and manifested in the literature, one of the most widely used controllable synthetic methods is chemical reduction deposition.^7,12^ As illustrated in Scheme 2, from direct heterogeneous deposition toward core/shell structures through direct heterogeneous nucleation for heterodimer nanostructures formation along with selective growth mechanisms based on the anisotropic material characteristics with or without assistance of light-induced photodeposition, these synthetic mechanisms allow deposition of metals on semiconductors or their incorporation into them and growth of semiconductors on metal seeds. Thermal or chemical reduction is typically obtained in the presence of organic molecular reducing agents, commonly alkyl amines such as dodecylamine and oleylamine, at various temperature conditions from room temperature to hot batch syntheses according the type of metal ions and reducing agents. The amine electron’s lone pair can reduce the nearby metal ions preferentially at the surface of the semiconductor nanocrystals (NCs), as will be elaborated in the next section (Scheme 2a). In addition, these reducing agents also take part in the colloidal stabilization of the formed HNPs. Formation of HNPs via photoreduction of metal ions is a remarkable manifestation of the HNPs synergetic properties. By exploiting the absorption and excitonic features of the semiconductor segment that lead to accumulation of free charges on the semiconductor surface, the metal ions can be reduced by it and form metal islands or segments in a controlled manner, including material composition and site-selective deposition (Scheme 2b). Additionally, formation of HNPs can be attained by partial transformation deposition (Scheme 2c). This strategy is based on the oxidation or sulfidation of the seed surface as an initial step to allow the deposition of different materials by introducing the desired organic precursors at the proper temperature conditions as will be discussed extensively in section 2.2.3.

### 2.1 Thermodynamic Aspects in Heterogeneous Deposition

**Scheme 3:**
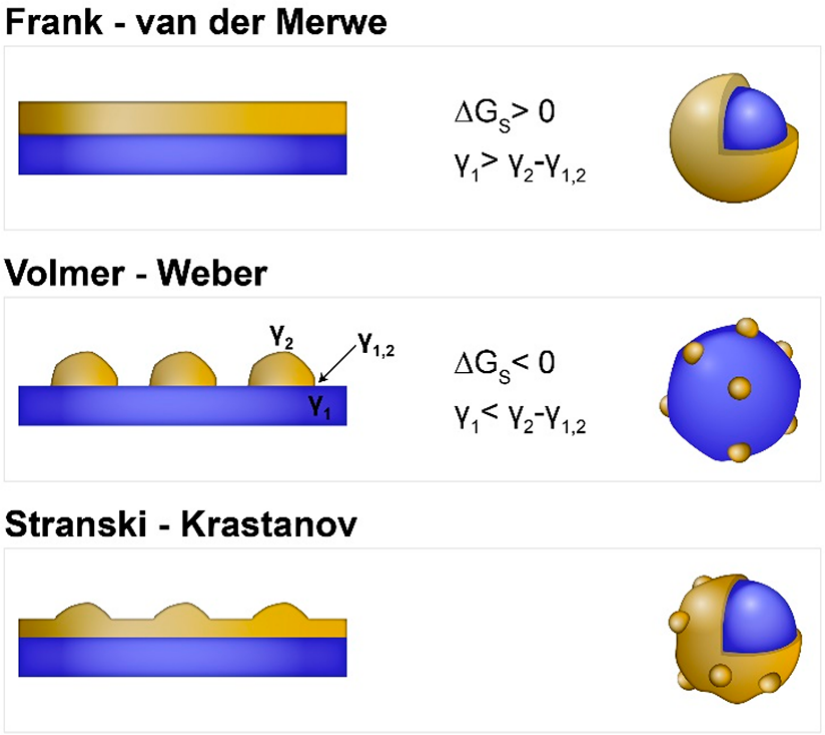
Illustrations of Possible Heterogeneous Deposition Modes for a Secondary Material That Is Deposited from the Respective Molecular Precursors onto a Preformed Seed Substrate of a Different Material Including Franck—van der Merwe, Volmer—Weber, and Stranski—Krastanov Regimes

Fundamentally, the underlying mechanism that promotes the formation of heterogeneous growth at the semiconductor–metal interface can be derived from the thermodynamic perspective of a similar macrosystem of thin film epitaxial growth over a crystal surface. The growth mode of a second material on a pre-existing substrate (first material) is formally dependent on the sign of the total Gibbs free surface energy function, Δ*G*_S_, that defines the heterogeneous deposition reaction and is given by^21,22^
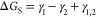
1where γ_1_ and γ_2_ are the surface energies associated with the respective materials (the solid/solution interfacial energies in the case of colloidal nanostructures in a liquid medium) and γ_1,2_ is the solid/solid interfacial energy. In a colloidal phase synthesis, the former two terms can be significantly influenced by adhesion of capping ligands, precursors, and solvent, while the latter depends on the bonding strength and degree of crystallographic compatibility of the concerned lattices. Hence, in the case of Δ*G*_S_ > 0, which is a result of a lower energy surface of the secondary material (γ_2_ < γ_1_) and/or a good crystallographic matching with the substrate (γ_1,2_ is small), a layer-by-layer deposition will probably take place (i.e., Frank—van der Merwe (FM) growth mode, Scheme 3, upper panel), resulting in a continuous and uniform coverage. When Δ*G*_S_ > 0, due to higher energy surfaces of the secondary material (γ_2_ > γ_1_) and/or significant lattice mismatch (γ_1,2_ is high), it will tend to deposit adopting the habit of a discontinuous island-like domain array (i.e., Volmer—Weber (VW) growth mode, Scheme 3, middle panel) to minimize of the overall interfacial area shared with the substrate. An additional route of heterogeneous growth is a combination of the two former mechanisms. First, a continuous film of the second material (up to several monolayers) can be deposited (wetting layer). The strain energy induced by the lattice mismatch between the two materials accumulates, and above a critical thickness, three-dimensional island growth will be favored to relieve the misfit strain, leading to an island-like formation (Stranski—Krastanov (SK) growth mode, Scheme 3, lower panel).

These different deposition routes can be realized via specific synthetic approaches. One of the most common strategies for the synthesis of heterogeneous nanoparticles in general and HNPs in particular is “seeded growth” implemented in solution phase, which may be considered as a derivative approach of traditional vapor-phase heteroepitaxial deposition. According to classical nucleation theory, the energy barrier for further growth on an already formed particle via heterogeneous nucleation is substantially lower than the activation energy for the formation of a new particle via homogeneous nucleation, as can be described by the following relationship
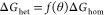
2where θ is the contact angle between the seed and the additional growth phase and the wetting function, *f*(θ), ranges between 0 (θ = 0°) in the case of heterogeneous nucleation and 1 (θ = 180°) for homogeneous nucleation. Note that the contact angle is influenced by the structural characteristics of both seed and deposited phases and the surface and interface tension at their boundaries.

In light of the above explanation, colloidal synthesis involving different organic stabilizers and other solution species, which affect the ultimate Gibbs free energy balance, along with kinetic factors such as solution supersaturation, various reactive precursor types, and different chemical diffusion conditions that may relieve the misfit strain provides a multitude of potential pathways to achieve an enormous diversity of HNPs with different morphologies and architectures.

### 2.2 Synthetic Methods

**Scheme 4:**
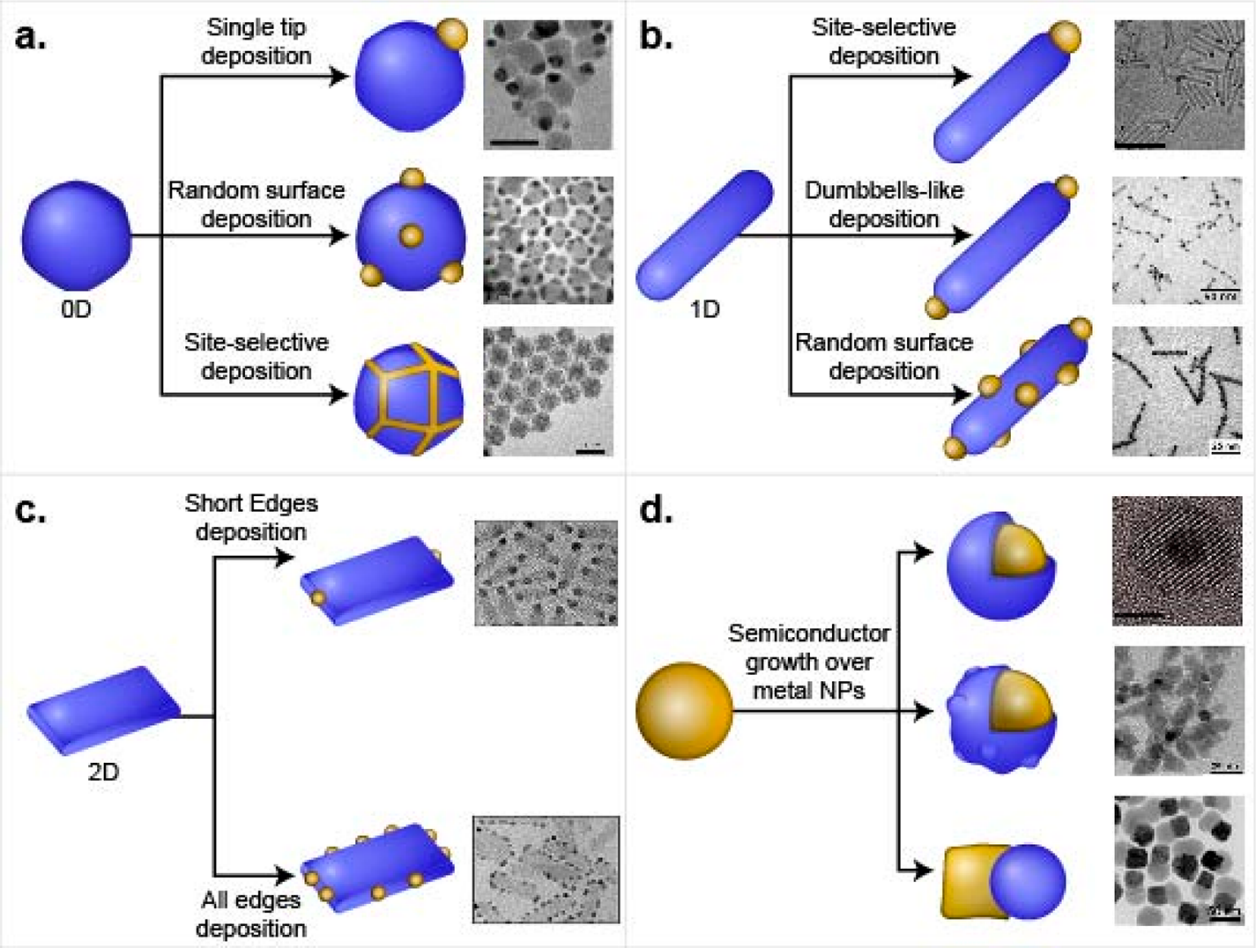
Synthetic Approaches for HNP Formation Schematic along with TEM images of selective metal deposition on (a) 0D, (b) 1D, and (c) 2D semiconductor NPs resulted in different HNPs architectures. (d) Selective deposition of semiconductor on metal NPs including Janus and core/shell morphologies.

The metal–semiconductor nanojunction can be formed by two strategies. As illustrated in Scheme 4, one way is to use semiconductor NCs as seeds to promote metal reduction deposition on its surface, and the second is semiconductor segment growth on metal NPs, which serve as seeds. In the next section, we will first focus on the HNP nanostructures achieved by semiconductor-seeded methods such as thermal and photoinduced procedures. The following section will review the metal-seeded approach.

#### 2.1.1 Chemical Reduction Deposition

**Figure 1:**
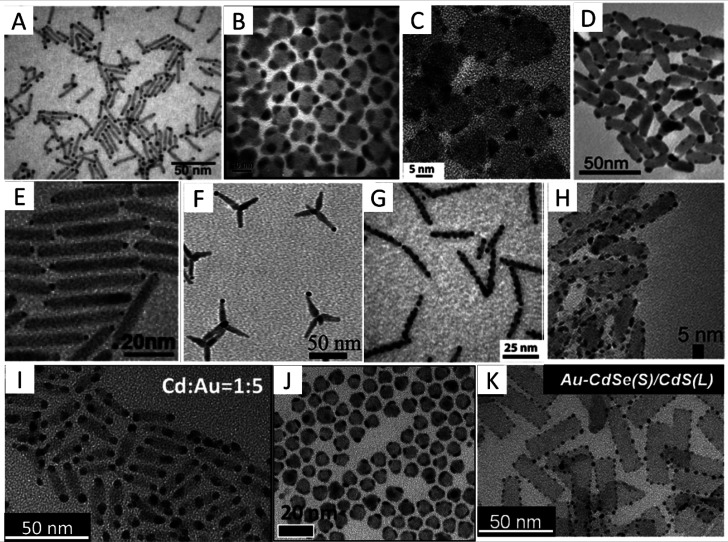
Chemical reduction deposition in the presence of DDAB and DDA surfactants. TEM images of various HNPs including (A) CdSe–Au nanodumbbells, (B) PbS–Au_4_ dots, (C) CZTS–Au cubes, (D) CdSe–Au NRs, (E) CdS–Au single-tipped NRs, (F) CdSe/CdS–Au tetrapods, (G) CdS–Au multiple random-decorated NRs, (H) CZTS–Au NRs, (I) CdSe–Au NPLs, (J) CdS–Au NPs, and (K) CdSe/CdS–Au NPLs. (A) Adapted with permission from ref (1). Copyright 2004 AAAS. (B) Adapted with permission from ref (24). Copyright 2004 American Chemical Society. (C) Adapted with permission from ref (25). Copyright 2014 American Chemical Society. (D) Adapted with permission from ref (149). Copyright 2021 American Chemical Society. (E) Adapted with permission from ref (32). Copyright 2015 Wiley. (F) Adapted with permission from ref (25). Copyright 2012 American Chemical Society. (G) Adapted with permission from ref (26). Copyright 2006 American Chemical Society. (H) Adapted with permission from ref (36). Copyright 2014 American Chemical Society. (I) Adapted with permission from ref (41). Copyright 2015 American Chemical Society. (J) Adapted with permission from ref (42). Copyright 2016 Royal Society of Chemistry. (K) Adapted with permission from ref (43). Copyright 2017 Wiley.

A general synthetic strategy for the formation of semiconductor metal HNPs in which metal salt precursors are reduced on the surface of presynthesized semiconductor nanoparticles was first demonstrated by Banin et al. for colloidal CdSe nanorods (NRs) with selectively deposited Au metal domains on their apexes.^1,23^ Typically, deposition of Au domains on various semiconductor materials is performed in organic phase solution at room temperature where the metal precursors are dissolved in this nonpolar environment with the assistance of surfactant molecules such as didodecyldimethylammonium bromide (DDAB), while the reduction occurs in the presence of a reducing agent, for example, dodecylamine (DDA), which can act also as stabilizer for the nanoparticles. Utilization of this method was expanded to a variety of HNPs including different semiconductor materials and shapes. This includes 0D structures such as PbS–Au dots^24^ and Cu_2_ZnSnS_4_ (CZTS)–Au cubes.^25^ Extensive work on synthetic Au decoration has been reported on quasi-1D structures including CdS–Au,^26−29^ ZnSe–Au,^30^ CdSe/CdS–Au,^31−34^ CdTe–Au,^35^ and CZTS–Au^36^ nanorods and tetrapods. More recently, with the synthetic development of 2D nanoplatelet-like semiconductor structures (NPLs), their semiconductor–metal hybrid forms were generated also. This includes CdS–Au,^37,38^ CdSe–Au,^39−41^ and CdSe/CdS–Au.^42,43^ Various HNPs with different morphologies and material compositions which were synthesized via this approach are shown in Figure 1.

**Figure 2:**
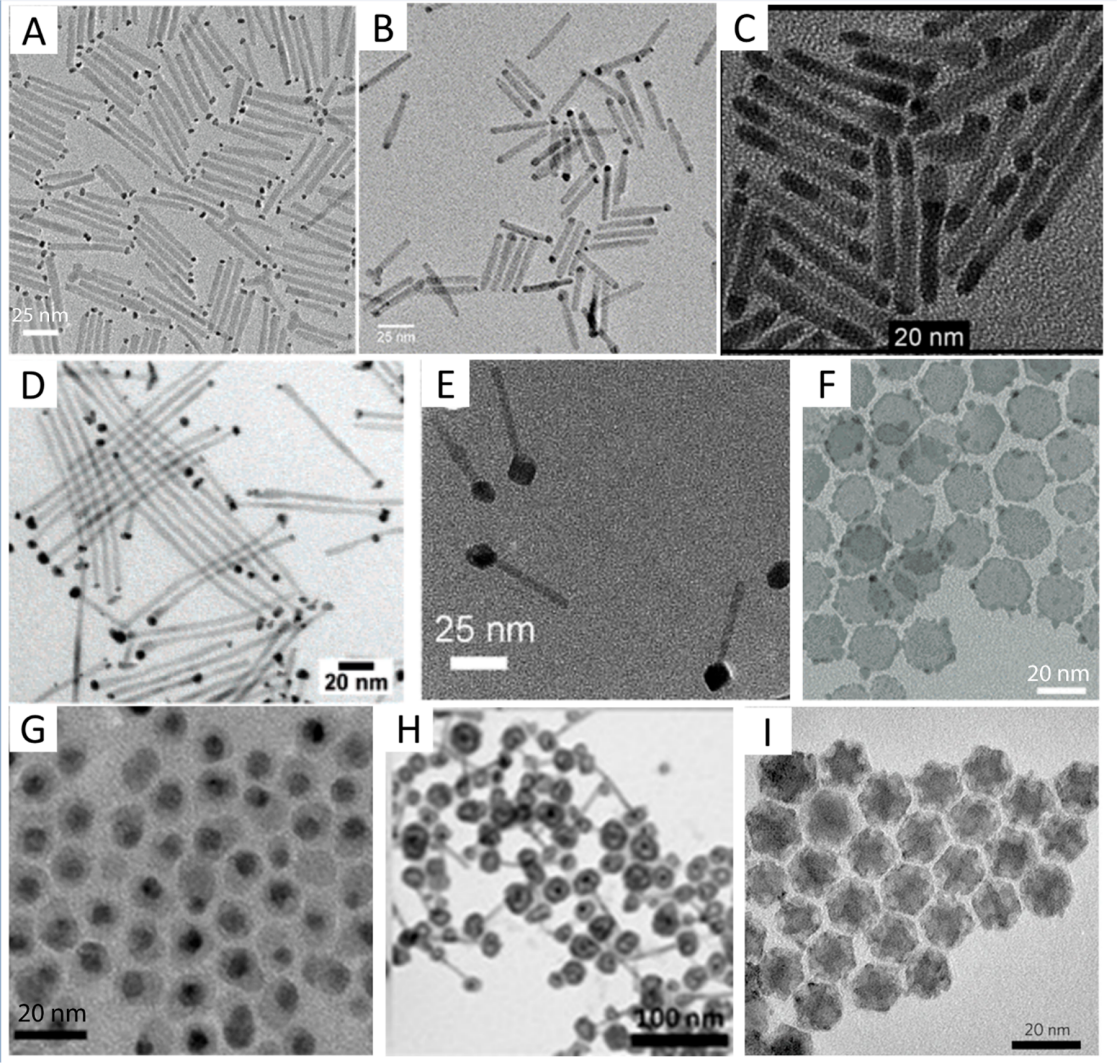
Chemical reduction deposition in the presence of oleylamine surfactants. TEM images of various HNPs including (A) CdSe/CdS–Pt NRs, (B) CdSe/CdS–Ag NRs, (C) CdS–PdS NRs, (D) CdS–PtCo binary metal-tipped NRs, (E) CdSe/CdS–Ni NRs, (F) CuInS_2_–Pt hybrids, (G) CZTS–Pd hybrids, (H) CdSe/CdS–Pt/Co core/shell-tipped NRs, and (I) Ru nanoinorganic caged Cu_2_S nanoparticles. (A) Adapted with permission from ref (239). Copyright 2016 American Chemical Society. (B) Adapted with permission from ref (51). Copyright 2021 American Chemical Society. (C) Adapted with permission from ref (50). Copyright 2011 Wiley. (D) Adapted with permission from ref (44). Copyright 2008 American Chemical Society. (E) Adapted with permission from ref (49). Copyright 2016 American Chemical Society. (F) Adapted with permission from ref (47). Copyright 2015 American Chemical Society. (G) Adapted with permission from ref (53). Copyright 2017 American Chemical Society. (H) Adapted with permission from ref (55). Copyright 2012 American Chemical Society. (I) Adapted with permission from ref (57). Copyright 2010 Nature Publishing Group.

Modification of this procedure while using oleic acid and oleylamine as stabilizing and reducing agents, respectively, along with higher reaction temperatures allowed for the deposition of additional metals on similar semiconductor components. Figure 2 shows TEM images for some of the following HNPs examples. Habas et al. demonstrated this synthetic pathway via solvothermal deposition of Pt domains on the apexes of CdS NRs.^44^ In addition, binary metal tips were also reported including PtNi and PtCo, where both metal precursors are present during the reduction reaction. A similar synthetic route was used to deposit Pt nanodomains on the apexes of heterostructure semiconductors such as CdSe/CdS,^45^ ZnSe/CdS, and ZnTe/CdS nanorods^46^ and CuInS_2_ nanoparticles.^47,48^ Additionally, other metals were epitaxially deposited under similar conditions including Ni.^49^ The reactivity of the semiconductor rod apexes was also exploited for the deposition of PdS.^50^ Dong et al. reported on the formation of CdSe/CdS–Ag NRs by reducing Ag–trioctylphosphine (TOP) complexes with oleylamine,^51^ thereby avoiding common cation exchange yielding CdSe/CdS–Ag_2_S heterostructures based on hard and soft (Lewis) acid and base theory.^52^ In the presence of TOP, which is a soft base, complexes consisting of a soft acid like Ag ions would preferably form while hampering the driving force of a hard acid, Cd ions, to migrate outside of the crystal structure. Reduction by oleylamine was also used for deposition of presynthesized metal nanoparticles such as Pt or Pd, which were used as seeds on CZTS nanoparticles to form core/shell and heterodimer architectures.^53^

**Figure 3:**
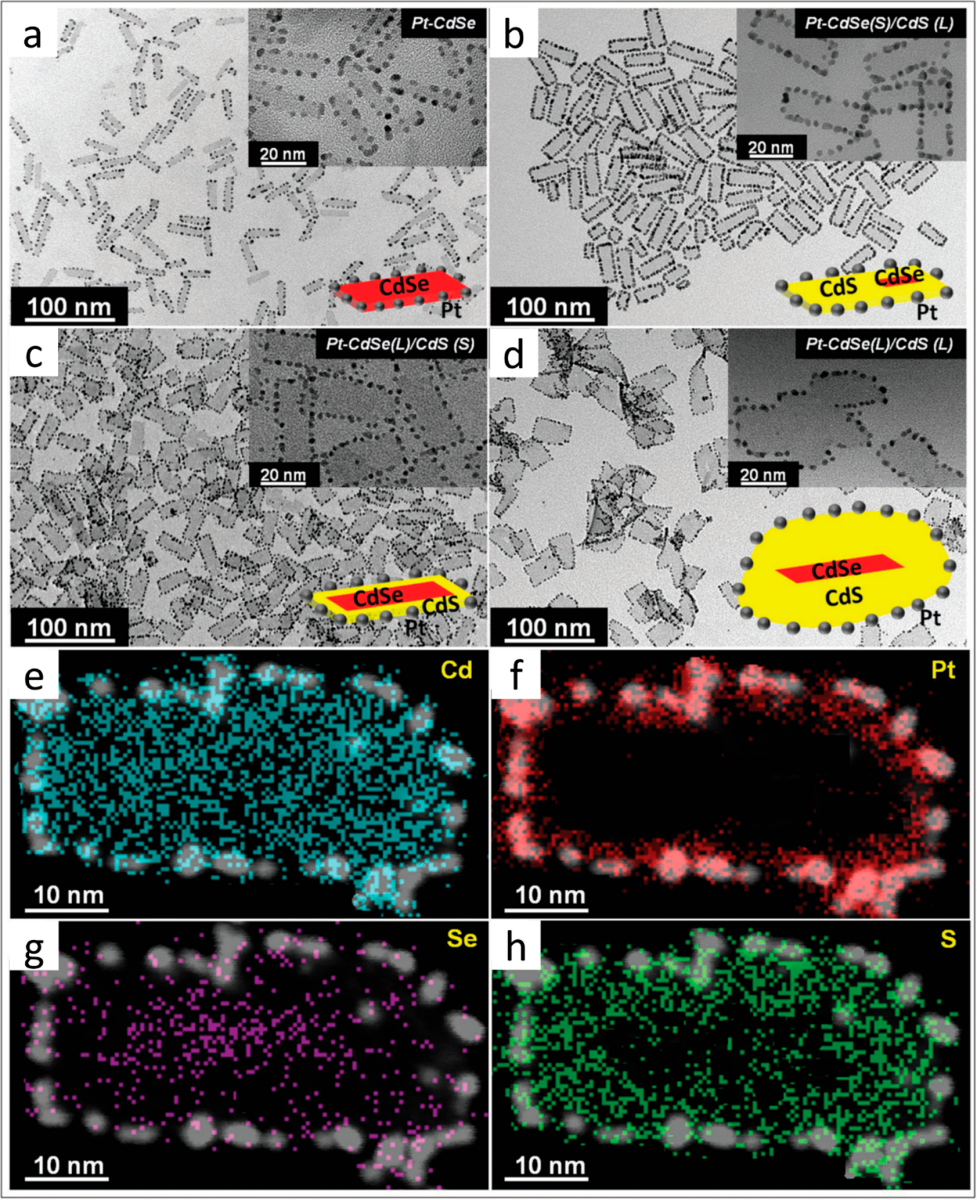
Pt-decorated CdSe and CdSe/CdS NPLs with variable CdSe core and CdS crown dimensions. TEM images of (a) Pt on CdSe, (b) Pt on CdSe(S)/CdS(L), (c) Pt on CdSe(L)/CdS(S), and (d) Pt on CdSe(L)/CdS(L) with schematic illustrations of the respective systems. (Insets) Higher resolution TEM images confirming the presence of quasi-spherical Pt domains around all of the edges of the NPLs at regular distances from each other. (e–h) HAADF-STEM images averaged with STEM-EDXS mappings for the elements Cd, Pt, Se, and S of Pt-decorated CdSe(L)/CdS(L) NPLs. Adapted with permission from ref (43). Copyright 2017 Wiley.

Cobalt deposition on the tips of CdSe nanorods was first achieved by reduction of [Co(η_4_-C_8_H_12_)(η_3_-C_8_H_13_)] in toluene with lauric acid and hexadecylamine under H_2_ atmosphere and elevated temperature.^54^ Pyun et al. presented cobalt metal heterogeneous growth on a pre-existing Pt tip serving as a substrate to allow the growth of a second metal phase^55^ while applying carboxylic acid-terminated polystyrene as a reducing agent used previously for cobalt deposition reactions.^56^ A unique metal decoration of Ru and Rh metallic frames over Cu_2_S nanoparticles was achieved by temperature-controlled thermal reduction of Ru or Rh acetylacetonate (acac) with octadecylamine in octyl ether or diphenyl ether/dichlorobenzene solution, respectively.^57,58^ Solvothermal formation of semiconductor–metal HNPs was achieved also in the absence of additional reducing agents and surfactants as reported by Cozzoli and co-workers. CdS–Co and CdSe/CdS–Co NRs were obtained at high temperature (240 °C) in octadecene and Co_2_(CO)_8_ as the metal precursor.^59^ Although chemical reduction of metal precursors is mostly reported in organic solutions, its feasibility was proven also in aqueous environments. For example, CdSe–Pt nanorods and nanonets were obtained under different pH values of aqueous solutions.^60^ Additionally, aqueous condensation of Pd^II^ onto CdS NRs was reported by Shemesh et al.^50^ As Au growth was utilized on 2D nanosystems, implementation of the described oleylamine-assisted synthetic route was also conducted on NPLs. Typically, Pt or Pd metal domains were deposited on the edges of homogeneous or heterostructure semiconductor NPLs resulting in CdSe–Pt,^41^ CdSe–Pd,^41^ CdS–Pt,^37,61^ and CdSe/CdS–Pt^43^ (Figure 3). Recently, a modified reducing procedure for Au deposition on nanoplatelets was presented by Mews et al. where CuS–Au hybrid NPLs were obtained in the presence of oxalic acid and oleylamine.^62^

#### 2.2.2 Photoreduction Deposition

Harnessing the photophysical properties of the semiconductor component allows the development of an additional important synthetic avenue to form HNPs via light-induced metal deposition. Following irradiation of the semiconductor, the absorbed photon generates the formation of an excited charge carrier within the semiconductor, namely, an electron–hole pair. These charge carriers, typically electrons, may accumulate at the defect site or preexisting metal domains through a suitable relaxation route. This is then followed by site-selective reduction of dissolved metal ions in the semiconductor surroundings on the surface of the semiconductor segment or at the metallic seeds. Commonly, besides the participating surfactants in the reaction that are accountable for reducing and stabilizing the nanoparticles, addition of a hole scavenger such as ethanol is required to efficiently exploit the excited electrons and avoid e–h recombination processes. This strategy holds an advantage of permitting site-selective and controllable deposition as will be described briefly next and discussed in detail in section 3.1.

**Figure 4:**
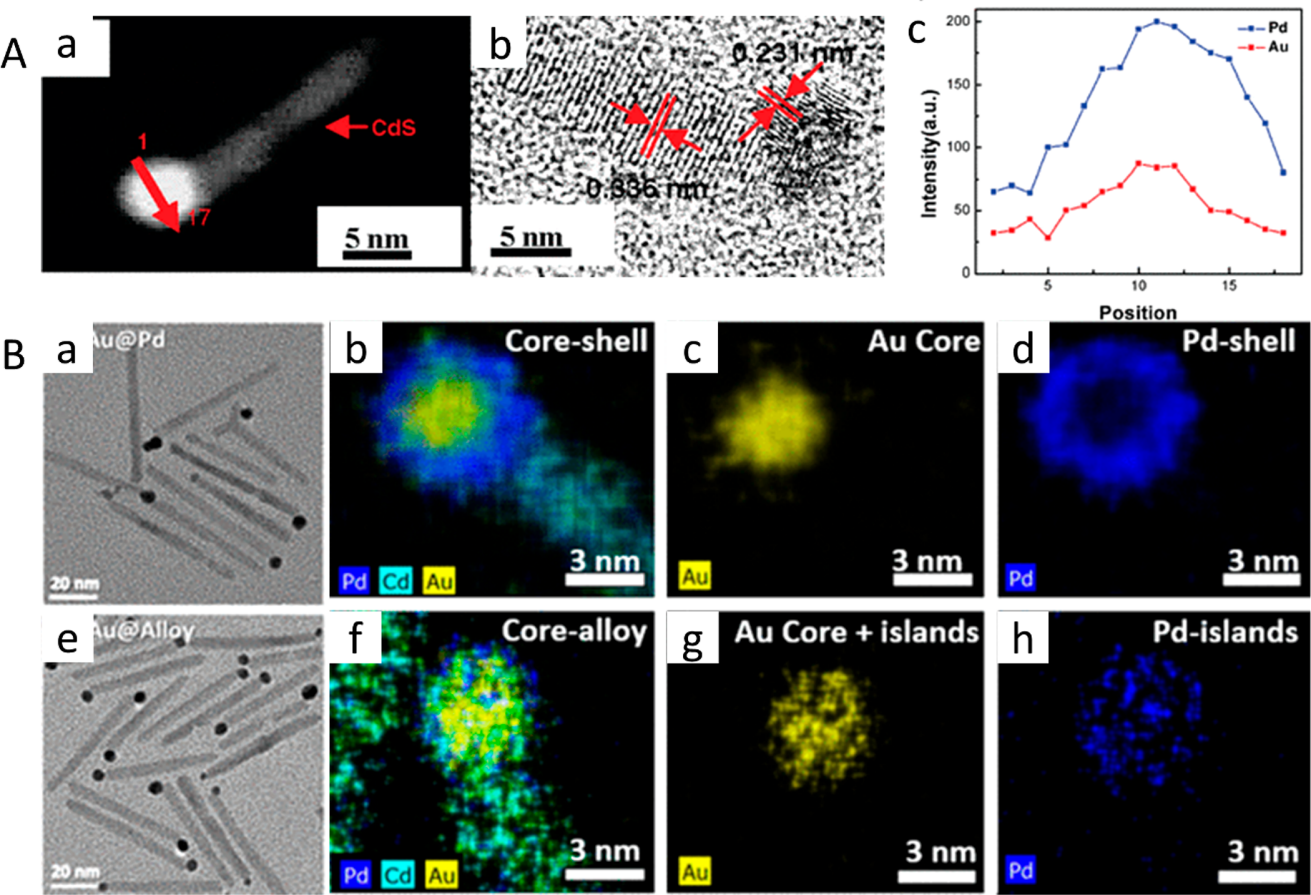
Bimetal deposition. (A) (a) HAADF-STEM image showing a homogeneous material composition at the spherical tip. (b) HRTEM image of a Pd–Au–CdSe-seeded CdS rod. Discernible lattice spacings of 0.231 and 0.336 nm are attributed to the PdAu {111} plane and CdS {002} plane, respectively. (c) Corresponding EDX line scan across the spherical tip of the matchstick structure. (B) TEM images and corresponding EDS elemental mappings of the bimetallic Au/Pd core/shell (a–d) and Au–Pd alloy tips (e–h). (A) Adapted with permission from ref (66). Copyright 2010 American Chemical Society. (B) Adapted with permission from ref (67). Copyright 2015 American Chemical Society.

The first demonstrations of photodeposition on metal–chalcogenide semiconductor NCs were presented for CdS–Pt and CdSe/CdS–Pt NRs^63^ along with Au growth on similar semiconductor structures, allowing different metal deposition morphologies and architectures.^27,64,65^ While photodeposition of Au domains contains similar metal precursors and surfactants, typically, AuCl_3_, DDAB, and DDA, respectively, Pt light-induced growth required different reactive precursors in comparison to the thermal deposition method. Pt domains were obtained in the presence of (1,5-cyclooctadiene)dimethylplatinum(II) as the platinum source and an excess of a tertiary amine (such as triethylamine and diisopropylethylamine). Combination of seeded growth principles and photodeposition allowed for size control and bimetallic deposition. Since the formed metal island serves as an electron sink for the photoexcited electrons, the reduction of the nearby metal ions takes place on the preexisting metal domain. Li et al. reported on Pd/Au alloyed metal-tipped CdSe/CdS NRs, as can be seen in Figure 4A. Following UV excitation of CdSe/CdS–Au HNPs in the presence of PdCl_2_ and additional surfactants such as tetraoctylammonium bromide (TOAB) and DDA, an increase in the average diameter is observed due to Pd deposition.^66^

Further examples of different metal and bimetals deposition were obtained via photodeposition with slightly modified procedures, mostly by introducing different surfactants and various semiconductor/metal ion ratios. CdS–Au NRs with tunable metal domain sizes were formed via light-induced Au growth over activated CdS nanorods with a small metal tip (thermally deposited under dark conditions).^28^ Formation of CdSe/CdS–Pd along with combined deposition of Au and Pd as the core/shell and alloyed tipped HNPs was presented by Bar-Sadan and co-workers (Figure 4B).^67^ Au and the Pd/Au domains in both textures showed spherical structures, while Pd metal deposition resulted in a match-like morphology with a quasi-rectangular shape. Similarly, the compositions of Au and Pt with both core/shell and Pt-decorated Au domains were achieved via photodeposition on CdSe/CdS HNPs.^68^ Aqueous photodeposition was also reported, achieving CdS–Pt,^69^ CdSe/CdS–Au,^70^ and CdS–Ni nanorods^71^ and nanoparticles^72,73^ following phase transfer performed via ligand exchange of the native organic ligands on the surface of the semiconductor component. In the latter case of Ni deposition, metal growth on the semiconductor segment was obtained “in situ” during the photocatalytic reaction of hydrogen generation.

Other semiconductor structures were shown to allow photoreduction of metal islands including Ni over CdS nanosheets^74^ and Au on CdSe/Cd_0.5_Zn_0.5_S core/shell NPLs.^39^ Recently, toward adopting greener synthesis protocols, photodeposition in ionic liquids in which additional surfactants are redundant has been developed for Pt, Au, and Ag deposition on CdSe–CdS NRs,^75^ achieving comparable results to the common amine-capped colloidal synthesis in organic medium. Another recent approach combines flow reaction synthesis with photodeposition to achieve controllable upscale of the HNPs syntheses. Continuous processes have been already utilized for different semiconductor and metal nanocrystals; nevertheless, Cohen et al. presented this kind of implementation together with light-induced reaction to achieve CdSe/CdS–Au, ZnSe–Au, and novel ZnSe–Pt NRs.^76^

#### 2.2.3 Direct Heterogeneous Deposition via Partial Transformation

**Figure 5:**
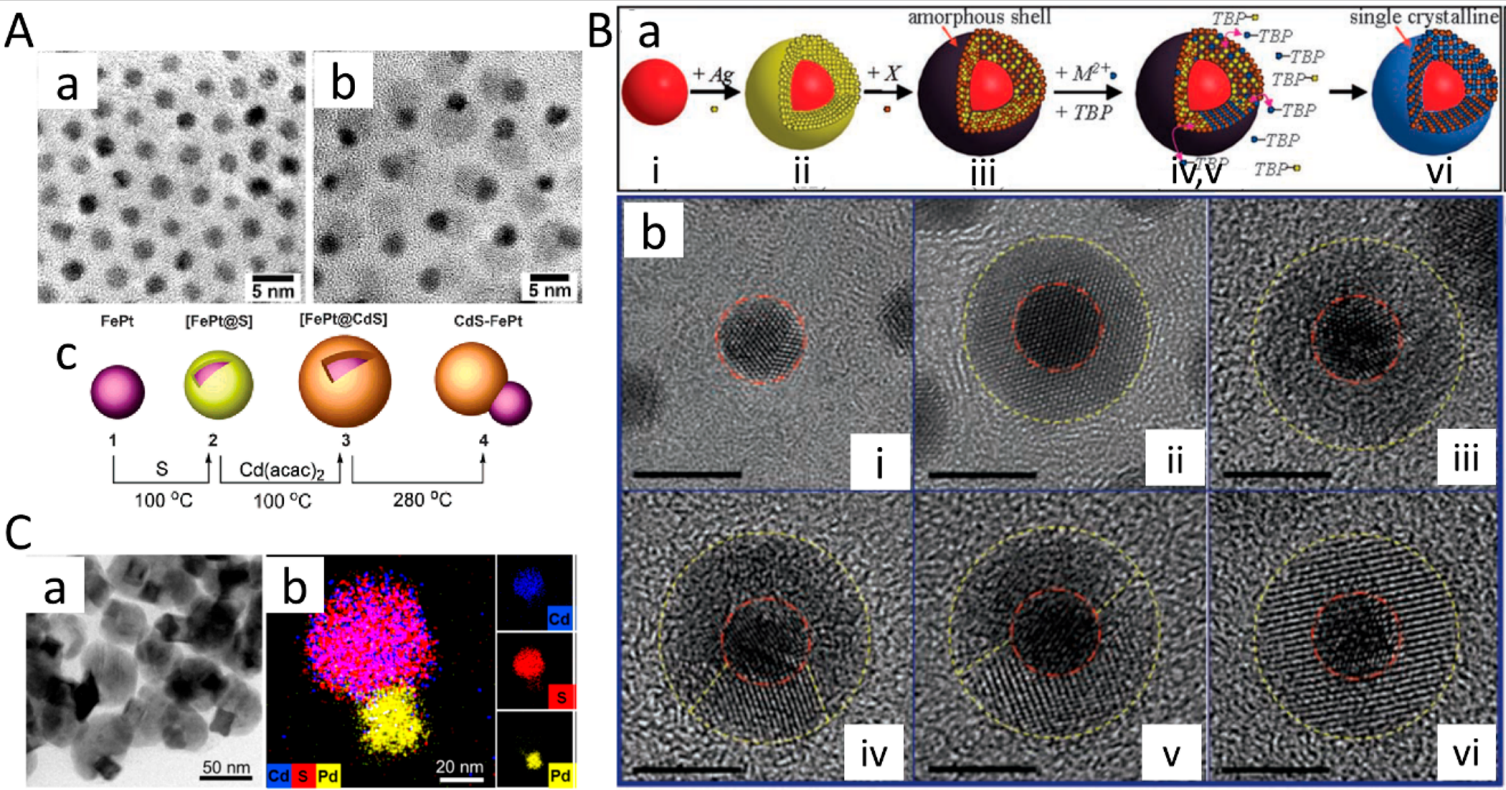
Direct heterogeneous deposition and deposition via partial transformation. (A) TEM and HRTEM images of (a) FePt NPs before deposition and (b) Janus CdS–FePt HNPs. (c) Schematic of the synthetic route. (B) (a) Scheme of the nonepitaxial growth process; (b) series of HRTEM images of different synthetic stages of Au–CdS growth. Scale bar, 5 nm. Red and yellow dashed lines are guides for the eye, distinguishing the core and shell boundaries, respectively. (C) (a) TEM and (b) HAADF-STEM-EDS elemental mapping images of Janus Pd–CdS nanocubes. (A) Adapted with permission from ref (77). Copyright 2004 American Chemical Society. (B) Adapted with permission from ref (83). Copyright 2015 AAAS. (C) Adapted with permission from ref (84). Copyright 2014 American Chemical Society.

Among the first suggestions for semiconductor–metal HNP formation was partial chemical transformation of the seed material, typically the metal component. During this procedure, the metallic surface is either oxidized or sulfidized to obtain a thin wetting layer of metal–oxide or metal–sulfide for a consecutive growth of the desired semiconductor phase. This strategy was exploited by Gu et al. to generate FePt–CdS heterodimer HNPs in a facile one-pot synthesis (Figure 5A).^77^ In this procedure, as illustrated in Figure 5A-c, first, an amorphous CdS shell is deposited over the FePt core bimetallic nanoparticles via sulfidation followed by metal ion and stabilizing ligand precursors addition at 100 °C. Next, the segregation of the metallic core from the semiconductor shell to obtain the heterodimer took place following additional heating to 280 °C in which the crystallization of the CdS component and a dewetting process took place. Similar control utilizing the reaction temperature was observed by Xu and co-workers upon introducing chalcogens following oxidation of Cd(acac)_2_ to create a CdO shell over the FePt nanoparticles surface. A lower reaction temperature (258 °C) resulted in core–shell structures, while higher temperatures (285–300 °C) promoted heterodimers formation.^78^ Further control over this synthetic approach was achieved through a moderate temperature ramp and different reaction times as established by Parak and co-workers. Moreover, this procedure was expanded to form additional classes of hybrids that comprise a FePt component and a II–VI or IV–VI semiconductor domain including CdS, ZnS, PbS, and CdSe.^79^

The components of the semiconductor–metal HNPs can also be predetermined by the choice of the seed composition. Schaak et al. demonstrated the formation of Au–Cu_2_S HNPs by introducing sulfur precursors in oleylamine to AuCu-alloyed nanoparticles under constant air bubbling.^80^ Similar approach of sulfidation in the presence of oleylamine at high temperature was demonstrated on PtCu-alloyed NPs, allowing the formation of Pt–CuS HNPs starting from either a Cu-seeded or a Pt-seeded NPs.^81^ The oxidative environment was necessary to activate the reaction by forming an oxide surface or intermediate. A general strategy for synthesizing binary and ternary HNPs based on sulfidation of the metallic seed was reported by Shi et al., allowing the formation of Au–PbS and Au–PbSe with dumbbell and tripod architectures.^82^

An additional common pathway is the use of Ag deposition on metallic nanoparticles as a preliminary step toward semiconductor–metal HNPs generation. This approach was exploited to produce a crystalline semiconductor shell over a metallic Au core based on the Lewis acid–base reaction mechanism.^83^ Sulfidation of the Ag layer created a Ag_2_S amorphous matrix that allowed the growth of the semiconductor lattice independently of the metallic core and thus circumventing the lattice mismatch between the two components, as illustrated in Figure 5B-a. Figure 5B-b presents the different synthetic stages of Au–CdS growth. The process starts by the synthesis of core metal NPs (i) through a soft Lewis metal deposition layer (ii) and formation of an amorphous structure (iii). The final step is a cation exchange reaction in the presence of a soft base (tributylphosphine) that gradually expels Ag ions from the shell (iv, v, and vi). The final composition of the semiconductor shell can be controlled by a cation exchange reaction with the desired metal cation to form a metal–sulfide component as was demonstrated for Au–CdS, Au–CdS_1–*x*_Se_*x*_, and Au–CdS+PbS.

This strategy was generalized for different noble metals (Au, Pd, and Pt), and improved control of Ag growth on metallic seeds was gained by addition of an appropriate amount of S^2–^ ions. In this manner, sulfur ions assist to manipulate the reduction kinetics of Ag^+^ ions, which results in the growth of Ag predominantly on one side of the preformed metallic nanocubes.^84^ The metallic heterodimers were used as a platform for the formation of metal–CdS HNPs through successive steps of sulfidation and cation exchange as described above (Figure 5C). Modification of this synthesis by Zhao et al. allowed for structural control and the formation of heterodimers instead of core–shell structures. An increase of the crystallinity of the Ag_2_X (X = S, Se, Te) shell as well as a higher reaction temperature of the cation exchange reaction led to a larger phase separation between Au and CdX to reduce the interfacial and grain boundary energies.^85^ In addition, Zhao and co-workers demonstrated the formation of Ag– and Ag_2_S– (or Ag_2_Se−) CdS, ZnS, MnS, and CdSe hybrid nanorods via controlled sulfidation (or selenidation) of Ag NPs.^86^

Different morphologies, architectures, sizes, and shapes are available depending on the surface coating, reaction temperature, metal type, precursors, and concentrations, as will be described in detail in the next section.

### 2.3 Synthetic Control over Structural Properties of Hybrid Semiconductor–Metal Nanoparticles

As was described in the former section and previously reviewed extensively in the literature,^2,7,14,87−89^ several synthesis mechanisms have been developed for the growth of HNPs. Applying one or a sequential combination of these strategies allows for good control over the structural characteristics of the HNPs in terms of their morphology and composition, which ultimately leads to different chemical and physical properties.^90^ This section focuses on controllable synthesis and structural characterization of different semiconductor–metal hybrid nanosystems. This includes site-selective deposition, tunable size and shape, and a variety of material compositions of both the semiconductor and the metal segments.

#### 2.3.1 Site-Selective Deposition

Selective deposition of either components of the semiconductor–metal HNPs may be dictated by the morphology and crystal structure of the semiconductor or metal phase. The crystal morphology and surface capping induce different chemical reactivity for different facets of the semiconductor nanocrystal, which can lead to specific growth of the second-phase material on the more reactive facets of the nanocrystals of the first-phase material. The architecture of site deposition can be determined through various synthetic approaches. Both thermal deposition and photochemical reduction methods can be used, resulting in different deposition patterns dependent on surface coating, reaction temperature, metal type, precursors, and concentrations.

A first demonstration of site-selective deposition dictated by the anisotropically shaped crystal structure was reported by Mokari et al., presenting Au single-metal domain deposition on the apexes of CdSe and CdS nanorods and tetrapods.^1,23^ In principle, the preferential growth on the tip sites, in this case, is originated from the enhanced reactivity of the (101) or (001) terminal facets of the semiconductor crystal leading to a lower free energy barrier for the heterogeneous nucleation on these sites.^91^ Furthermore, a transition from two tips to a single tip was observed and ascribed to an electrochemical Ostwald ripening mechanism stabilizing the larger single tip preferably over two tips.^23^ In addition, a controlled site-selective deposition where the metal precursor concentration acts as a knob for different site deposition resulted in the formation of single-tipped CdSe/CdS–Au nanorods along with dumbbell-like structures and body decoration on the semiconductor surface with increased Au concentrations.^92^ This control was attributed to the hierarchy of the semiconductor facets reactivities where the sulfur-rich nature of one apex promotes initial deposition of a single Au domain due to strong Au–S interactions. With the addition of metal precursors, deposition on the less reactive Cd-rich apex occurred. At saturated concentration conditions, spontaneous growth over defect sites on the surface of the semiconductor structure was observed.

**Figure 6:**
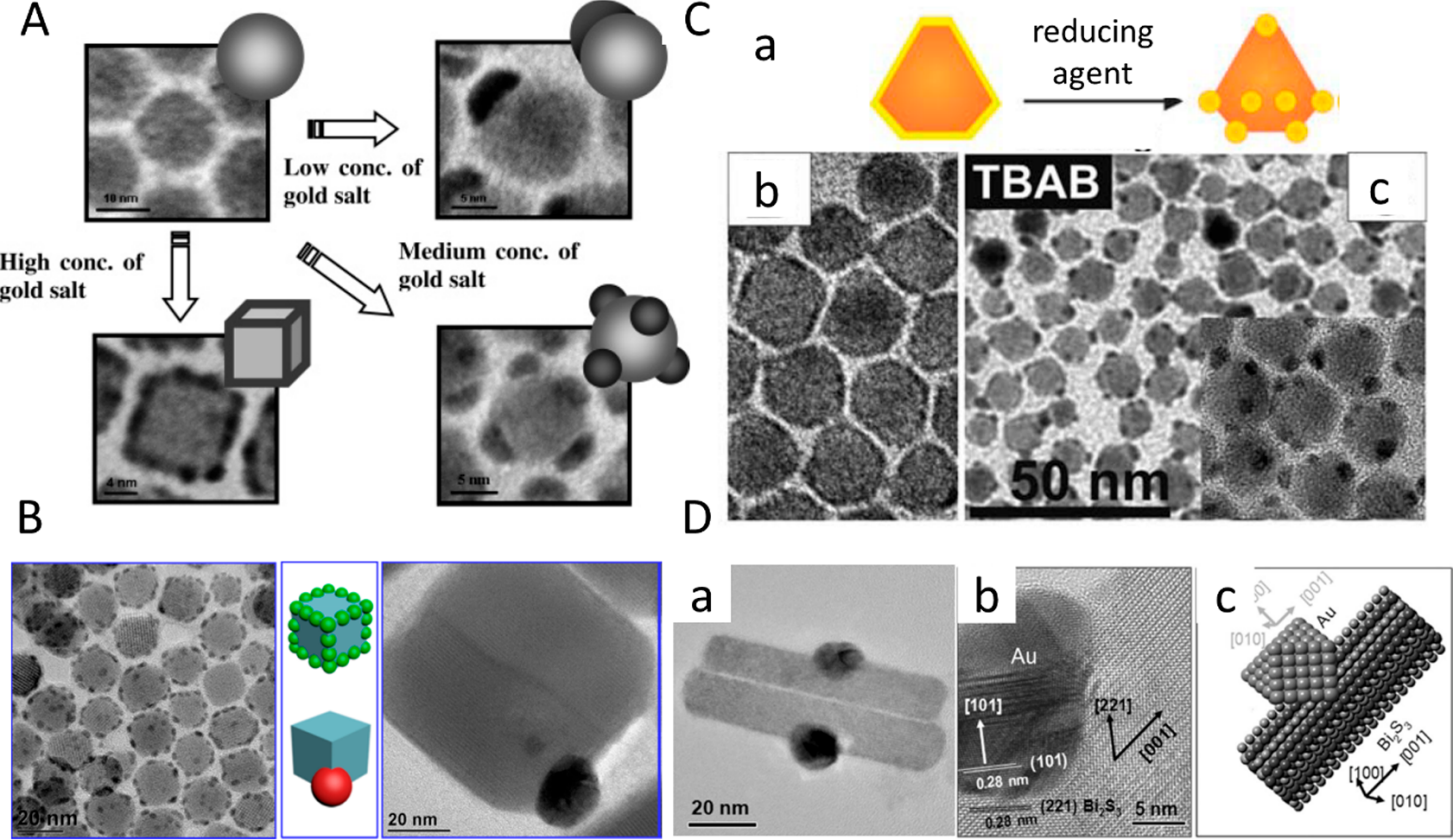
Site-selective chemical deposition. (A) Schematic of preferential deposition of Au sites on PbS NPs as a function of Au ions concentration. (B) TEM and HRTEM images showing semiconductor size-controlled decoration of cube-shaped Au–SnS heterostructures with multiple metal domains and a single site for small and large SnS cubes, respectively, along with their complementary scheme. (C) (a) Schematic of Au growth process forming the Au shell to several metal sites in the presence of strong reducing agents. TEM images: (b) enlarged image of CdSe NCs with an Au shell and (c) CdSe NCs after their incubation with Au–DTAB/OA and further addition of TBAB–DTAB solution. (D) TEM and HRTEM images of (a) Au–Bi_2_S_3_ hybrid and (b) the heterojunction with labeled planes. (c) Atomic model of a typical Au–Bi_2_S_3_ hybrid. (A) Adapted with permission from ref (24). Copyright 2006 American Chemical Society. (B) Adapted with permission from ref (95). Copyright 2014 American Chemical Society. (C) Adapted with permission from ref (97). Copyright 2010 Royal Society of Chemistry. (D) Adapted with permission from ref (98). Copyright 2014 Wiley.

Similar dependence on the polarity of crystal facets was observed for PbS–Au HNPs.^24^ The deposition of Au occurred favorably on PbS facets with the highest reactivity. In the reported cubic crystal system, the growth rates on common crystal planes as deduced formerly for various NCs can be ordered as [111] > [110] > [100].^93,94^ This suggests that Au would preferentially grow on the {111} facets. Because of the different polarities of the (111) and (111) facets, only four of the {111} facets were deposited and the PbS–Au_4_ (4 metal domains in average) nanostructure was formed. At high Au precursor concentration, gold would saturate all of the {111} facets and PbS–Au_8_ (eight metal domains in average) was formed. At an even higher Au precursor concentration, a gold-crowned cubic-shaped heterogeneous nanostructure was achieved. This evolutionary metal deposition as the metal precursor concentration increases is illustrated in Figure 6A.

An additional example of the semiconductor NCs templating the Au site deposition is manifested by Au deposition on SnS nanocubes of different sizes. Reducing HAuCl_3_ in the presence of small nanocubes (<25 nm) in aqueous solution resulted in decorated SnS–Au HNPs with sub-5 nm sized metal domains. However, a similar reduction reaction in the presence of large SnS nanocubes (>50 nm) formed large single-metal domain growth on the corner of the nanocubes and a heterodimer structure (Figure 6B).^95^ Thus, the different sizes of SnS promoted different reaction routes. The decorated form that is only observed for small sized SnS is governed by the minimum hydrophobic surface, which is easier for penetration into the aqueous phase, and random metal reduction is observed. However, larger sized SnS nanocubes have a larger hydrodynamic radius; therefore, its dispersity in aqueous media is poor. Hence, Au deposition is slow and thermodynamically dependent, resulting in selective deposition on favorable facets. In the case of SnS, the [021] polar axis of SnS allows zero lattice mismatch, which acts as the driving force for the specific attachment of the metallic Au on the S end sites, and this also leads to the epitaxy along the (131) plane of SnS with (111) of Au.

Another example of the morphology dependence was demonstrated for cone-like CdSe/CdS tetrapods, which exhibited increased selectivity toward single Au-tipped deposition as a result of the tapered arm structure, in comparison to regular tetrapods with cylinder-like arms.^34,96^ This tendency for tip growth is ascribed to an enhanced intraparticle electrochemical Ostwald ripening process due to the presence of more surface atoms in the cone-like structure in comparison to the cylindrical morphology.

Other shapes were found to dictate different hybrid architectures. Core/shell structures of CdSe–Au dihexagonal pyramidal HNPs were formed following mild AuCl_3_ reduction.^97^ This shell growth is attributed to the lower ligand density on the surface of the pyramidal nanocrystal in comparison to rod structures. Hence, more surface is accessible to the metal precursors to be deposited and forms a continuous shell. Further addition of a strong reducing agent or e-beam irradiation was reported to transform the metal shell to multiple isolated metal domains on the nanocrystal surface, as shown in Figure 6C.

Other methods of controlling the site and the morphology of the metal deposition are extensively reported in the literature. Pradhan and co-workers exploited the lattice mismatch of Au with Bi_2_S_3_ (ca. 2.81%) to form unique HNPs with Au nanoparticles positioned at the center of Bi_2_S_3_ NRs (Figure 6D).^98^ The authors attributed this morphology formation to the ability of Au particles to catalyze and enhance the rate of the 1D agglomeration along the [001] direction of the Bi_2_S_3_ nanoparticles which possess minimized lattice mismatch with the metal. Recently, Boldt et al. reported selective metal growth of different metals (Pt, Pd, Au) on semiconductor nanorod structures with enhanced selectivity for metal tellurides over the lighter chalcogenides.^99^ CdSe/CdS NRs with CdTe tips showed specific metal growth over the CdTe segment with suppressed body decoration over the nanorod surface. This behavior was attributed to a trap-mediated nucleation mechanism, in which metals are rapidly deposited at electron-deficient surface traps located at the Te–Te bond at the nanocrystal surface. The reduction potential at these trap states is sufficient to reduce the cations Pt^2+^, Pd^2+^, and Au^3+^.

**Figure 7:**
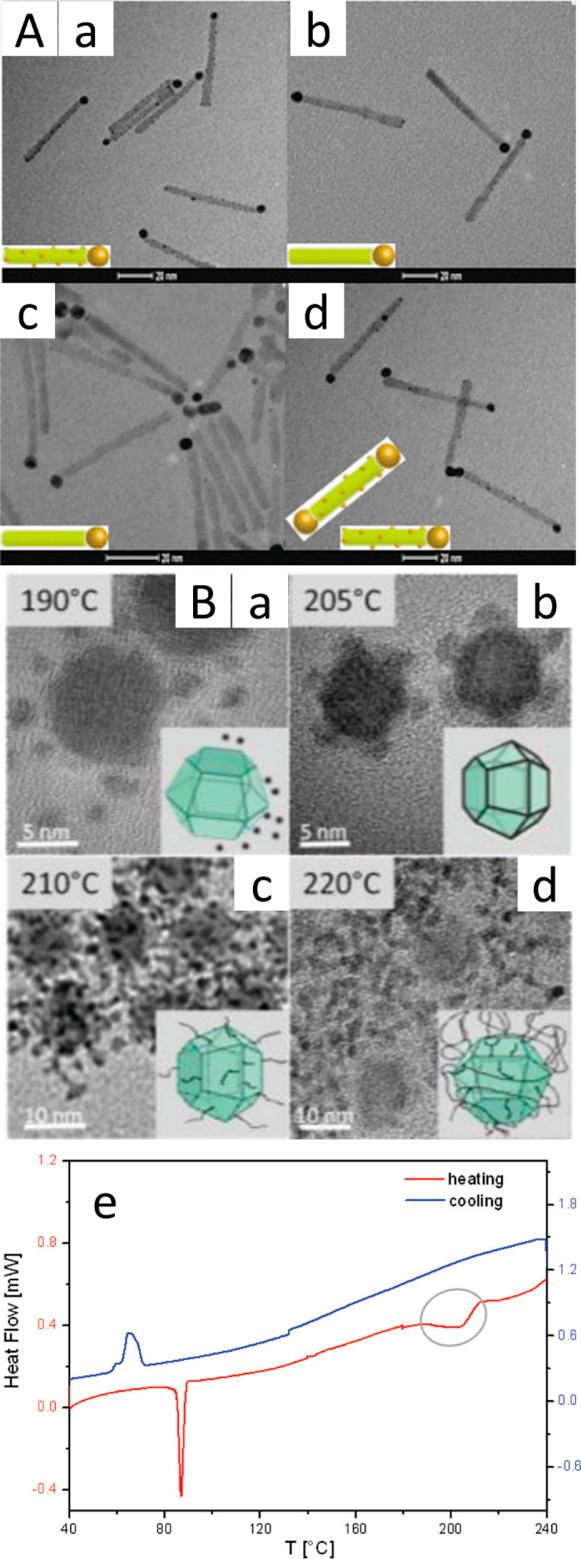
Temperature-controlled site-selective chemical metal deposition. (A) TEM images of Au growth on CdS NRs in different synthetic conditions including (a) in the dark at 313 K, (b) 473 nm laser irradiation at 313 K, (c) in the dark at 273 K, and (d) 473 nm laser irradiation at 273 K. Scale bar is 20 nm in all cases. (B) TEM images of (a) separate Ru(0) dots surrounding the Cu_2_S seeds at 190 °C, (b) Ru–Cu_2_S nanoinorganic cages formation at 205 °C, (c) surrounding Ru-strings structures at 210 °C, and (d) surrounding Ru-nets structures at 220 °C. (e) Differential scanning calorimetry (DSC) analysis on Cu_2_S seeds showing the irreversible endothermic peak at 203 °C of the heating curve highlighted by the circle. (A) Adapted with permission from ref (27). Copyright 2009 American Chemical Society. (B) Adapted with permission from ref (104). Copyright 2012 American Chemical Society.

Another strategy to arrest defect growth and surface metal decoration is through mediation via effective ligand capping and suitable temperature control.^27,100^ Single-tipped CdS–Au were achieved by lowering the reaction temperature down to 0 °C. At these conditions, phase transition of the alkyl chains of the surface amine ligands (DDA) is taking place and a static phase is formed, preventing the diffusion of Au ions to the semiconductor surfaces. Similar results were obtained by replacing the surface ligand with longer alkyl chain amines, octadecylamine, already at 25 °C, which go through phase transition at ∼32 °C^101^ (Figure 7). In addition, a postsynthesis temperature treatment was reported to suppress the multiple Au islands growth along the CdSe nanorod. Via thermal annealing, intraparticle Ostwald ripening is induced, combining both atomic and cluster diffusion.^102^ At high temperature under vacuum, the smaller Au islands migrated to the apexes of the CdSe nanorod, forming a larger single Au tip at that site. Moreover, the high-temperature treatment allowed one to overcome the energy barrier for the thermodynamically most stable configuration, resulting in a high-quality epitaxial interface between the semiconductor and the metal domains. Recently, this ripening effect of small metal islands migration to form a single large metal domain was achieved by the Langmuir–Blodgett process in which the air/diethylene glycol interface facilitated ligand exchange and ripening along the rod surface.^103^

A phase transition in the behavior of the capping ligand at different temperatures was also demonstrated to affect the selective deposition of Ru and Rh over Cu_2_S seeds.^57,58,104^ At a distinct temperature of 205 °C, metal growth along the defined edges of the faceted nanocrystals was achieved (Figure 7B-e). At this temperature, a change on the seed surface occurs, as was verified by differential scanning calorimetry analysis, which increased the surface reactivity toward heterogeneous metal nucleation. Metal deposition at lower or higher temperatures (190 and 210–220 °C) resulted in homogeneous nucleation or metal netlike structures, respectively (Figure 7).

**Figure 8:**
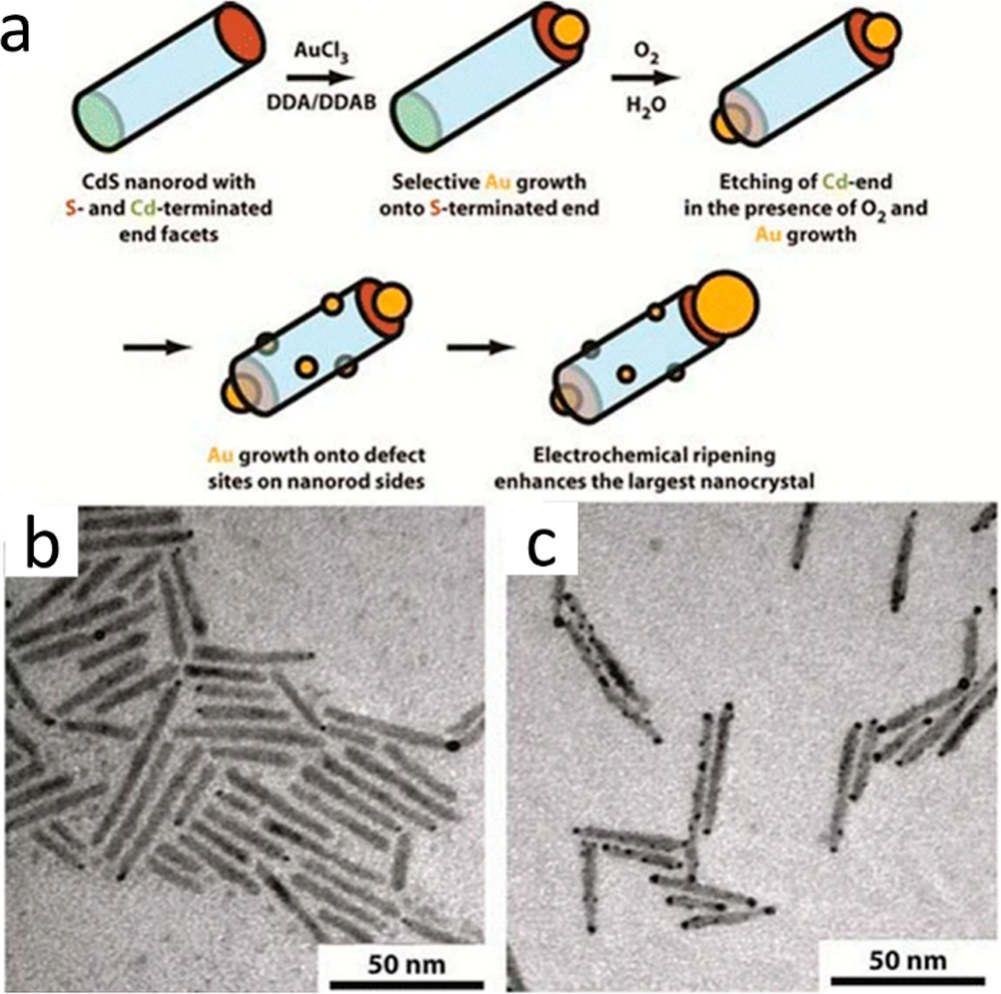
Atmospheric controlled site-selective metal deposition. (a) Schematic of the growth process of Au nanocrystals onto CdS NRs. Without oxygen, gold grows only at one tip. In the presence of oxygen, the Cd-terminated end is etched, providing a second high-energy site for gold growth, followed by side growth. TEM images of gold growth (b) under argon and (c) under air after 3 h. Adapted with permission from ref (26). Copyright 2006 American Chemical Society.

Chemical reaction conditions such as anaerobic or aerobic environments have been used to manipulate the selective deposition of metals on semiconductor components in the case of CdS–Au NRs. While an inert atmosphere led to the growth of single-metal domains on the apexes of the rod structure, in the presence of air and humidity, dumbbell-like and multiple-defect-site Au growth was obtained.^26^ When the metal reduction was performed under air, in the presence of dissolved oxygen, or with trace amounts of water in the solvent, the etching rate is enhanced or activated, contributing to an increase in defect site formation which in turn promotes Au ions reduction on it (Figure 8).

**Figure 9:**
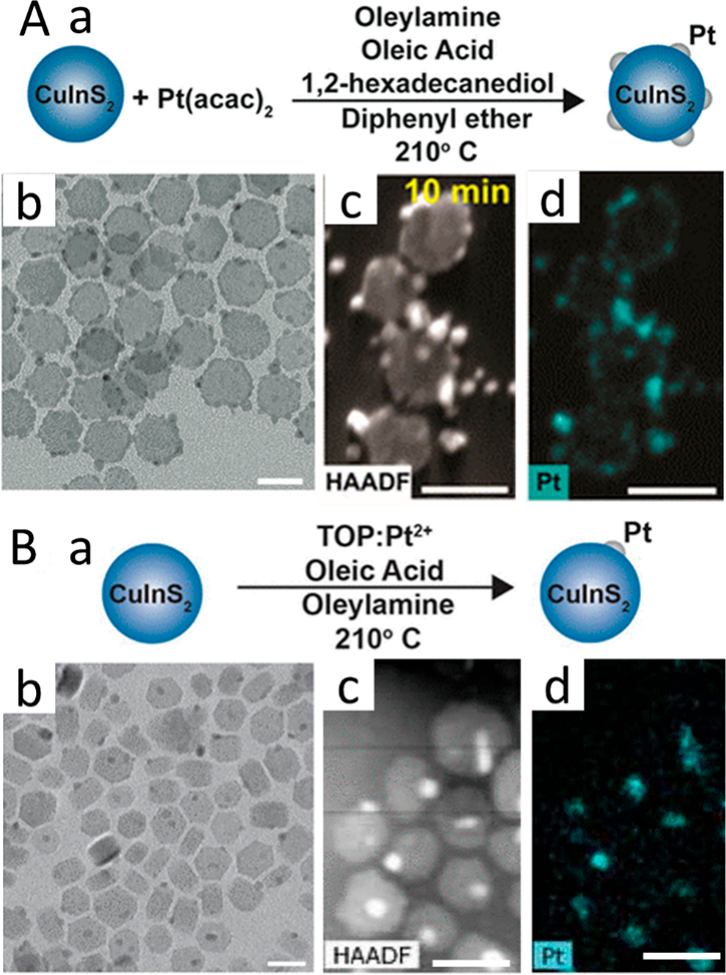
Metal precursor controlled site-selective metal deposition. (A) (a) Schematic of the growth process of Pt nanocrystals onto CuInS_2_ NPs in the presence of Pt(acac)_2_/1,2-hexadecanediol. (b) TEM images and (c, d) HAADF images and corresponding EDS mapping of CuInS_2_–Pt HNPs synthesized with 1,2-hexadecanediol. (B) (a) Schematic of the growth process of Pt nanocrystals onto CuInS_2_ NPs in the presence of complex TOP/Pt^2+^ as the precursor. (b) TEM images and (c, d) HAADF images and corresponding EDS mapping for of CuInS_2_–Pt HNPs synthesized with the complex TOP/Pt^2+^ as the precursor. Adapted with permission from ref (47). Copyright 2015 American Chemical Society.

Depending on the reaction conditions, such as different surfactants, the metal domains can be selectively deposited. Single or multiple Pt domains were selectively grown on CuInS_2_ nanoparticles by using TOP or acetylacetonate as the metal coordinating ligand, respectively (Figure 9).^47^ On the basis of hard and soft Lewis acid and base theory, in the presence of TOP, which is a soft Lewis base, its Pt binding is seemingly stronger than the hard Lewis base, acetylacetonate. Consequently, this attenuated the Pt reactivity and allowed for single-domain growth. Moreover, further limitation of the Pt reactivity was achieved by removing the additional reducing agent, 1,2-hexadecanediol, which was present in the multiple-metal island growth (Figure 9B).

**Figure 10:**
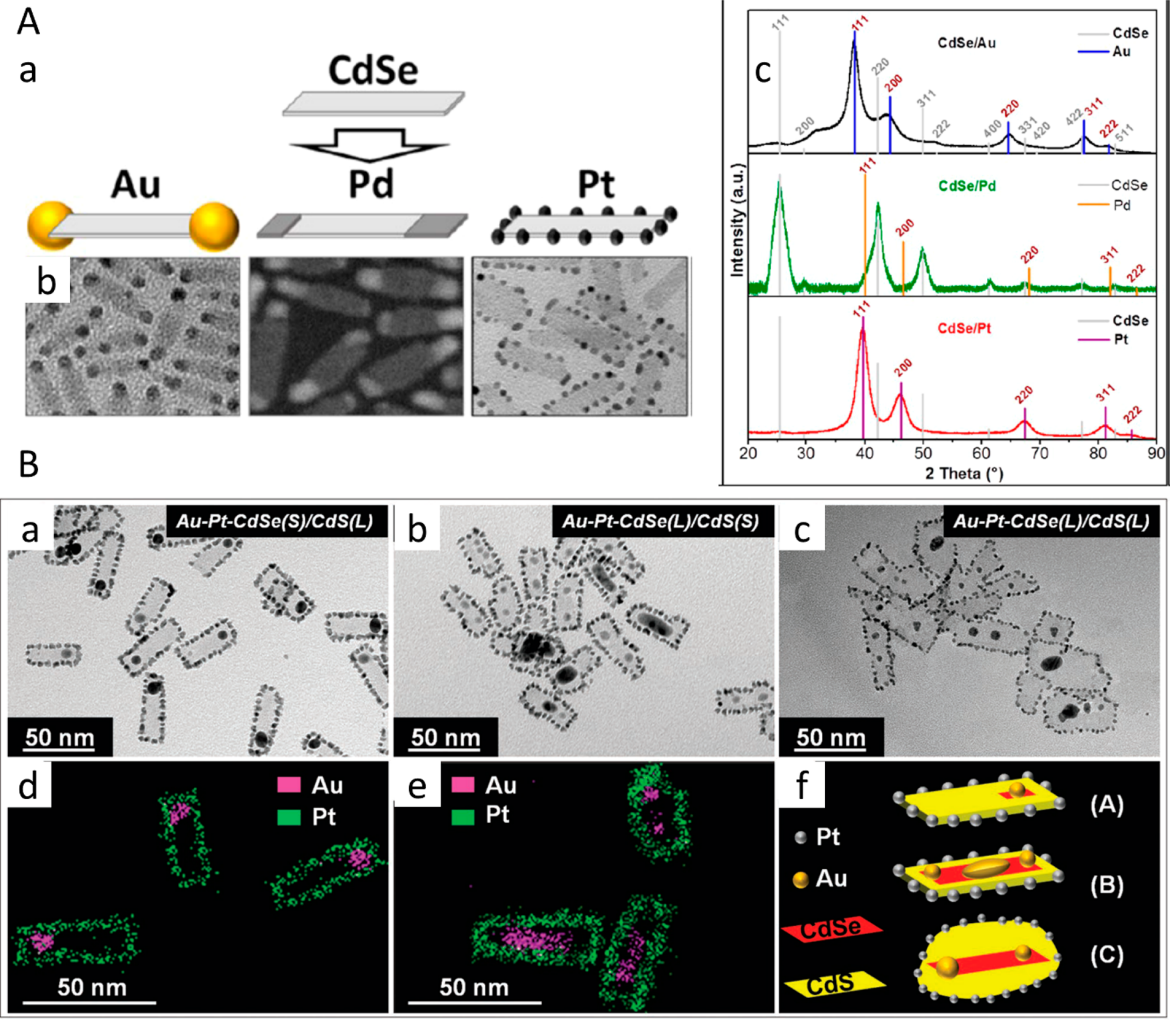
Metal-type controlled site-selective metal deposition. (A) (a) Schematic of the growth process of Au, Pd, and Pt domains on CdSe NPLs resulting in different metal decoration architecture, and (b) their complementary TEM images (c) XRD diffractograms of (top) CdSe/Au, (center) CdSe/Pd, and (bottom) CdSe/Pt NPLs. Gray lines indicate the positions and relative intensities for CdSe, and colored lines indicate the positions and relative intensities of the respective noble metal in bulk according to the literature. (B) TEM images of Au domain growth on Pt-decorated NPLs with variable CdSe core and CdS crown dimensions. (a) Small CdSe cores with large CdS crowns result in a single Au domain near the core. (b, c) Large CdSe cores with small and large CdS crowns result in either several Au domains at the CdSe/CdS interface or one single large domain near the CdSe core. (d, e) STEM-EDXS mapping of the Au growth on Pt-decorated CdSe(S)/CdS(L) and CdSe(L)/CdS(S) NPLs. (f) Schematic drawings for the three different morphologies of the NPLs. (A) Adapted with permission from ref (41). Copyright 2015 American Chemical Society. (B) Adapted with permission from ref (43). Copyright 2017 Wiley.

In a similar manner, CdSe–Au NPLs with metal deposition in different morphologies were formed depending on the reaction surfactant and reducing agents. Small Au domains growth (<5 nm) on the corners of the nanoplatelets was observed in the presence of DDA and DDAB in toluene. However, using oleylamine in 1-octadecene revealed a quasi-spherical growth at the shorter edges of the NPLs with an Au domain size of ∼5.0 nm.^41^ The authors noted that a possible fusion of initial corner growth to form larger domains at the entire edges might occur given the close proximity of the corners. Moreover, deposition of different metals including Pt and Pd resulted in different site metal deposition. Pd in a quasi-rectangular morphology grew in plane on the NPLs shorter edges, while Pt domains were deposited at all edges around the NPLs where maximum crystal defects are present (Figure 10A). It is assumed that in comparison to Au coalescence, Pt has lower mobility along the edges of the NPLs so that growth takes place at the nucleation sites only. Similar morphology control by the choice of the reducing agents was demonstrated by Manna and co-workers, synthesizing either CuS–Au core–shell or Janus hybrid NPLs via Au ion reduction in the presence of ascorbic acid or oleylamine/ascorbic acid surfactants, respectively.^105^

Differences in the growth of noble metal domains may also be attributed to the different reactivities of the metal precursors present in the respective systems. In a sequential work by the same group, quaternary Au- and Pt-decorated CdSe/CdS core/crown NPLs were presented with site-selective metal deposition.^43^ In all cases, independent of the CdSe core and CdS crown size, Pt domains are found only at the edges of the NPLs (Figure 10B). In this case, deposition of Au, was observed in different sites depending on the CdSe core and CdS crown dimensions. Small quasi-spherical Au domains surrounding the edges was seen on small CdSe core NPLs (Figure 10B-a,d) and single or multiple larger Au islands (4–6.5 nm) on the surface of large CdSe core with small and large CdS crown NPLs (Figure 10B-b,c,e).

**Figure 11:**
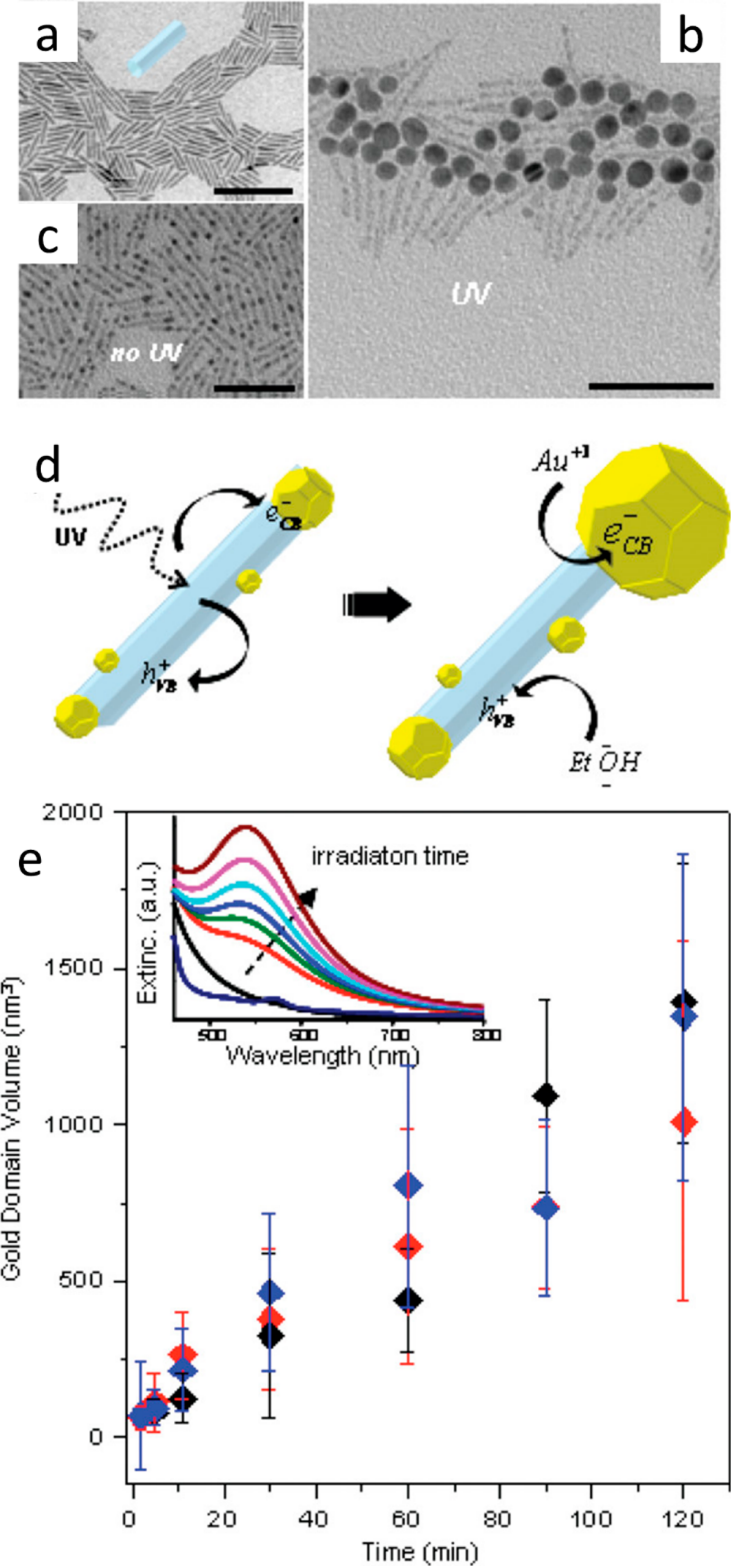
Light-controlled site-selective metal deposition. TEM images of (a) pristine CdS NRs, (b) resulting CdS–Au NRs following UV irradiation, and (c) Au growth without UV light (all scale bars 50 nm). (d) Schematic of photoinduced growth mechanism of large gold domains on semiconductor CdS NRs. (e) Gold domain volume increases over growth time duration. Red, CdSe/CdS nanorods; blue, CdS nanorods; black, nanodumbbells. (Inset) Optical extinction with increasing UV irradiation time. Plasmon peak at 536 nm grows over time. Adapted with permission from ref (65). Copyright 2009 American Chemical Society.

Light-induced reduction is another pathway for tuning and selectively depositing metals on semiconductor components. A complementary strategy to thermal annealing and Ostwald ripening (described above) in forming one-side Au-tipped CdS or CdSe/CdS NRs was presented by Sönnichsen and co-workers and others^27,106^ via UV light excitation of CdS or CdSe/CdS semiconductor nanorods in Au precursors and surfactant solution. In this method, the ripening mechanism was suppressed, and instead, light-induced metal growth on preillumination metal nuclei was suggested (Figure 11).^65^ Following spontaneous growth of small metal domains favorably on the rod apexes, upon UV excitation, the excited electrons created in the semiconductor migrate preferentially to one metal tip, reducing further Au^3+^ ions on the seed and resulting in a single large metal domain at the expense of the initially formed sites. Metal growth was monitored via absorption measurements, allowing the calculation of metal domain volume along the reaction time (Figure 11e). To neutralize the system, excited holes are scavenged by sacrificial hole scavengers in the solution such as ethanol. In a similar manner but a less controlled deposition, CZTS–Au nanorods were obtained.^36^ While thermal metal growth formed a surface decoration of multiple small metal particles (∼2 nm), applying light-assisted metal deposition yielded two large metal sites (∼15 nm) per rod in average, yet, surface decoration by small metal domains was not entirely arrested.

**Figure 12:**
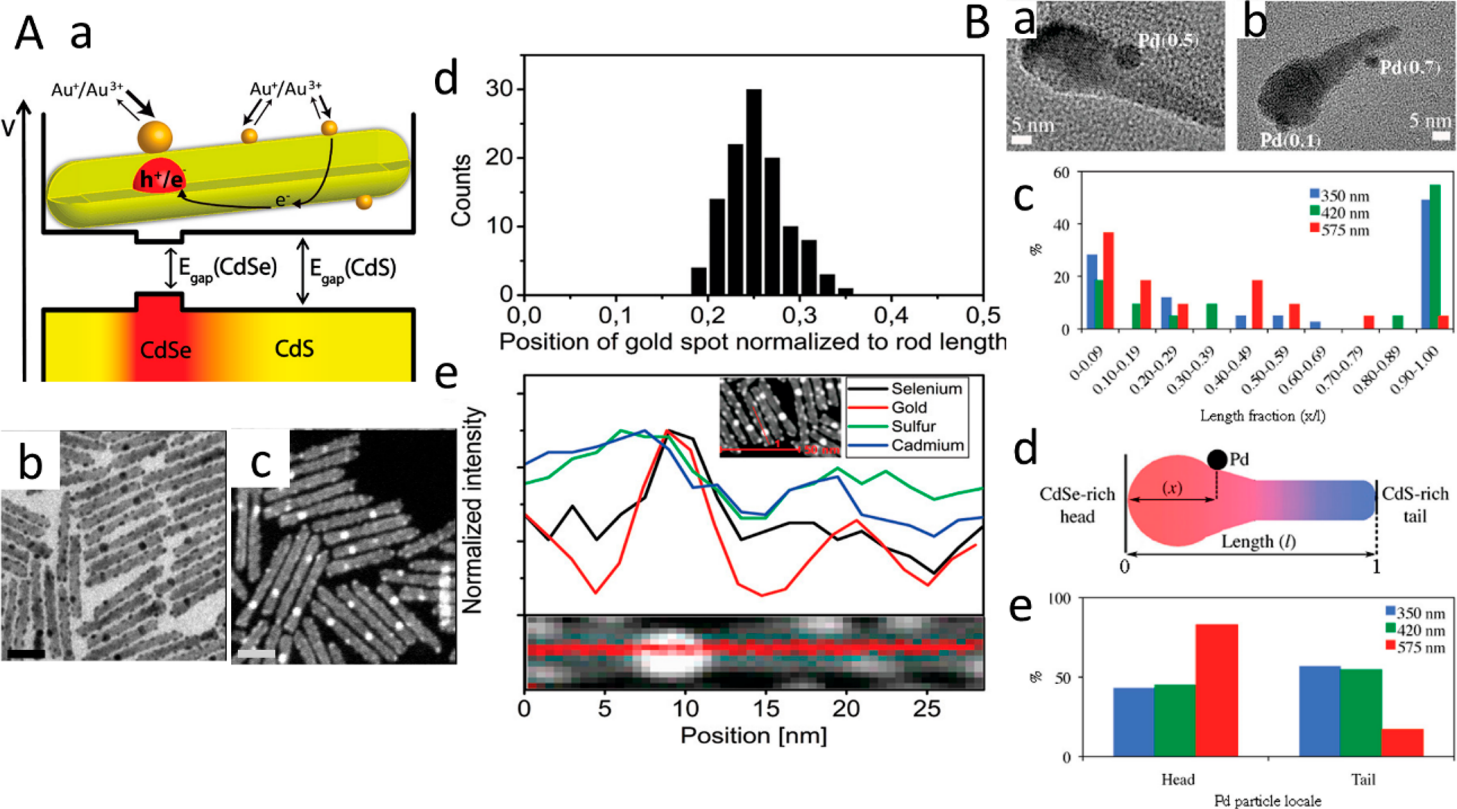
Semiconductor structure controlled light-induced site-selective deposition. (A) (a) Scheme and potential diagram for the CdSe/CdS NRs along with a schematic of the electrochemical ripening mechanism for gold growth. (b) TEM and (c) HAADF-STEM images of CdSe/CdS–Au hybrid NRs. (d) Histogram of the seed position relative to the length of the rod. (e) Elemental analysis (EDS line scan, 1.5 nm step size) of a single nanorod with a big gold dot. (B) (a, b) TEM images of light-induced deposition of Pd on axially anisotropic CdS_0.4_Se_0.6_ NRs with metal sites length fractions. (c) Location of Pd metal site along the length of the NR, plotted as a fraction of the total length of the nanorods. (d) Schematic of CdS_0.4_Se_0.6_–Pd hybrid NRs, and length fraction measurements. (e) Head-side vs tail-side population of Pd nanoparticles on CdS_0.4_Se_0.6_ NRs. (A) Adapted with permission from ref (64). Copyright 2008 American Chemical Society. (B) Adapted with permission from ref (108). Copyright 2011 American Chemical Society.

Photoreduction deposition was also utilized to selectively deposit metal domains on specific sites based on the semiconductor heterostructure composition. Comparing Pt light-induced deposition on CdS nanorods and CdSe/CdS-seeded nanorods revealed different metal decoration.^63^ While Pt growth on CdS NRs showed heterogeneous growth on their surface with up to 6 islands per rod, mostly a single-metal domain was observed following photodeposition of Pt on CdSe/CdS NRs. Moreover, the location of the Pt island is near the CdSe seed. This specific site deposition is ascribed to the localization of the formed excitons under illumination. This trend was also observed for CdSe/CdS–Au NRs where a large Au domain is deposited at the CdSe seed region under irradiation conditions (Figure 12A).^64^ Similarly, The preference of this site was attributed to the electronic profile of the heterostructure in which excited electrons and a hole are relaxing at the CdSe seed location and allows Au ion reduction at this site (Figure 12A-a). The effect of lattice mismatch and therefore enhanced surface defects at the seed region was considered to be less dominant since ZnSe-seeded CdS nanorods do not show any preferential growth location, even though the seed region is shown to be more defective than in the case of CdSe-seeded rods.^107^ Moreover, less effective selective deposition was observed for a thicker CdS shell around the CdSe seed due to a larger potential barrier, which further supports the suggested mechanism.

Further extension of this selective deposition methodology was demonstrated in photodeposition of Pd nanoparticles on CdS_0.4_Se_0.6_ nanorods which have CdSe- and CdS-rich domains on opposite apexes of the rod. A dependence on irradiation wavelength was observed, allowing for preferred growth at the CdSe-rich end upon irradiation in the red or on both CdSe-rich and CdS-rich regions upon blue irradiation (Figure 12B).^108^ This partial site selectivity was explained by the varying band gaps and absorption properties of the two different semiconductor regions (CdSe has a smaller gap than CdS) accompanied by trapping of the electrons on surface defects in that area.

The charge transfer of an excited charge carrier to already deposited metal domains upon light stimulation can be also exploited for selective deposition of additional different metal types on top of the former metal decoration as will be described in detail in section 3.2.

#### 2.3.2 Morphology: Size and Shape Control

Another aspect of structural control of the HNPs synthesis is tuning and tailoring the morphology of HNP structures along with their component’s sizes. The size and shape of both the semiconductor and the metal components can affect the physical and chemical properties of such hybrid nanosystems. Early investigation of semiconductor–metal HNPs showed fair control of the hybrids morphology via temperature-dependence syntheses. CdS–FePt have been synthesized in core/shell and heterodimer structures depending on the reaction temperature. While Cd ions reduced on FePt-sulfidized nanoparticles at 100 °C formed a core/shell structure, raising the solution temperature to 280 °C converts CdS from its amorphous to a crystalline state, accompanied by a dewetting process, and results in heterodimers of FePt–CdS HNPs.^77^ This method was also demonstrated for the growth of CdS, ZnS, PbS, and CdSe on the FePt nanoparticle’s surface, achieving improved stability and control by increased surfactant concentration in the reaction and gradually raising the reaction temperature in a 5 °C/min rate.^79^ FePt–CdS/Se sponge-like nanostructures were obtained by first introducing chalcogen (sulfur or selenium) precursors, promoting the formation of nanowires (NWs) of chalcogens which serve to connect FePt nanoparticles.^78^ Successive addition of Cd(acac)_2_ allowed formation of the hybrid nanosponges.

**Figure 13:**
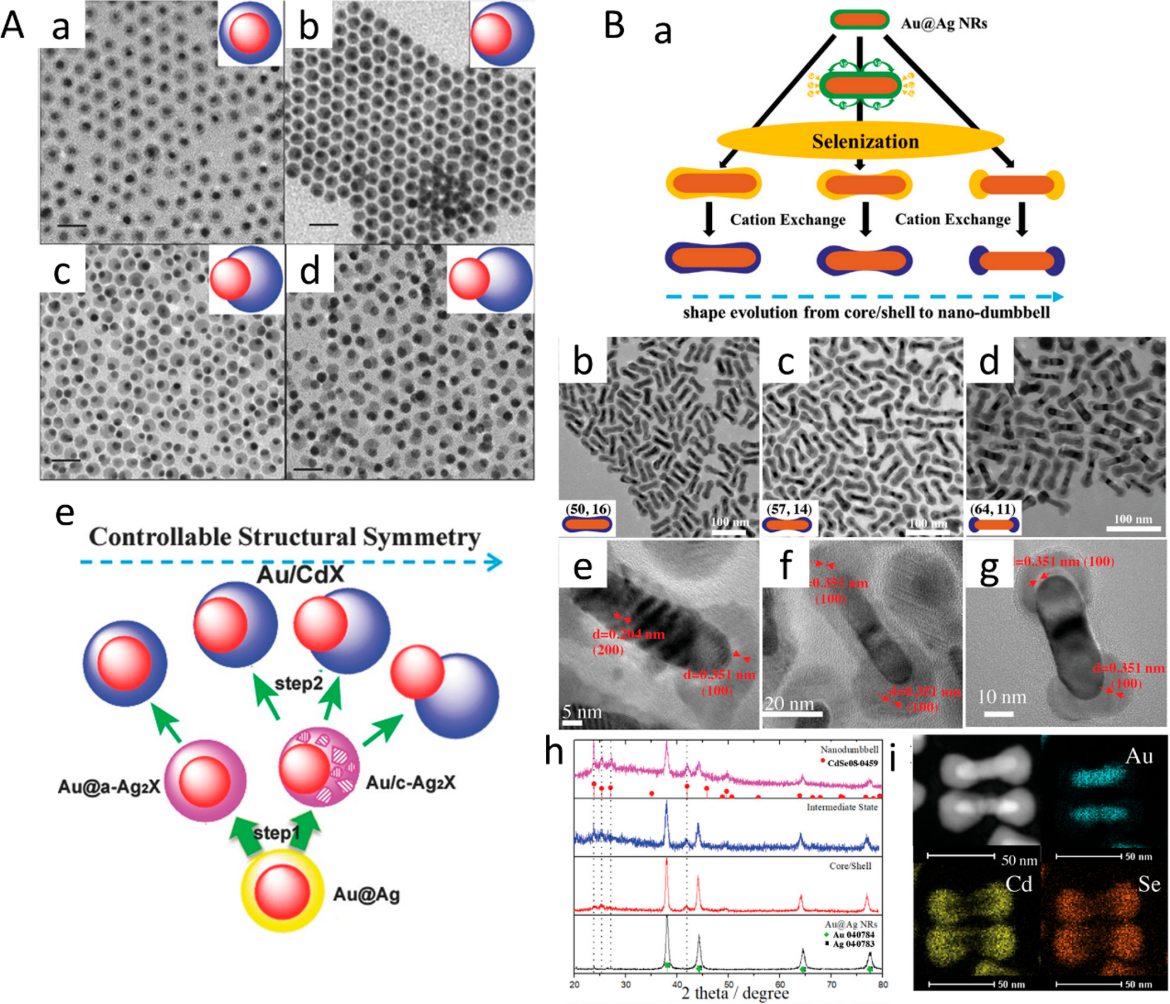
Morphology control. (A) TEM images of (a) concentric core/shell, (b) nonconcentric core/shell, and (c, d) Janus HNPs. (Insets) Phase separation-induced Au/CdS morphologies. Scale bar: 20 nm. (e) Schematic of the synthetic process for controllable structural symmetries in Au/CdX (X = S, Se, and Te) hybrid structures with large lattice mismatch by two steps of in situ chemical conversion: a-Ag_2_X, amorphous Ag_2_X; c-Ag_2_X, crystalline Ag_2_X. (B) (a) Schematic of controlled structural symmetries synthesis of Au/CdSe HNPs with large lattice mismatch by two steps of in situ chemical conversion. TEM and HRTEM of (b, e) Au/CdSe core/shell, (c, f) Au/CdSe intermediate state, and (d, g) Au/CdSe nanodumbbells. (h) XRD pattern of the as-prepared Au/Ag NRs, Au/CdSe core/shell, Au/CdSe intermediate state, and Au/CdSe nanodumbbells. (i) EDX elemental mapping results of Au, Cd, and Se for the Au/CdSe nanodumbbell. (A) Adapted with permission from ref (85). Copyright 2013 Wiley. (B) Adapted with permission from ref (109). Copyright 2019 Wiley.

Further control of the anisotropic nature of Janus HNPs was gained by taking advantage of chemical thermodynamics-directed colloidal strain. Au–Ag core/shell nanoparticles were used as seeds for initial sulfidation toward Au–Ag_2_X (X = S, Se, Te). The degree of Ag_2_X crystallinity dictated the extent of component separation following cation exchange to form a family of gradually separated Janus HNPs including Au–CdS, Au–CdSe, and Au–CdTe (Figure 13A-a).^85^ In general, the higher crystallization of the Ag_2_X shell as well as the higher reaction temperature of the cation exchange reaction lead to larger phase separation between Au and CdX, reducing the interfacial and grain boundary energies (Figure 13A-b). The controlled separation of Ag_2_X from Au seeds was also reported for Au/Ag NRs where selenization by different Se concentrations allowed the formation of Au–CdSe core/shell NRs and CdSe-tipped Au nanorod structures (Figure 13B).^109^

**Figure 14:**
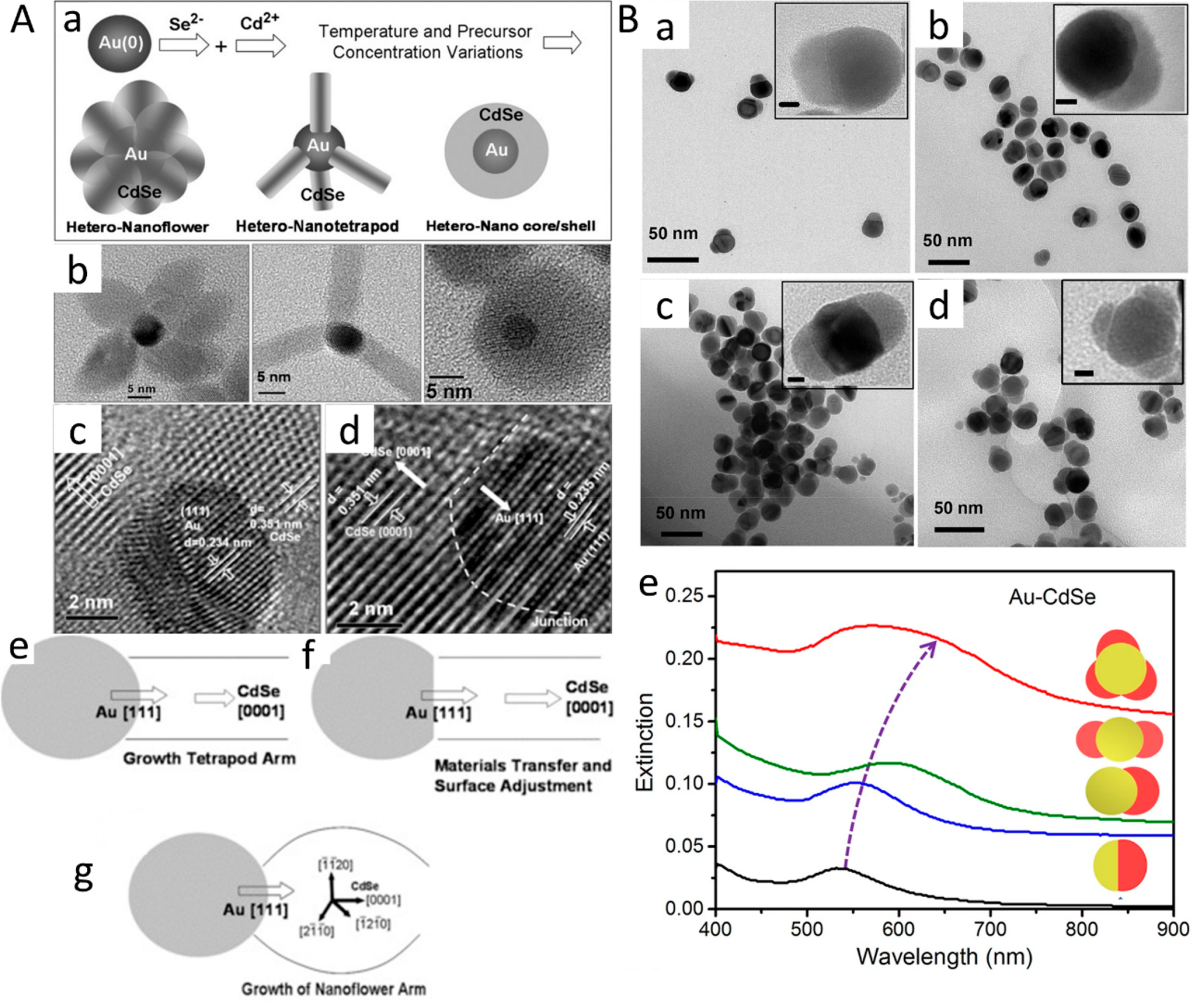
Morphology control via precursor concentrations and pH. (A) (a) Schematic of formation of flower-, tetrapod-, and core/shell-shaped HNPs with an Au core and CdSe arms/shell. (b) TEM images of three different shapes of HNPs. HRTEM images of (c) tetrapod- and (d) flower-shaped HNPs showing the lattice mismatch at the heterojunctions. In both cases, 3 × d (111) of Au remains equal to 2 × d (0001) of CdSe. Schematic of the synthesis mechanism of (e, f) tetrapod- and (g) flower-shaped HNPs. (B) TEM images of Au/CdSe HNPs including (a) Janus nanospheres, (b) heterodimers, (c) symmetric double-domain NPs, and (d) multidomain NPs (scale bars in the insets are 5 nm). (e) UV–vis–NiR spectra of Au/CdSe HNPs with different morphologies such as Janus nanospheres (pH = 2.5), heterodimers (pH = 4.5), symmetric double-headed nanoparticles (pH = 7.2), and multiheaded nanoparticles (pH = 8.1). (A) Adapted with permission from ref (110). Copyright 2013 Wiley. (B) Adapted with permission from ref (113). Copyright 2019 Springer Nature.

Patra et al. showed morphology control of Au–CdSe HNPs by varying the precursor concentrations and reaction temperatures. CdSe growth on the Au nanoparticle surface in shapes of nanoflowers, tetrapods, and core/shell was achieved (Figure 14A). While the same Se concentrations were used in the nanoflower and tetrapod structures, the lower reaction temperature in the flower-shaped synthesis decreased the growth rate along the [0001] direction of the wurtzite crystal and allowed also growth in the ⟨2110⟩ direction as shown and illustrated in Figure 14A-e–g.^110^ A Core/shell architecture was obtained using a lower chalcogenide precursor concentration in which insufficient monomer in the reaction system did not allow them to grow further to obtain nanoflowers.

Using a related approach, a different type of control over the HNPs morphology was demonstrated in the synthesis of Cu–Cu_*x*_S HNPs. This was achieved by sulfidation under two different environments, H_2_/argon and argon. While the former resulted in Janus formation, the latter produced predominantly hetero-oligomer HNPs. This morphology control is assigned to an outcome of an interplay between a kinetically controlled reaction in the presence of H_2_ as a reducing agent and a thermodynamically controlled reaction governed by self-diffusion of the ions.^111^

Au–Cu_2–*x*_Se HNPs with different morphologies were synthesized by adding different polymers as stabilizers during the selenization process.^112^ Recently, a different approach of morphology control was achieved by changing the pH value of Au–CdSe HNPs aqueous mediated synthesis. The synthesis procedure included Ag deposition and selenization followed by Cd(NO_3_)_2_ cation exchange at 2.5, 4.5, 7.2, and 8.1 pH values. Janus nanospheres, heterodimers, symmetric double-headed nanoparticles, and multiheaded nanoparticles were formed, respectively (Figure 14B). At higher pH values, both Ag deposition on the Au surface in the first step and Cd ion exchange in the second step are accelerated, promoting a thicker Ag shell and stronger Ag_2_S ripening, respectively, both contributing to additional selective growth sites.^113^ In addition, the increased growth of the CdSe sites induces a plasmon energy band red shift due to an increase in the effective refractive index of the environment (Figure 14B-e).

**Figure 15:**
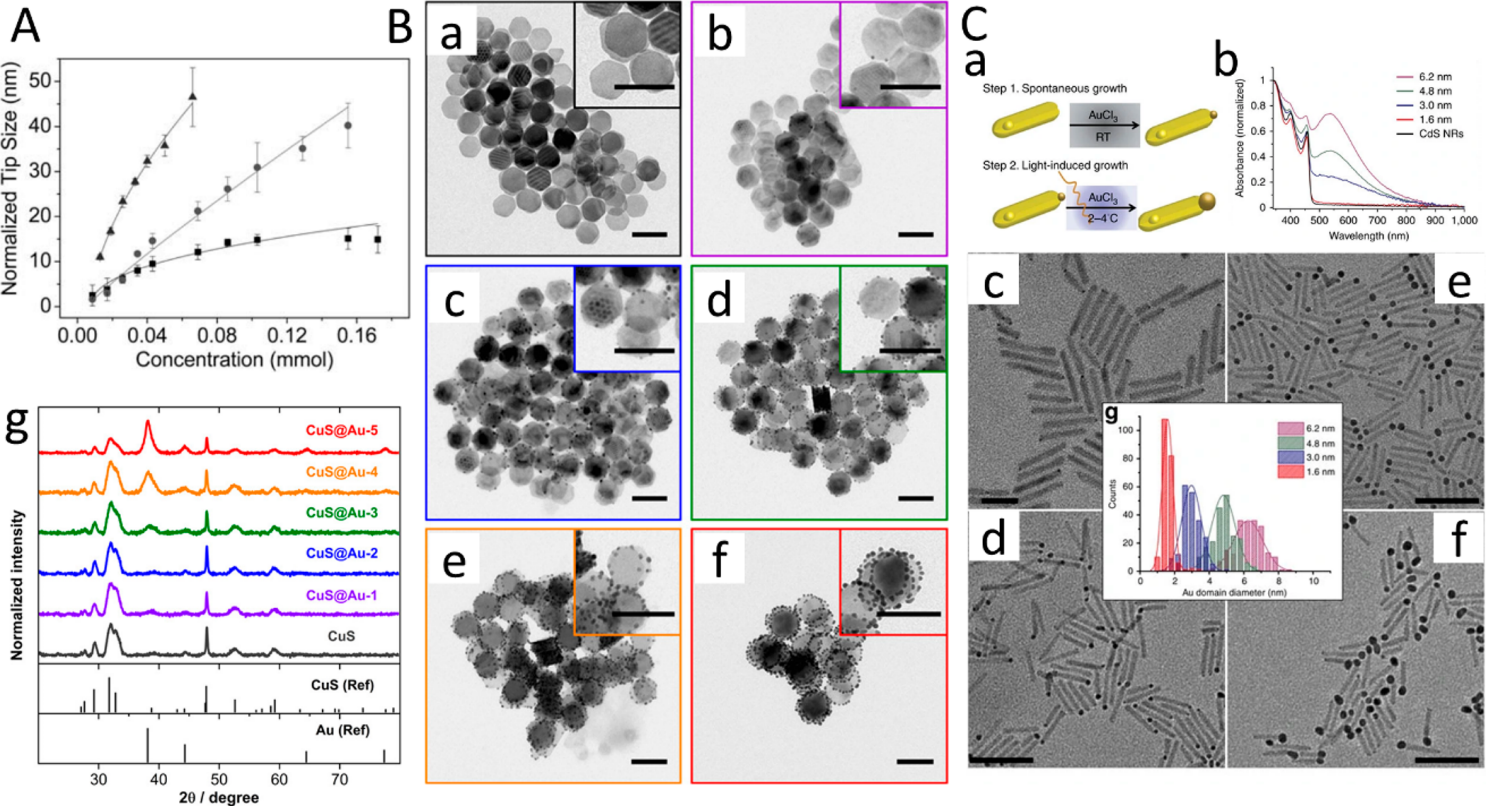
Metal domain size control. (A) Normalized metal tip size vs concentration of Au precursor for CdS (square), CdSe (circle), and CdTe (triangle) NRs. (B) TEM images and corresponding close-up images (inset) of (a) CuS NPLs and (b–f) CuS–Au hybrid NPLs synthesized with different amounts of Au^3+^ ions (scale bars 100 nm). (g) XRD patterns of CuS and CuS–Au HNPs. Standard XRD patterns of CuS (covellite, ICDD pattern no. 06-0464) and Au (ICDD pattern no. 04-0784) were used as references. Each diffractogram was normalized to the maximum intensity of the respective pattern. (C) (a) Schematic of the two-step metal growth deposition. (b) UV–vis absorbance spectra of CdS–Au hybrid NRs showing the development of the plasmonic feature around 540 nm as the Au tip size increases. TEM images of CdS–Au hybrid nanoparticles (c) with a 1.5 ± 0.2 nm Au tip size after 1 h dark synthesis and light-induced synthesis for 30 min with various CdS:Au molar ratios, leading to Au tip sizes of (d) 3.0 ± 0.5, (e) 4.8 ± 0.7, and (f) 6.2 ± 0.8 nm. (g) Size distribution histogram of the Au metal tip diameters. (A) Adapted with permission from ref (106). Copyright 2010 Royal Society of Chemistry. (B) Adapted with permission from ref (53). Copyright 2022 American Chemical Society. (C) Adapted with permission from ref (19). Copyright 2016 Nature Publishing Group.

As the size and shape of the semiconductor part can be synthetically controlled, HNPs with different sizes of metal components can be obtained across extended regimes via diverse synthetic approaches. Deposition of small cluster size domain metal particles (up to ∼200 atoms) was achieved using laser ablation methods.^114^ Larger sizes, from several to a few tens of nanometers, were achieved via controlling the reaction temperature and/or precursor concentration. The latter approach was demonstrated on different hybrid nanosystems in several studies including CdX–Au nanorods (X = S, Se, Te) where metal tip sizes reaching up to 40 nm in fast reactions times (<120 s) were achieved (Figure 15A).^115^ Similar control was reported for Au growth over the edges of covellite copper sulfide NPLs. Increased metal concentration and reaction times formed larger sized domains and a higher number of metal domains per particle as well (Figure 15B).^62^ Size control of Pt and Ni metal domains was demonstrated for CdS NRs,^116^ CdSe, and CdSe/CdS NRs^117^ and NPLs,^41,43^ depositing Pt nanoparticles from 0.7 to 3.5 nm and Ni domain in the range from 2.3 to 10.1 nm. Thermal control was also suggested to allow tuning of the metal tip size. By using oleylamine solution and gold–oleate complexes as precursors at a temperature range between 95 and 140 °C,^118^ the Au tip sizes ranged between 3 and 16 nm, whereas higher temperatures yielded larger metal tips in the hybrid nanoparticles.

An additional degree of control was achieved by consecutive two-step synthesis combining spontaneous chemical reduction and light-induced reduction deposition. Specifically, following spontaneous metal nucleation in dark conditions to form site-selective small metal islands on the apexes of the CdS NRs, light-induced metal deposition at low temperature (2–4 °C) allowed the metal domain size to be controlled by varying the Au ions/NRs molar ratio (Figure 15C).^28^

With increased metal domain sizes, stability and aggregation phenomena have been reported especially in organic media. A novel synthesis in an aqueous high dielectric environment was reported to achieve high stability and size control of Au metal tips on CdSe/CdS NRs.^70^ Au metal domains close to 50 nm in size were obtained by photochemically reducing Au ions on already phase-transferred NRs in ethylene glycol/H_2_O solvent with polyvinylpyrrolidone (PVP) as the reducing agent. The choice of PVP also allowed further surface functionalization of the gold domain since it is only loosely covered by PVP surfactants.

In addition, control of the metal domain shape was achieved by selective deposition of faceted Pt metal tips on CdS nanorods. By successive metal deposition reactions, nucleation of nonfaceted Pt tips on CdS nanorods was followed by facet-selective Pt deposition in the presence of CO molecules that favor the (100) facet growth over the (111) facet due to stronger binding to the former.^119^ This strategy resulted in cube-shaped Pt tips with well-defined (100) facets.

Control over the morphology of HNPs can also dictate their assembly in more complex formations such as colloidal networks,^120^ gels,^38^ and aerogels.^106,121,122^ Recently, Bigall and co-workers showed the influence of metal decoration architecture on the structural properties of HNPs gels and aerogels. While random Au metal decoration on the surface of CdS NRs led to a network which was mainly connected between the NRs (rod to rod connections), Au-tipped CdS NRs result in gold only in the connection points between the NRs.^106^ Similar structural effects were reported for CdS NPLs decorated with Pt NPs.^121^ Hybrid NPL network gelation reveals a homogeneous distribution of the metal in the cryoaerogel, in contrast to accumulation of Pt–NPs in close vicinity in the case of mixing and cogelation of NPLs and Pt NPs. Such differences in the metal distributions are expected to have a significant impact on the optical and chemical properties of HNP-based aerogels.

#### 2.3.3 Material Composition Control

As described above, hybridization of the two different materials, semiconductor and metal, provided new synergistic properties. In addition, combination of more than one type of semiconductor or metal component can expand the chemical and physical characteristics of such HNPs. The influence of such combinations was previously presented in bimetallic nanoparticle systems, where changes in a variety of properties were observed including electric field enhancement effects, modification of surface plasmon properties, magnetic functionality, and, most notably, enhanced catalytic activity.^123,124^ Throughout the previous sections some examples of bimetallic–semiconductor nanoparticles were reviewed. Such nanosystems with different architectures including FePt–Cd–chalcogenide core/shell and heterodimers^77−79^ along with AuCu–CdS star-like nanostructures^125^ or spherical CdSe nanoparticles decorated with an alloy of AuPd^126^ islands were synthesized via solvothermal reactions. Either presynthesis of binary/alloyed metal nanoparticles was used as in the former examples or chemical reduction in the presence of both metal precursors on the semiconductor surface or apexes was used as demonstrated for CdS NRs tipped with PtNi and PtCo alloys.^44^ A different mechanism of exploiting the favorable heterogeneous growth on a pre-existing metal domain serving as a substrate, over homogeneous nucleation of free-standing metal nanoparticles in solution, was reported by Pyun et al. Activation of small Pt islands at the apexes of such NRs allowed the formation of a Co shell or a hollow cobalt oxide shell via a Kirkendall effect in the presence of O_2_ under high temperature.^55^

**Figure 16:**
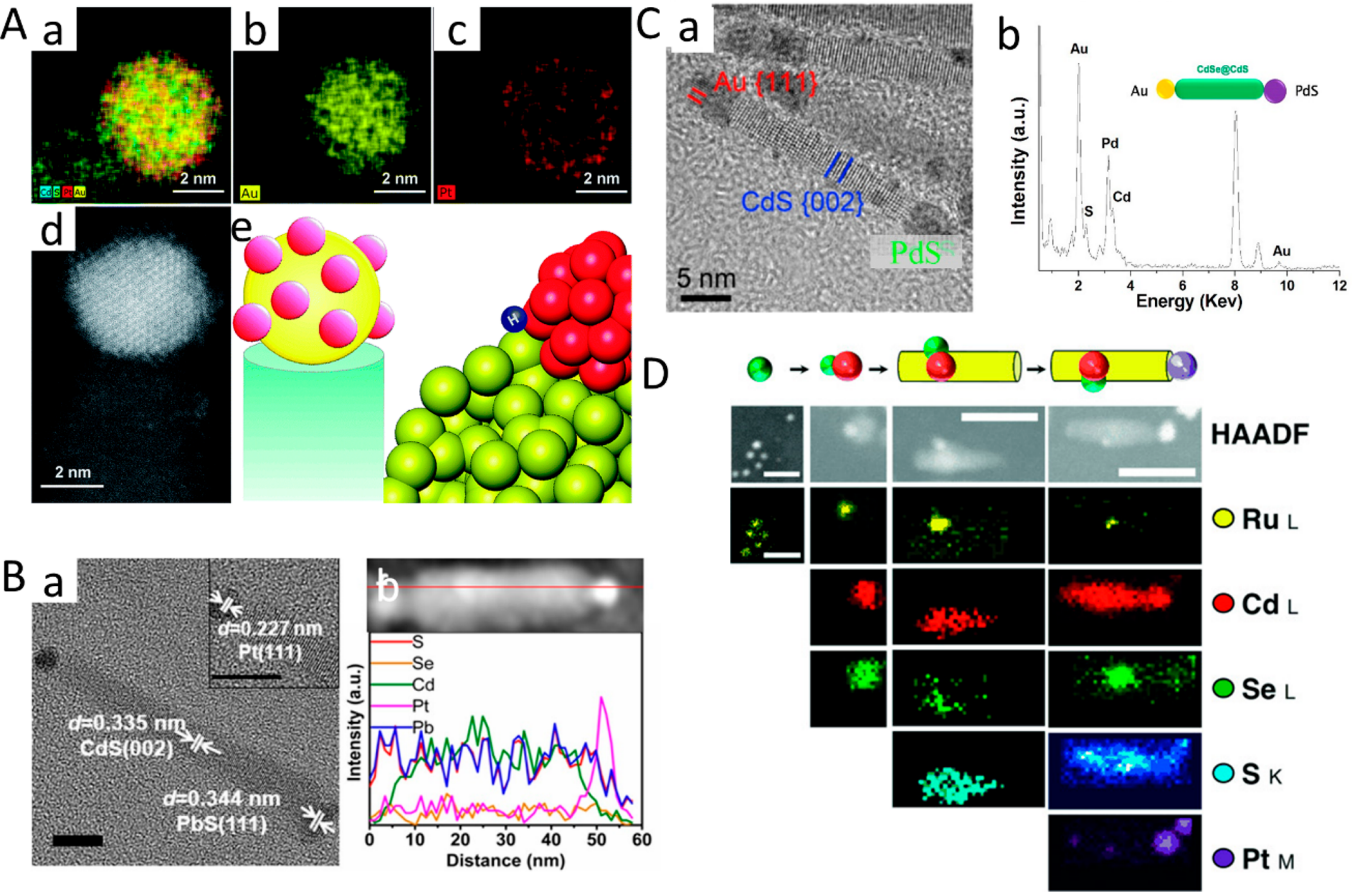
Material composition control. (A) (a–c) EDS mapping and (d) atomic resolution HAADF image of the bimetallic tip. (e) Schematic demonstrating of a Au core that is decorated with islands of Pt. (B) (a) HRTEM and (b) HAADF images with (c) corresponding EDS elemental profiles of PbS–CdSe@CdS–Pt HNPs. (Inset in a) High-magnification image of the rod near the Pt tip. All scale bars are 10 nm. Elemental analysis includes profiles of the S-K (red), Se-L (orange), Cd-L (green), Pt-M (pink), and Pb-M (blue) lines. Red line on the HAADF image represents the line scan where the EDS profile was recorded. (C) (a) HRTEM image and (b) EDS spectra of PdS–CdSe@CdS–Au hybrid NRs. (D) HAADF and elemental analysis of the different components within Ru–CdSe/CdS–Pt hybrid NRs (scale bar 10 nm for Ru and 20 nm for the rods). (A) Adapted with permission from ref (68). Copyright 2015 Royal Society of Chemistry. (B) Adapted with permission from ref (130). Copyright 2016 American Chemical Society. (C) Adapted with permission from ref (131). Copyright 2018 Elsevier. (D) Adapted with permission from ref (133). Copyright 2015 Wiley.

This kind of synthetic control, similarly to the other forms of structural control, can be achieved by a light-induced route as well. Selective deposition of Pd on Au-tipped CdSe/CdS NRs was conducted under UV illumination. Exploiting the formed excitons to provide reductive charges at a specific site, the preexisting Au seed, due to charge transfer from the semiconductor segment to the metal domain, led to the reduction of Pd ions and formation of an alloyed AuPd tip which revealed interesting magnetic properties.^66^ This strategy was adopted and utilized for the formation of several bimetal tip deposition in different hybrid nanosystems. Au-tipped CdSe/CdS NRs were used as template for photochemical reduction of other noble metals such as Pt^68^ and Pd.^67^ Controlling the order of metal precursors addition and depending on their reduction potential (Au possesses a higher reduction potential than Pt), different morphologies of the binary metal tip could be achieved, from core/shell through core and alloyed shell and formation of a single-metal core with small secondary metal islands deposition (Figure 16A). Similarly, such morphology control was shown by Bar-Sadan and co-workers on CdSe/CdS–Ag/Pd NRs, where an Ag–Pd core/shell structure was obtained along with alloyed tips (Figure 4B).^127^ The tendency for Ag cation exchange to form Ag_2_S was avoided by slow addition of the reactants and an overall low loading (∼5% of the existing Cd atoms in the rods). In addition, the use of illumination for the photodeposition of the metals facilitates the reduction of Ag^+^ to Ag^0^. Other hybrid nanosystems syntheses were also reported to employ photochemical deposition including NiCo-decorated Zn_0.5_Cd_0.5_S nanoparticles^128^ and PtPd on CdS nanoparticles^129^ utilizing these structures for photocatalytic applications.

Along with the deposition of binary metal domains, disparate metals and metal chalcogenide types on different sites were also synthetically obtained. Mokari et al. exploited the nanorod architecture to synthesized PbS–CdS–Pt HNPs where the metal and metal chalcogenide domains are each deposited on the opposite apex of the rod structure (Figure 16B).^130^ By depositing first the Pt by solvothermal growth on one side due to the favored reactive facet, it serves as a blocking material, preventing further growth at this site and promoting secondary PbS growth on the opposite apex through thermal decomposition of Pb–bis(diethyldithiocarbamate). In a similar manner, asymmetrically tipped PdS–CdSe/CdS–Au NRs were obtained.^131^ Following Au deposition by common spontaneous metal growth in the presence of DDA in toluene solution, cation exchange at 180 °C was performed to achieve PdS on the other rod tip (Figure 16C). A different synthetic approach was presented by Li and co-workers to form asymmetric Cu_1.94_S–Zn_*x*_Cd_1–*x*_S–Pt HNPs.^132^ In this case, Cu_1.94_S nanospheres were initially prepared as starting materials followed by epitaxial growth of Zn_*x*_Cd_1–*x*_S. Pt decoration at the surface of the Zn_*x*_Cd_1–*x*_S component was performed via photochemical deposition in aqueous solution. Combination of separate domains of Ru and Pt on CdSe/CdS NRs was also obtained by presynthesis of CdSe–Ru hybrid dimers and their use as seeds for epitaxial growth of a CdS rod-like shell.^133^ Controlled and gradual displacement of the dimer surface ligands was found to stabilize them and minimize detachment. Lastly, Pt thermal deposition of small domains mostly at the rod tips was conducted (Figure 16D). Sequential metal deposition on CdSe NRs of Pt, again by thermal deposition and thereafter Au growth via dropwise addition of Au ions in DDAB and DDA toluene solution, resulted in nanodumbbells with heterometal tips, Pt–CdSe–Au.^134^ Note that the inverse sequence of the precursor addition did not form the asymmetrical architecture because an irregular deposition of Au occurred at the CdSe nanorods. These two metal types were also deposited separately on MoS_2_–CdS nanostructures (CdS nanospheres on MoS_2_ sheets).^135^ Au loading was formed through reducing HAuCl_4_ by sodium citrate followed by photochemical deposition of Pt, which allowed control of their size and density.

Another example of synthetic control via sequential metal deposition was demonstrated by binary metal deposition of Au and Pt on CdSe/CdS core/crown NPLs.^43^ Au growth, followed by Pt deposition, formed a Pt–Au alloy or core/shell type morphology. Surprisingly, Au deposition on already decorated Pt–CdSe/CdS NPLs resulted in a large Au metal domain mostly on the CdSe core. This observation is in contrast to previous reports on other hybrid nanosystems where the Pt metal domains acted as an electron sink, promoting secondary metal deposition over the existing one. In the case of saturated Pt domains at the edges of the CdS crown, the Au growth pattern is due to the high tendency of stable Au–Se bond formation at the CdSe–CdS interface and steric hindrance causing unsaturated S atoms to be less available since it is already occupied by Pt. On the other hand, on less crowded Pt decoration, small Au island were observed on the edges as well, as the influence of steric interference became less relevant.

Along with the variety of deposited metal component combinations, heterostructures of the semiconductor components have also been realized in the formation of HNPs. As was mentioned throughout the current and previous sections, different combinations of metal chalcogenides have been reported. These kinds of heterostructures reveal a different band alignment and therefore different electronic profile such as type I, type II, and quasi type II. The final energy band alignment of the HNPs with the specific metal deposition can influence and dictate the performance of the HNPs in its designated application, where different applications require different energy band design. The effect of the material combination on the HNPs characteristics and on their utilization in various applications will be discussed in the following sections.

## 3 Synergetic Properties of HNPs: Whole Is Greater than the Sum of the Parts

HNPs hold unique properties which arise from the combination of two disparate materials with distinct different physical and electronic characteristics. The hybrid properties are either combined attributes of each of the segments that present both elements properties at the same single system or often a synergistic combination manifesting new properties that are a consequence of the semiconductor–metal interface. Notably, the resulting properties of the combined materials depend not only on their type but also on the size and morphology of the HNPs and the materials interface.

### 3.1 Optical Properties

#### 3.1.1 Absorbance

Conjugation of semiconductor and metal segments in a single nanocrystalline system may introduce new hybrid electronic states at the semiconductor–metal interface. Therefore, it is expected that the overall optical and electronic properties of such hybrid systems will be affected in comparison to their components. In HNPs, the coupling between excitons and plasmons becomes especially strong near resonance in which the exciton energy lies in the vicinity of the plasmon peak which results in broadening and a shift of the first exciton transition in the semiconductor spectra.^136^ This optical alternation is highly pronounced in HNPs combining semiconductors where the excitonic band-gap transition and the plasmonic features of the metal segment are spectrally separated from each other.

**Figure 17:**
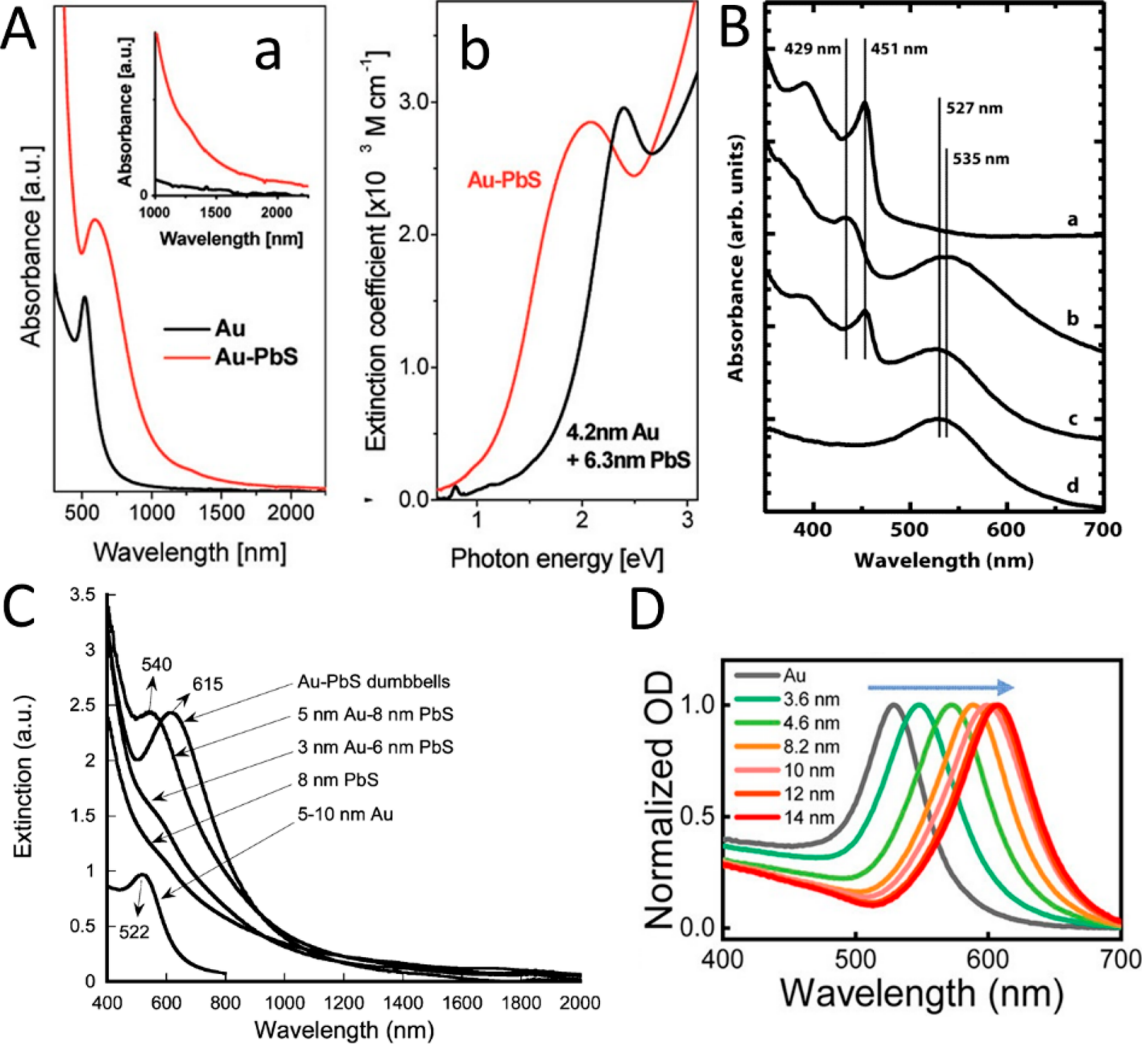
Absorption enhancement and spectral shift. (A) (a) Absorption spectra of 4.2 nm Au NCs (black) and Au–PbS core–shell HNPs with 2.6 nm thick PbS shells (red).( Inset) Magnified near-IR part of the spectra. (b) Extinction coefficient of Au–PbS core–shells (red) and extinction coefficients for noninteracting 4.2 nm Au and 6.3 nm PbS NCs (black). (B) Comparison of the absorption spectra of (a) free CdS NRs, (b) CdS–Au hybrid NRs, (c) physical mixture of free CdS NRs and Au NCs, and (d) free Au NCs coated with tetraoctylammonium bromide. CdS exciton peak and Au plasmon peak in the HNPs are shifted with respect to the individual components and a physical mixture of the components. (C) Absorption spectra of Au–PbS HNPs exhibit red shifts of the SPR frequency compared to pure Au NCs. (D) Steady-state absorption spectra of the uncoated Au core and Au–CdS HNPs with various shell thicknesses. Blue arrow indicates the direction of the increasing shell thickness. (A) Adapted with permission from ref (137). Copyright 2008 American Chemical Society. (B) Adapted with permission from ref (26). Copyright 2006 American Chemical Society. (C) Adapted with permission from ref (82). Copyright 2006 American Chemical Society. (D) Adapted with permission from ref (142). Copyright 2021 American Chemical Society.

Experimental observations of exciton–plasmon coupling were seen for Au–PbS core/shell HNPs^137^ and CdS–Au NRs (Figure 17).^26,29^ In the latter hybrid nanosystem, the native CdS excitonic transitions as well as the Au surface plasmon resonance (SPR) modes are well separated and still discernible. Still, some changes are seen (Figure 17B). A blue shift to higher energy accompanied by optical broadening was observed in the hybrid form in comparison to pristine CdS semiconductor NRs. Suppression of the excitonic features following Au growth was also observed and investigated by Zamkov et al. on similar CdS–Au HNPs with different metal domain sizes synthesized by a thermal reduction procedure.^118^ This phenomenon was attributed to the tunneling of CdS excited charge carriers into Au domains.^138^ This property is dependent on the size of the Au tips where for small-diameter Au tips, the delocalization of CdS electrons into the Au is limited to a few nanometers, which is insufficient to alter the character of quantum confinement in CdS. On the other hand, larger Au domains permit larger electronic delocalization into the metal, which in turn leads to the “washing out” of the excitonic transitions.

Along with the excitonic alternation, the plasmonic resonance in HNPs is red shifted compared to free-standing metal nanoparticles. This shift is ascribed to the change in the dielectric constant of the nearby surroundings of the metal component. In general, the plasma frequency of the bulk metal, *ω*_P_, is deduced with the Drude model^139^
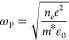
3where *n*_e_ is the electron concentration, m* is the effective mass of the electron, *e* is the charge of an electron, and ε_0_ is the permittivity of vacuum. For a metal nanoparticle, the surface plasmon resonance frequency, *ω*_SPR_, can be expressed by the following relation according Mie theory^140^
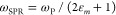
4where *ε*_*m*_ is the environment dielectric constant. Since the plasmonic resonance is inversely dependent on the dielectric constant of the environment, a coupled semiconductor domain with higher refractive index will result in a decrease in the plasmonic frequency and a spectral red shift. This shift is much more pronounced in core/shell HNPs structures due to the spherical symmetry as was demonstrated for PbS–Au core/shell and dumbbell-like nanoparticles. SPR shift to lower energies between 20 and 100 nm was measured, respectively (Figure 17C).^82,137^ The extent of the plasmonic shift can be tuned through changing the type and shell thickness of the semiconductor component. Such control was demonstrated for Au–Ag, Ag_2_S–Au, and CdS–Au core/shell NRs that show both transverse and longitudinal SPR signals.^141^ A blue shift was observed upon growth of the metallic silver shell around the Au nanorods with respect to the Au rods SPR signal. For the CdS shell, a red shift is seen, which increased with shell thickness. A similar trend was recently reported for Au–CdS core/shell nanoparticles with different shell thickness from 3.6 to 14 nm, revealing a plasmon shift from 548 to 608 nm (center wavelength of the Au LSPR band), respectively (Figure 17D).^142^ For the case of matchstick-like HNPs such as Au-tipped CdS, the plasmonic shift was found to be more moderate, up to 10 nm,^26,29,143^ as the surrounding environment of the gold tip is not homogeneous, where the tip is directly exposed to both the semiconductor domain on one side and the solvent on the other side. Additional manipulation of the plasmonic features of the metal component was reported by synthesizing the Cu_2_Se shell over Au NRs. In the absence of oxygen, the spectrum of the Au–Cu_2_Se core–shell HNPs exhibit metallic behavior, where the characteristic transverse and longitudinal plasmon bands of the AuNR cores are dominant. Under aerobic conditions, oxidation of the semiconductor shell resulted in vacancies (Cu_2–*x*_Se) that lead to diminishing of the longitudinal plasmon band of the Au due to the gradually overlapping and ultimately dominating transverse mode of the shell.^144^

**Figure 18:**
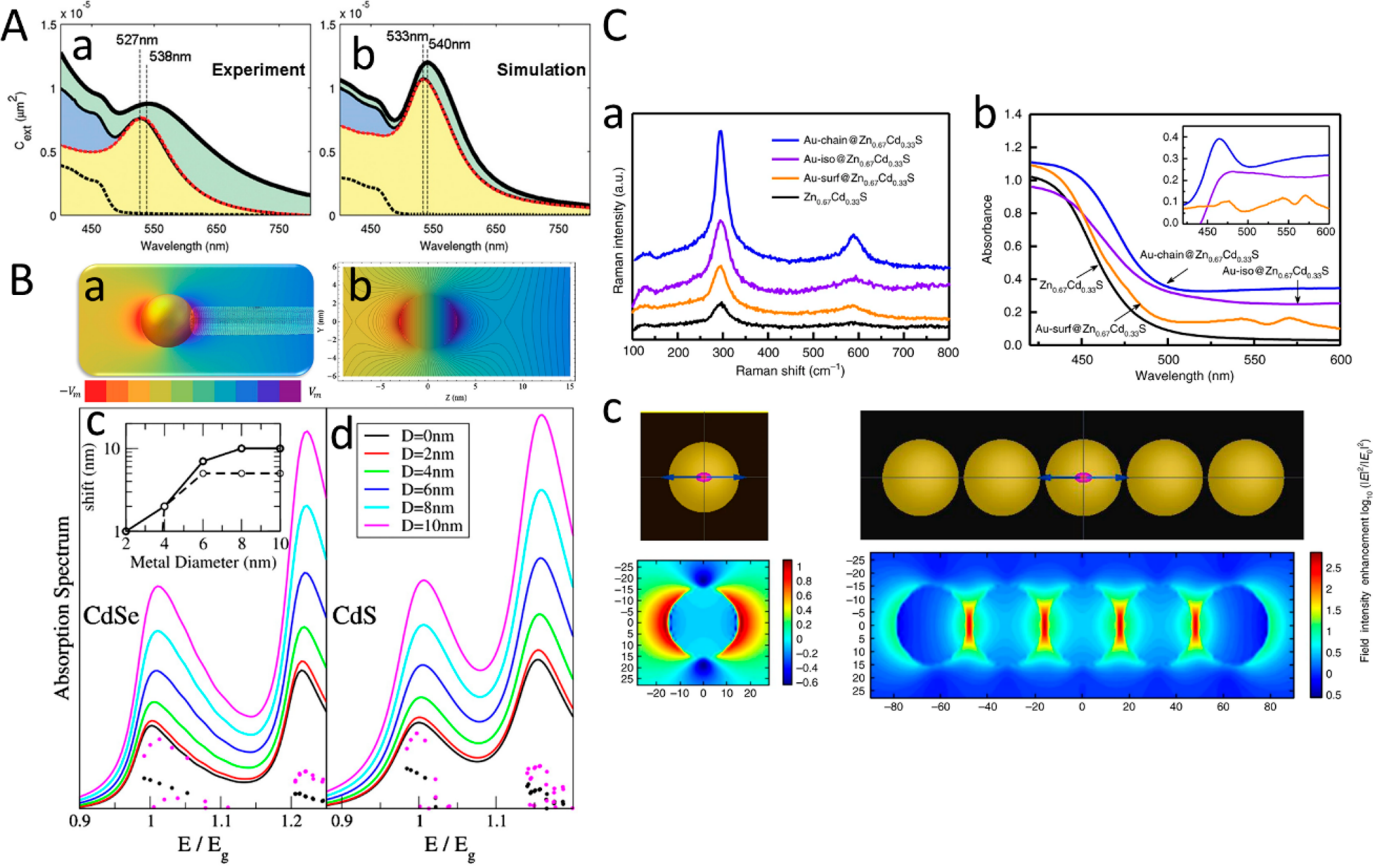
Optical properties of HNPs. (A) Extinction cross section (in μm^2^) as a function of wavelength for bare CdS NRs (dashed black curve), Au bare NCs (red dotted curve and yellow area), mixture of Au NCs and CdS NRs with dimensions similar to those in the hybrid NRs (yellow plus blue area, narrow black curve), CdS–Au HNP (thick black line), and difference between HNP versus the mixture of its components (green area). (a) Experimental absorption measurements. (b) Discrete dipole approximation simulations. (B) (a) Schematic illustration of the geometry of a metal-tipped nanorod used to model the effect of a near field on the optical transitions of a nanoscale system. (b) Isopotential contour lines of the near-field potential generated by a gold nanoparticle of 6 nm diameter. *V*_m_ is the maximal potential on the surface of the gold nanoparticle. Absorption rates in (c) CdSe and (d) CdS 20 nm NRs. Lines represent results for different metal tip diameters ranging from 0 (black) to 10 nm (magenta) in steps of 2 nm. Relative oscillator strengths for the strongest transitions are shown for individual transitions for the smallest and largest metal tip sizes. (Inset) Absorption maximum (blue) shift as a function of metal tip diameter. Solid and dashed lines correspond to CdSe and CdS, respectively. (C) Plasmon-induced local electromagnetic field effect: (a) Raman spectra, (b) UV–vis diffuse reflection spectra of pure Zn_0.67_Cd_0.33_S and Zn_0.67_Cd_0.33_S–Au with different spatial arrangement of Au NCs. (c) FDTD simulation of the near-field distributions of a single Au NC and five coupled Au NCs. (A) Adapted with permission from ref (143). Copyright 2011 American Chemical Society. (B) Adapted with permission from ref (154). Copyright 2012 PNAS. (C) Adapted with permission from ref (157). Copyright 2019 Nature Publishing Group.

Measurements of extinction cross section as a function of wavelength of the CdS–Au HNP and a mixture of its individual components, CdS NRs, and Au nanoparticles showed a red-shifted plasmon peak from 527 to 538 nm by about 50 meV (Figure 18A).^143^ This observed shift, as explained above, is related to the larger real part of the refractive index of the semiconductor compared to that of the solvent surrounding the metal tip. Comparison with discrete dipole approximation (DDA) simulations on HNP (Figure 18A-b) with the same dimensions taking into account only electrodynamic interactions by describing the individual components by their independent dielectric function showed qualitative agreement with the experimental measurements. Other different HNP architectures including Au–CZTS^53,145^ and Au–Bi_2_S_3_^98^ have presented a similar red shift trend yet with larger shifts, 60–100 and 40 nm, respectively. Such significant shift may suggest more efficient coupling of the metal plasmon with the semiconductor exciton. However, for HNPs where the optical features of the semiconductor and metal are in resonance such as CdSe–Au HNPs, the simplistic calculated electrodynamic treatment could not reproduce the observed absorption spectra. Typically, the absorption spectra of such HNPs exhibit very broad features with the tails extending to the low-energy region in their spectra.^1,146−149^ The stronger coupling and interactions of both components may lead to a strong mixing of the electronic states that an electrodynamics approach solely cannot predict.

Additionally, upon excitation of free electrons by incident irradiation, the metal component can induce an intense electric field in its vicinity.^150^ The proximity of the metal domain to the semiconductor component allows near-field effects that can lead to enhanced absorption in the semiconductor.^151^ This field enhancement is maximized when these HNPs are irradiated at the corresponding plasmon resonance frequency.^152^ The near-field effect is expected to induce an enhancement in the oscillator strength of the dipole-forbidden transitions^153^ and thus may control the optical selection rules. Jain et al. presented atomistic simulations of the oscillator strengths of individual transitions in matchstick-like CdSe–Au and CdS–Au NRs (Figure 18B). A gradient resonant electric field propagates along the nanorod long axis and allows quadrupole-induced transitions and even higher multipolar order transitions, which are forbidden under far-field selection rules.^154^ This phenomenon was experimentally demonstrated on Au–PbS core/shell HNPs, where the absorbance cross section was enhanced by ∼28% compared to the measured absorbance of noninteracting separated Au and PbS nanocrystals.^137^ Generally, the electric field enhancement strongly depends on the size and spacing between adjacent metal domain, as was reported for free-standing metal nanoparticles.^155,156^ Recently, deposition of a Au nanochain (small distance between adjacent metal domains) on Zn_0.67_Cd_0.33_S NPs has shown enhanced formation of electron–hole pairs that led to improved photocatalytic properties in comparison to isolated Au metal domain decoration (3.5 times higher).^157^ This was attributed to the strong plasmon coupling of the chain structure inducing highly intense and localized electromagnetic fields which in turn enhances the semiconductor segment absorption. As shown in Figure 18C, this enhancement was verified experimentally by Raman and UV–vis absorption measurements along with theoretical simulations using a three-dimensional finite difference time domain (FDTD) methodology.

#### 3.1.2 Fluorescence Enhancement

A well-known and highly applicable optical property of semiconductors is it fluorescence via spontaneous emission. When conjugating plasmonic metal and semiconductor nanomaterials into a single system, this radiative process can be either enhanced or quenched depending on the hybrid structural characteristics. These effects are strongly dependent on the distance between the metal and the fluorescent semiconductor NC, on the nature of the semiconductor–metal interface, and on the spectral overlap between the SPR and the emission spectra.

Fluorescence enhancement is typically associated with controlled separation between the two material components as was explored in molecule–metal nanoparticle interfaces.^158^ The enhancement effect can be attributed to either of the two following origins: first, excitation enhancement, which relates to the increased excitations in the presence of an SPR electric field, also associated with the enhanced absorption discussed in the previous section and to the surface-enhanced Raman scattering (SERS) phenomena;^159^ second, emission enhancement, which is affected by the coupling of the SPR field and the transition dipole moment of the emitting semiconductor NC, leading to an increased radiative rate.^160^ The fluorescence enhancement is accompanied by shortened radiative lifetimes due to the inverse relation of lifetime and the radiative rate, which is proportional to the field enhancement factor.^161^

**Figure 19:**
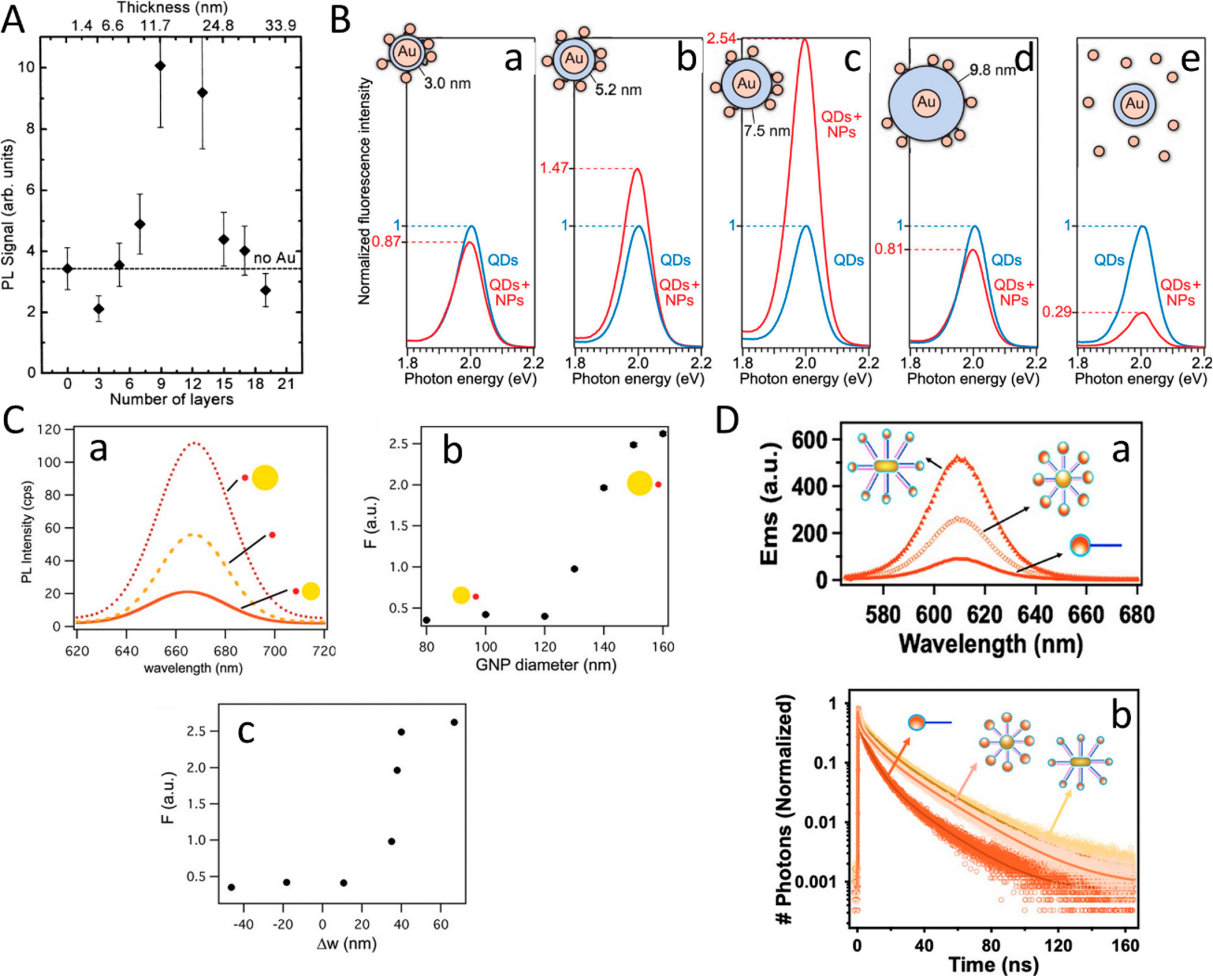
Fluorescence enhancement. (A). Fluorescence intensity of CdSe/ZnS core/shell NPs deposited on Au colloids with defined polyelectrolyte spacer versus number of polyelectrolyte layers between NPs and gold colloids. Horizontal dashed line shows the PL intensity of the same amount of NPs deposited on glass without gold colloids (reference sample). (B) (a–d) Selected fluorescence spectra (red) of Au–CdS HNPs for shell thicknesses 3.0, 5.2, 7.5, and 9.8 nm, normalized to the corresponding NPs fluorescence spectra (blue). (e) Unlinked NPs with Au/CdS NPs (5 nm shells). (C) (a) Fluorescence spectrum of CdTe/CdS NCs measured in the vicinity of Au NP of 80 nm (solid line), without Au NP (dashed line), and in the vicinity of Au NP of 140 nm (dotted line). (b) Modification factor of fluorescence *F* as a function of the diameter of the Au NPs. (c) Modification factor of fluorescence *F* as a function of Δ*w* = λ_LSPR_ – λ_NC_, difference between the plasmon resonance wavelength and the NCs emission wavelength. (D) (a) CdSe/CdS NPs Au NRs (plain orange), CdSe/CdS with Au NPs (circle orange), and CdSe/CdS with Au NRs (triangle orange) assemblies. Average number of satellites is 5 ± 2 for NCs–Au NPs and 13 ± 4 for NCs–Au NRs assemblies. (b) Normalized fluorescence lifetime decays of an ensemble of hybrid assemblies with 30 base pair DNA (∼10 nm interparticle separation) CdSe/CdS NPs Au NRs (dark orange), CdSe/CdS with Au NPs (light orange), and CdSe/CdS with Au NRs (medium orange) assemblies. (A) Adapted with permission from ref (162). Copyright 2002 American Chemical Society. (B) Adapted with permission from ref (169). Copyright 2019 American Chemical Society. (C) Adapted with permission from ref (171). Copyright 2010 American Chemical Society. (D) Adapted with permission from ref (167). Copyright 2022 American Chemical Society.

One of the most prominent factors to control the fluorescence enhancement is the distance between the plasmonic metal and the emitting semiconductor components.^161−168^ Demonstration of the distance effect on emission enhancement in semiconductor–metal nanoparticles was reported for CdSe/ZnS core/shell nanoparticles deposited on Au colloids with a defined polyelectrolyte spacer allowing controlled distance between the semiconductor nanoparticles and the gold films.^162^ Maximum enhancement by a factor of 5 was achieved for a 9-layer spacer (∼11 nm) due to a local enhanced electromagnetic field around the metal nanostructures (Figure 19A). An additional example of controlling enhanced fluorescence by varying the distance between the two components was achieved by introducing different concentrations of Au nanoparticles to CdTe nanoparticles in aqueous solution, both being negatively charged. Their identical surface charges induce strong interparticle repulsion. By controlling the concentration and feed ratio of Au and CdTe, the interparticle distance between them can be tuned and hence the degree of fluorescence enhancement of the semiconductor NCs can be controlled. A 3-fold fluorescence enhancement of the Au–CdTe mixed solution was obtained compared to a CdTe NCs solution in the absence of Au NPs.^164^

Colloidal Au/CdS and Au/ZnS core/shell HNPs linked with CdSe/CdS NPs were also used to study the distance dependence of fluorescence enhancement. A significant fluorescence enhancement was observed for 6–8 (CdS) and 8–10 nm (ZnS) shell thicknesses, with the yields dropping for thinner and thicker shells (Figure 19B).^169^ The authors assigned this effect mostly to the distance-dependent energy transfer rate with a minor contribution of spectral overlap of excitonic and plasmonic features of this hybrid assembly. A similar methodology of absorbed CdSe/ZnS semiconductor NPs on the surface of Ag/SiO_2_ core/shell metal–semiconductor HNPs revealed a similar distance-dependent behavior, although in this case the effect was explained by excitation enhancement.^165^

Additional parameters can influence the measure of fluorescence enhancement as was widely reported previously in the literature. The number of Au domains in the vicinity of emitting semiconductor nanostructures was found to be a key factor in this optical enhancement as was reported for CdTe NWs coaxially surrounded by Au NPs^161^ as well as for ZnCdSeS–Au HNPs.^170^ The collective character of an ensemble of interacting Au NPs promotes a strong electromagnetic field and therefore more efficient fluorescence enhancement compared to single or isolated Au domains.

Moreover, the size and shape of the plasmonic metal component can affect the degree of fluorescence enhancement. Royer et al. reported on a transition from a fluorescence quenching regime to enhancement behavior via the increased size of the Au metal NPs in an assembly with CdTe/CdS NCs while keeping a distinct distance of 3 nm between the two components.^171^ Au NPs with diameters in the range of 60–160 nm were investigated. Au NPs larger than 130 nm in diameter yielded fluorescence enhancement, up to 260% for 160 nm diameter Au NPs compared to the reference sample. Smaller sized Au NPs (<130 nm) caused fluorescence quenching (Figure 19C). The authors correlated this behavior with the SPR extinction, deducing that the extinction maximum must be red shifted with respect to the CdTe/CdS NC photoluminescence wavelength to obtain fluorescence enhancement (Figure 19C-c).

The shape of the plasmonic metal was also shown to affect the fluorescence enhancement.^172^ Using a rod-like structure of the plasmonic absorber induced stronger enhancement compared with spherical nanoparticles. Hybrid DNA-based assembly of CdSe/CdS with Au NPs and Au NRs showed a significant fluorescence enhancement of 15–75% depending on the different HNPS architectures in comparison to nonconjugated semiconductor NPs.^167^ However, Au rod structures exhibit a greater magnitude of fluorescence enhancement (75% vs 48% for rods and spheres, respectively) due to the strong localization of the near field at the nanorod tips (Figure 19D).

Along with the contribution of the metal component shape and size to the fluorescence properties, the effect of the morphology and architecture of the complete hybrid nanosystem has been investigated. Ramamurthy and co-workers reported on the improved up to 9-fold luminescence enhancement by Au body-decorated CdS NRs, while for the case of CdS–Au NPs, a 5-fold enhancement was observed.^173^ Different metal sites deposition led to alternated photoluminescence. This effect was demonstrated by Au–AgCdSe HNPs with three different architectures. A single Au site at the apex of the AgCdSe segment in a microphone-like structure revealed the strong fluorescence in comparison to the negligible emission of the double Au sites on both apexes (dumbbell-like structure) and single site in the formation of a toothbrush-like structure.^174^ Although quenching processes likely occurred in all nanostructures, the fluorescence enhancement of the mike-like architecture outcompetes these relaxation routes due to the overlap energies of the Au SPR and the band edge of CdSe exciton absorption which promote coupled field enhancement from the Au portion of the nanorods.

#### 3.1.3 Fluorescence Quenching

**Figure 20:**
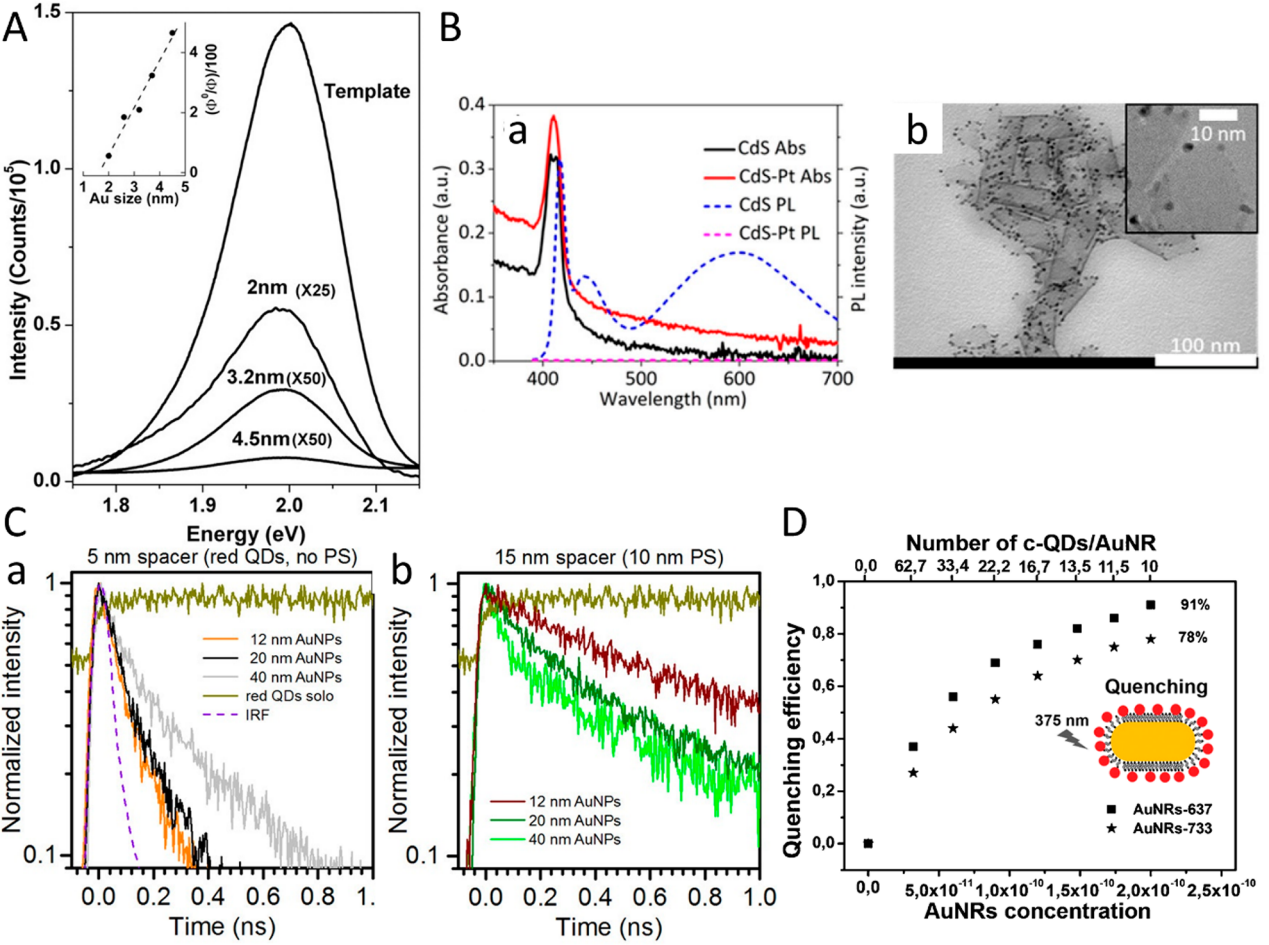
Fluorescence quenching. (A) Fluorescence spectra of CdSe–Au hybrid nanodumbbells. Traces were multiplied by 25, 50, and 50 for the 2, 3.2, and 4.5 nm Au tips, respectively, for clarity. (Inset) Plot of relative fluorescence yield for template (Φ^0^) over Au rod (Φ) versus Au tip size. (B) (a) Absorption (Abs) and static-photoluminescence (PL) spectra of CdS NPLs and CdS–Pt hybrid NPLs. (b) TEM images of CdS −Pt hybrid NPLs. (Inset) TEM images showing preferential deposition of Pt at the CdS NPL edge. (C) Comparison of PL decays from CdSe/CdS/ZnS core/shell/shell NPs with different-sized NPs with (a) 5 and (b) 15 nm fixed PS spacer thicknesses. (D) Comparison of quenching efficiencies at different concentrations of AuNRs with longitudinal localized surface plasmon resonance at 637 (squares) and 733 (stars) with CdSe/ZnS NPs. (A) Adapted with permission from ref (1). Copyright 2004 AAAS. (B) Adapted with permission from ref (61). Copyright 2018 American Chemical Society. (C) Adapted with permission from ref (183). Copyright 2016 American Chemical Society. (D) Adapted with permission from ref (166). Copyright 2014 American Chemical Society.

As discussed above, the photophysical properties of HNPs are dictated by the unique interactions of the semiconductor–metal nanojunction. As was deduced for the case of fluorescence enhancement, the nanoscale separation between the two components of a hybrid nanosystem can also induce quenching effects as the distance between the semiconductor and the metal becomes smaller. In a similar manner to the enhancement effect, this quenching mechanism is attributed to the strong plasmon–exciton coupling and interactions which increase the nonradiative routes. Moreover, direct growth of the metal component on the semiconductor reduces the distance and the barrier between them, typically causing fluorescence quenching. This reduction in fluorescence intensity is assigned to charge transfer from the excited semiconductor part to the metal and thus increases the nonradiative rate accompanied by a shorter lifetime. Such effect was observed and reported previously in the literature for several different HNPs with different material combinations and a variety of morphologies. Prasad and co-workers demonstrated this quenching effect via PbS and PbSe growth on Au NPs with formation of both a dimer and a core/shell.^82^ Similar results were obtained for CdSe–Au dimers^175^ and Au–CdS^176^ core/shell HNPs. Numerous reports on 1D hybrid nanorod structures observed similar quenching behavior, including CdSe–Au,^1,115,146,147,177^ CdSe–Pt,^146,178^ CdSe–Co,^54^ CdS–Au,^26,32,138,177^ CdS–Pt,^179^ CdSe/CdS–Au,^33^ CdSe/CdS–Ag,^51^ ZnSe/CdS–Pt,^46^ and CdTe–Au.^180^ An example of the CdSe nanodumbbell quenching trend with increased metal deposition is provided in Figure 20A.

Recently, with the synthetic development of 2D HNPs, hybrid structures such as nanosheets or nanoplatelets showed the same fluorescence quenching property as was demonstrated for CdS–Au,^61^ CdSe–X (X = Au, Pd, Pt),^41^ CdSe/CdS–Au,^42,43^ and CdSe/CdS–Pt^43^ NPLs and CdSe–Pt^181^ and CdS–Ni^74^ nanosheets (Figure 20B). Although all studies report on the reduction in the fluorescence intensity upon metal deposition, there are differences regarding the charge carrier dynamics of this quenching process as will be further discussed in following sections.

As with fluorescence enhancement discussed above, quenching of HNPs emission has been shown to be size and shape dependent. An early example of the Au metal domain size effect on the fluorescence intensity was reported for ZnSe/CdS NRs where a sharp decrease in fluorescence intensity (up to 500-fold for a 4.5 nm diameter Au domain) with increasing Au NP size was observed.^1^ Later, this trend was also reported for CdSe–Au HNPs with different sizes of the semiconductor as well as the metal components.^182^ However, this monotonic behavior was shown to be further effected when taking into account different separating distances between the metal and the semiconductor segments. Time-resolved fluorescence measurements of HNPs with different sizes of Au NPs bound to CdSe/CdS/ZnS core/shell/shell NPs with different lengths of spacer showed that for the smallest separations, the smallest Au NPs yield the fastest decay, while for larger separations, the largest AuNPs lead to the fastest decay (Figure 20C).^183^

Besides the size of the metal domain, Haldar et al. reported on additional shape effect comparing different sizes of Au NPs with Au nanorod structures conjugated to CdTe NCs. While quenching values by 47%, 65%, and 73% were measured for 2 ± 0.3, 9 ± 0.4, and 17 ± 0.2 nm Au NP, respectively, quenching by 86% was obtained for the Au NR conjugates.^180^ Other studies have presented the influence of the number of either metal or semiconductor sites on the fluorescence efficiency. Mattoussi and co-workers studied the effect of increased Au NPs to CdSe/ZnS NPs ratio along with the semiconductor size and the separation distance between the components. For all semiconductor sizes and separating distances, an increasing ratio led to a higher quenching efficiency and faster decay lifetime.^184^ The authors attributed the quenching mechanism to the strong energy transfer from the emitting NPs to the metal domains affecting mostly the nonradiative channel with negligible influence on the radiative channel. The effect of the inverse ratio of increased number of semiconductor NPs to a single plasmonic metal site was demonstrated by an Au nanorod coated with CdSe/ZnS NPs.^166^ In this work, a shortened lifetime and increased quenching efficiency were obtained for decreasing semiconductor to metal ratios from 40% to 91% for from 62 to 10 CdSe/ZnS NPs for a single Au NR, respectively (Figure 20D). These observations emphasize that the resonance between the exciton and the plasmon strongly influences their coupling and therefore their photophysical properties.

Therefore, according to the above understandings regarding both fluorescence enhancement and quenching as were deduced from the extensive investigations of these optical properties of HNPs, several rules of thumb can be established for designing either enhanced or quenched emitting HNPs systems. While the size and shape of both the metal and the semiconductor components can contribute to the extent of desired optical property and enhance the overall fluorescence effect of both enhancement and quenching, the more pronounce parameters are the distance between the two segments and the spectral overlap. The first can be set by sorting the suitable synthetic pathway to achieve either sufficiently separated HNPs by inserting a separating layer in the form of SiO_2_ or organic linkers (polymers or molecules) that will lead to fluorescence enhancement or deposing one material on the surface of the other, allowing charge transfer and therefore fluorescence quenching. The second parameter of spectral overlap can be tuned by the choice of materials to be conjugated efficiently to promote near-field effects that would enhance the absorption of the adjacent component and permit effective energy transfer.

### 3.2 Electrical Transport Properties

**Figure 21:**
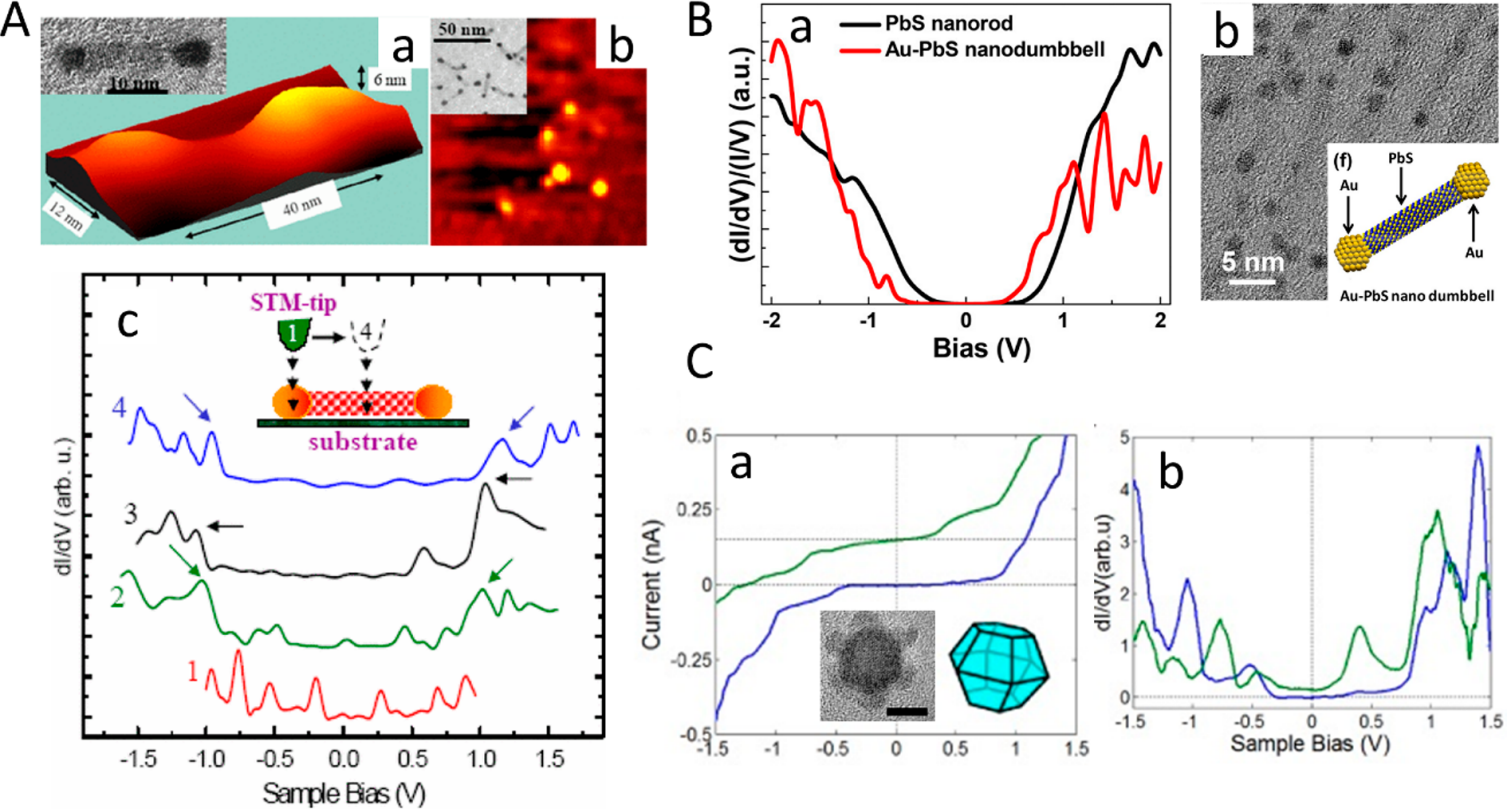
Electrical transport properties. (A) (a) STM topography image of a single Au-tipped CdSe nanorod (nanodumbbell) taken with *V*_B_ = 2 eV. (b) STM current image (150 × 150 nm^2^) acquired at a sub-band-gap bias (0.5 eV) showing the gold tips of three nanodumbbells. (Insets) High-resolution (a) and low-resolution (b) TEM images of nanodumbbells. (c) Evolution of tunneling spectra (at 4.2 K) along the nanodumbbells, moving from the Au dot (curve 1) to the rod center 4. Curves 2 and 3 were taken on the CdSe rod, about 1 and 4 nm from the dot, respectively, showing subgap structure attributed to localized surface states induced by the Au–CdSe contact. Arrows mark the onsets of the CdSe conduction and valence bands. (Inset) Scheme of the measurement configuration. (B) (a) Comparison of (d*I*/d*V*)/(*I*/*V*) versus *V* spectra of a PbS nanorod (black curve) and PbS–Au nanodumbbell (red curve) measured at center positions. (b) TEM image of PbS–Au nanodumbbells showing Au contacts at both tips of the PbS nanorod. (Inset) Schematic of PbS–Au nanodumbbell. (C) (a) Tunneling *I*–*V* curves and (b) corresponding d*I*/d*V* – *V* spectra of a Cu_2_S–Ru hybrid vary greatly along the surface of the nanocage, exhibiting either the pristine semiconductor band gap with in-gap states (e.g., the blue curves) or reduced gaps with enhanced in-gap DOS (e.g., green curves) as well as metallic-like single electron tunneling characteristics. (Inset) TEM image and schematic of hybrid Cu_2_S–Ru nanocages. (A) Adapted with permission from ref (185). Copyright 2005 American Physical Society. (B) Adapted with permission from ref (186). Copyright 2018 American Chemical Society. (C) Adapted with permission from ref (187). Copyright 2012 IOPscience.

The semiconductor–metal interface also manifests unique electrical properties. Besides the electronic characteristics of each of the components on its own, synergetic features arise due to the conjugation of these two disparate materials. Understanding the synergistic effects at the semiconductor–metal nanojunction may lead to their utilization in various electrical applications including their use as electrical contacts and building blocks for nanoelectronic devices. Early study of the electronic synergistic effects was conducted on a single CdSe–Au nanodumbbell structure by cryogenic scanning tunneling microscopy and spectroscopy (STM and STS, respectively).^185^ Since the derivative of the tunneling current to voltage is proportional to the local density of states (DOS), the method offers a unique view of the spatial dependence of the electronic levels. Therefore, by scanning along the nanorod structure, the changes in the local DOS can be visualized. As can be seen in Figure 21A, at the center of the rod, STS showed an energy gap value similar to that of a CdSe rod. On the metallic site, a modified Coulomb staircase structure, signifying single-electron charging events in the metal NP, was observed. At the semiconductor–metal interface, a subgap structure was observed up to 5 nm from the metal domain, indicating the formation of in-gap states in the vicinity of this interface.

A related electronic behavior was observed for PbS–Au nanodumbbells, while measurements at different locations along the length of a pristine PbS NR showed delocalization of both the VB and the CB. Partial delocalization of the conduction band along the nanodumbbell length was observed, whereas the VB was found to be localized to the semiconductor section.^186^ This delocalized conduction band is attributed to the electronic coupling of the Au metallic contact with that of the PbS nanorod and the ability of charge-separation processes. This partial delocalization forms an n-type behavior that is manifested by the shift in the (d*I*/d*V*)/(*I*/*V*) spectrum to the negative bias (Figure 21B). STM/STS studies performed on Cu_2_S–Ru nanocage structures also revealed a semiconductor gap along with in-gap states assigned to metal-induced gap states that developed at the semiconductor–metal interface (Figure 21C), consistent with other reports.^187^

**Figure 22:**
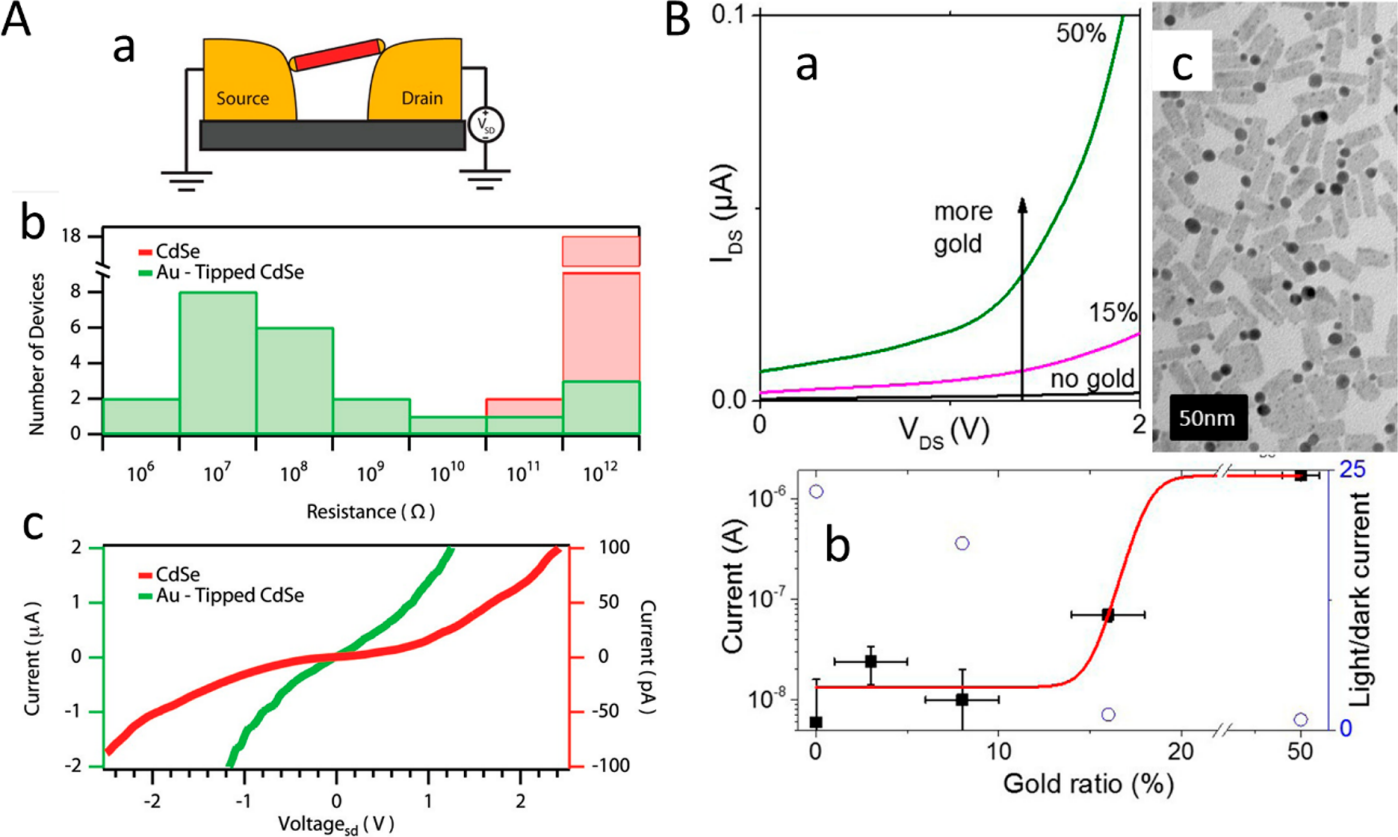
Electrical conduction enhancement. (A) (a) Schematic of a CdSe–Au single-nanocrystal two-terminal device. (b) Histogram of room-temperature device resistance near 0 V applied bias. (c) Representative two-probe *I*–*V* trace of a CdSe device (red) and an Au-tipped CdSe device (green) at room temperature. Note the color-coded axes correspond to picoampere (red) and microampere (green) scales. (B) (a) Current as a function of applied bias for thin film of CdSe NPLs and Au tip–CdSe NPLs. (b) Conductance and photocurrent of the Au tip–CdSe NPLs film as a function of the gold ratio. (c) TEM image of CdSe/CdZnS nanoplatelets functionalized using gold photoreduction. (A) Adapted with permission from ref (188). Copyright 2009 American Chemical Society. (B) Adapted with permission from ref (39). Copyright 2016 American Chemical Society.

The resulting in-gap states at the semiconductor–metal interface leads to an enhancement of the HNPs conductance, as was demonstrated for CdSe–Au nanodumbbells. *I*–*V* measurements of such HNPs compared to the bare semiconductor NR showed superior performance of the former attributed to a lower Schottky barrier in the hybrid structure (about 75% decrease), allowing an average 6-decade increase in conductivity near zero applied bias (Figure 22A).^188^ Charge-transport studies of CdSe–Au NR arrays and networks showed enhanced dark currents and photocurrents that are mediated by the morphology of the hybrid structures.^120,189,190^ Selective Au deposition at the rod apexes resulted in a decrease of the conductivity at room temperature compared to random Au decoration on the rod surface. However, improvement at cryogenic temperature was observed. This phenomenon was assigned to the dominant thermionic emission across the nanosize Schottky barriers at room temperature, whereas at cryogenic temperature, the thermal activation of carriers becomes negligible and the charge transport is affected by charge tunneling and Coulomb blockade that corresponds to single-electron charging events. Similar results were obtained for CdSe–Pt HNPs.^191^ Moreover, a metal domain size dependence was observed where for a 3 nm Pt site dark current was permitted due to increased thermionic and field emission processes, in contrast to small size Pt-decorated HNPs and pristine CdSe NPs that conduct only upon irradiation. Note that since the nanoparticles size is smaller than the depletion width, Schottky barrier formation models might not apply to the metal–semiconductor nanojuction.

Mahler et al. studied CdSe–Au NPLs reporting as well on conduction enhancement by an order of magnitude (Figure 22B).^39^ Yet, the authors withheld assigning this behavior to a reduction of the band-gap energy. Instead, they attributed these observations to tunneling events between metallic tips. Other transport mechanisms were reported for different HNPs depending on their materials combination. Hole conductivity was proposed for Au–PbS core–shell HNP thin films, where electrons are transferred to the metallic core.^137^ In addition, a tunneling mechanism of charge carriers between the metallic cores was suggested to dominate the electron transport in FePt–Pb chalcogenide core/shell HNPs.^192^ In spite of the existence of a relatively long distance between adjacent NPs, the tunneling is enabled by the low effective mass of electrons in the PbS shell as well as the contribution of electron-donating species as adsorbates, which lowers the energy barrier at the FePt–PbS interface.

### 3.3 Light-Induced Charge Separation

One of the most important and potentially applicable properties of HNPs is the formation of spatial charge separation following irradiation and light absorption. This characteristic arises from the conjoining of a semiconductor and a metal in a single HNP. The formed energy band alignment on both sides of the semiconductor–metal interface promotes excited charge carrier transfer across this interface.

#### 3.3.1 Light-Induced Charge Separation Across the Semiconductor–Metal Nanointerface

Typically, the charge-separation process in HNPs includes the following steps. First, absorption of sufficient energy at the semiconductor segment (above the band-gap energy of the semiconductor) creates an excited electron–hole pair (i.e., exciton). Then, excited electrons relax and transfer to the metal domain, whereas the counter charge carriers (e.g., holes) are restricted to the semiconductor region. The described separation process competes with backward recombination processes, including electron–hole recombination and the loss of electrons in the metal, by recombination with the holes in the semiconductor component. The resulting spatial charge separation is the fundamental principle behind the photocatalytic characteristics of HNPs as will be extensively discussed later. This unique synergistic property of HNPs was extensively investigated in various HNPs architectures and morphologies. Generally, the efficiency and the dynamics of this process are dependent on the type, structure, and morphology of both components. Several reviews previously discussed and summarized the kinetics and dynamics of the different relaxation routes of excited charge carriers in HNPs, focusing on the effects of different structural and environmental parameters and their implications for different applications.^6,90,193−195^ In this context, we will provide a brief overview on the dominant parameters that influence charge separation along with highlighting recent findings and understandings of different relaxation routes dynamics in HNPs.

Early evidence of charge separation was observed in the form of fluorescence quenching of emitting semiconductor NPs following metal deposition which was assigned to the relaxation of the excited electrons in the semiconductor through charge transfer to the metal domain. Hence, reduction of the fluorescence lifetime is obtained by forming an additional competitive nonradiative relaxation route (as was discussed in section 3.1.3). An additional primary observation showed that the charge transferred to the metal domain can either be accumulated as in the case of Au and Ag conjugated metals, leading to a Fermi level shift due to the electrons charging energy, or hold Ohmic-like behavior as was reported for Pt deposition.^17,147,196^ A charging effect of the metal domain through charge transfer was demonstrated for CdSe–Au nanodumbbells in aqueous solutions accumulating up to tens of electrons per metal domain following irradiation.^177^ Moreover, an in situ single-particle measurement of Au deposition on CdSe/CdS NRs was able to trace the charging effect of the metal domain with relaxed electrons from the semiconductor conduction band up to Fermi level equilibration with the latter via plasmonic shifts and increased optical intensities in their UV–vis spectra.^65^

To unravel the excited charge carriers dynamics in the charge-separation process, steady-state measurements and time-resolved fluorescence alone cannot differentiate between the different relaxation routes of the different charge carriers (electrons, holes). Therefore, ultrafast transient absorption spectroscopy (TA) is commonly required. Indeed, in the past decade, excited charge carrier relaxation processes in HNPs have been extensively studied by combination of time-resolved fluorescence and TA measurements (refs (28, 29, 32, 46, 61, 74, 117, 134, 138, 147, 179, 181, 197−199)).

**Figure 23:**
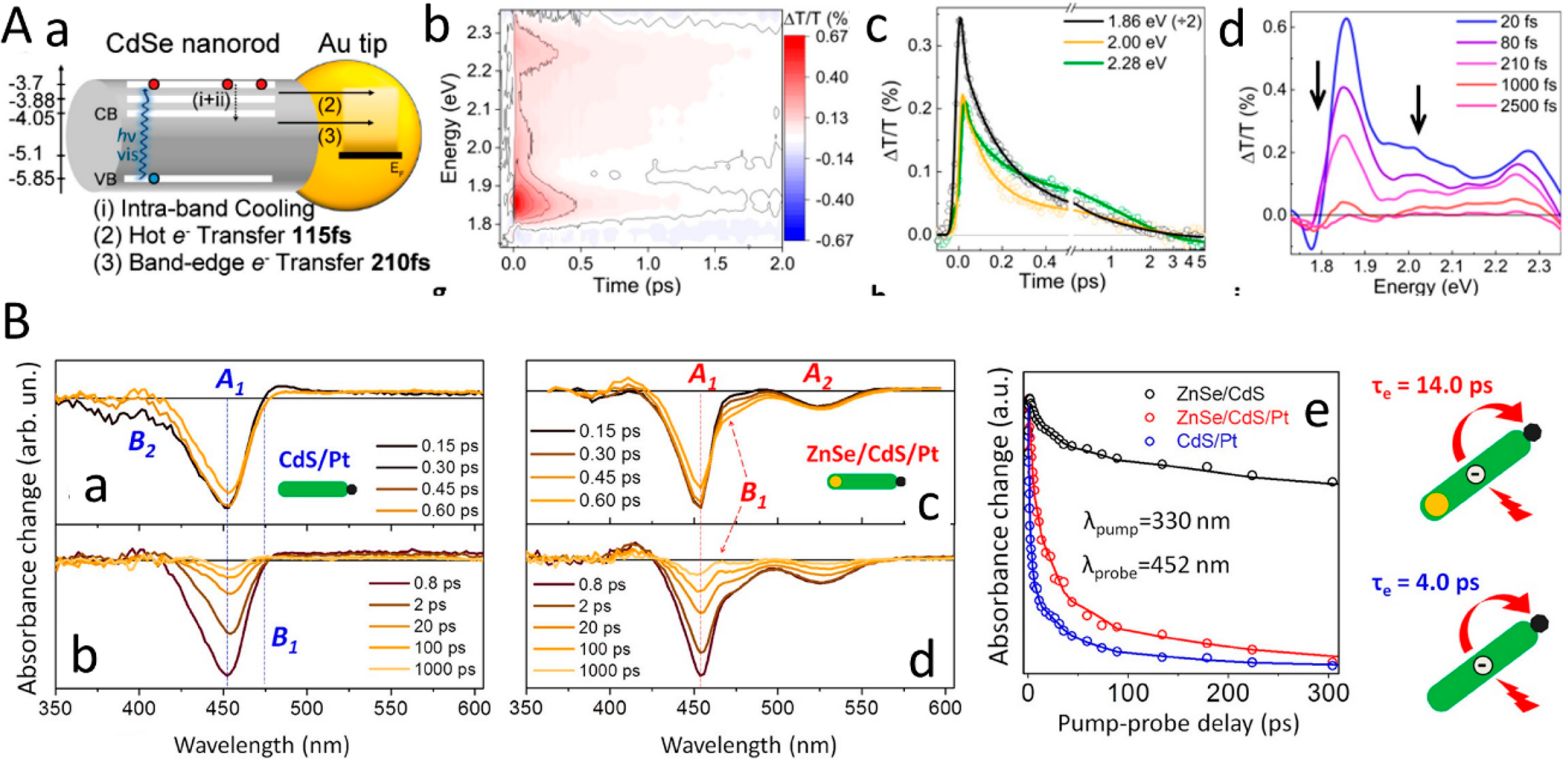
Light-induced charge separation—semiconductor type dependence. (A) (a) Photophysics of CdSe–Au HNPs following broad-band visible, ultrafast hot-electron transfer to gold on a 115 fs time scale competes with intraband relaxation in the CdSe domain and is followed by a slower (210 fs) transfer of band-gap electrons to the gold. (b) TA map of CdSe–Au HNPs in aqueous solution using the visible pulses as a pump and probe (pump fluence of 20.7 μJ/cm^2^). (c) TA kinetics at selected photon energies (dots) and fits to the data (lines). (d) TA spectra at selected time delays. (B) TA spectra of Pt-tipped NRs: (a ,b) CdS–Pt and (c, d) ZnSe/CdS–Pt. Both HNPs exhibit a TA bleach at λ = 455 nm corresponding to the 1S_3/2_(h) →1S(e) transition in CdS (A1) as well as smaller amplitude photoinduced absorption features (B1 and B2). (e) Temporal evolution of the 1S_3/2_(h)1S(e) bleach for ZnSe/CdS (black), ZnSe/CdS–Pt (red), and CdS–Pt (blue) HNPs. (A) Adapted with permission from ref (149). Copyright 2021 American Chemical Society. (B) Adapted with permission from ref (201). Copyright 2012 American Chemical Society.

HNP intraparticle charge separation as well as interparticle hole removal were shown to be affected by the structural, surface, and chemical characteristics of the HNPs including their surrounding environment. For example, CdSe-based HNPs such as CdSe–Au NRs revealed hot and band-gap electron-transfer processes during the first 115 and 210 fs, respectively (Figure 23A).^149^ Similar observations of charge separation within <100 fs were reported for similar HNPs decorated with Au and Pt domains.^134,147,148,200^ However, CdS-based HNPs including CdS–Pt,^179,198^ CdS–Au,^28,138,197^ and CdSe/CdS–Ni^117^ showed ultrafast exciton bleach decays on time scales of at least 1 order of magnitude longer in the range from tens to several picoseconds. This difference can be explained as due to the spectral overlap of the plasmon resonance and the semiconductor exciton that exists in CdSe-based HNPs, whereas in CdS-based HNPs, these features are separated.^143^ This explanation was also assigned to the slower dynamics of the charge-separation process observed for type II ZnSe/CdS–Pt or quasi-type II CdSe/CdS–Pt heterostructured HNPs (∼14 ps) than with the single-phase CdS–Pt HNPs (∼4 ps) (Figure 23B).^201^ Note that although trapping processes of the excited holes at surface defects are extremely fast (0.7 ps),^179^ their transfer from the semiconductor to the surrounding media or to a sacrificial hole scavenger is slower than the analogous electron charge transfer (hundreds of picoseconds to nanoseconds time scale).^202−204^

**Figure 24:**
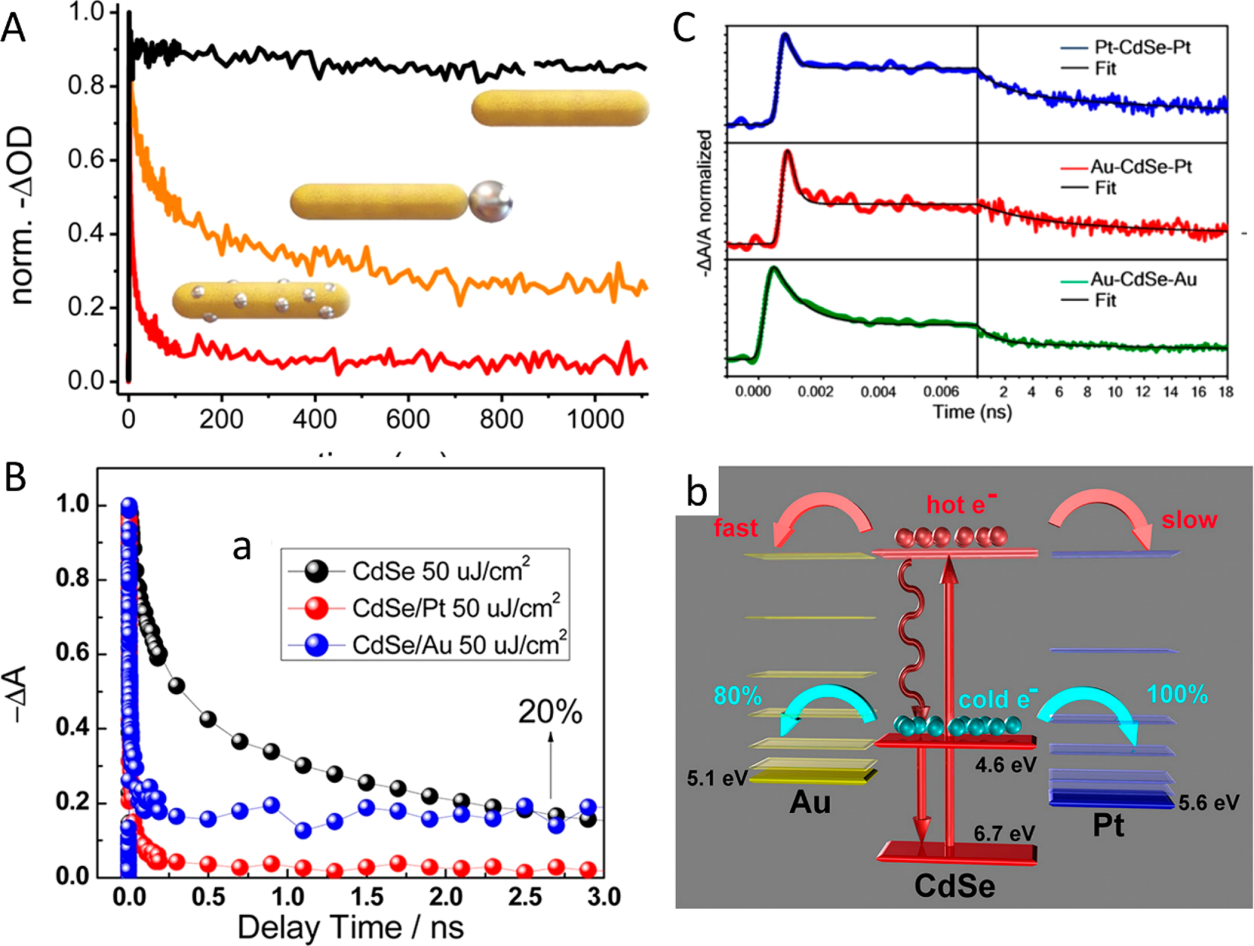
Light-induced charge separation—metal type and decoration architecture dependence. (A) Relaxation of the bleach of the 1S exciton in TA for CdS NRs, CdS–Pt HNPs single-tipped and random multiple sites decoration. (B) (a) TA time traces of CdSe (black), CdSe/Pt (red), and CdSe/Au (blue) rods. (b) Schematic of semiconductor–metal band alignment and relaxation pathways. (C) Normalized TA time traces and fits (black) of Pt–CdSe–Pt (blue), Au–CdSe–Pt (red), and Au–CdSe–Au nanodumbbells (green). (A) Adapted with permission from ref (197). Copyright 2016 American Chemical Society. (B) Adapted with permission from ref (147). Copyright 2013 American Chemical Society. (C) Adapted with permission from ref (134). Copyright 2017 American Chemical Society.

An additional structural effect was reported by Simon et al. comparing random decoration of multiple Pt metal clusters on CdS NRs with selective single-tipped deposition on same nanorods. The relaxation half-life of the excitonic bleach of the multiple-decorated HNPs was found to be much faster than the single-tipped form (4 vs 82 ps for random decoration and tip, respectively).^197^ Moreover, the former architecture showed almost complete decay, implying an effective electron transfer to the metal domains showing 10% of the TA signal remaining after 75 ps, while significant TA signal (>20%) was measured along the entire time scale in the single-metal-tipped HNPs (Figure 24A). These observations were attributed to the shortening of the relaxation path in the presence of multiple metal clusters. However, the recombination rate with the nearby holes increases, thereby canceling any benefit of the increased electron-transfer rate. These opposing trends were also demonstrated on CdS–Au NRs aerogel networks by spectroelectrochemical measurements. Efficient charge carrier separation has been found in the tipped–NR networks resulting in higher negative photocurrent efficiencies compared to the random Au-deposited NR networks where the charge recombination is more pronounced.^106^ A similar behavior for the efficiency of the charge separation was shown to be dependent on the type of the metal component as well. The bleach recovery of CdSe–Au was reported to reach only 80% of the initial bleach signal, indicating remaining excited electrons at the CdSe rod due to suppressed charge transfer, in comparison to 100% bleach recovery in the CdSe–Pt system (Figure 24B).^147^ In addition, Choi et al. reported on improved electron-transfer kinetics for Pt–CdSe–Pt nanodumbbells (263 fs) compared to Au–CdSe–Au and Au–CdSe–Pt compositions (877 and 270 fs, respectively) (Figure 24C).^134^ However, the electron–hole charge recombination rate, at the metal–semiconductor interface, was found to be higher for the Pt-tipped NRs as well, which diminish this separation property faster.

**Figure 25:**
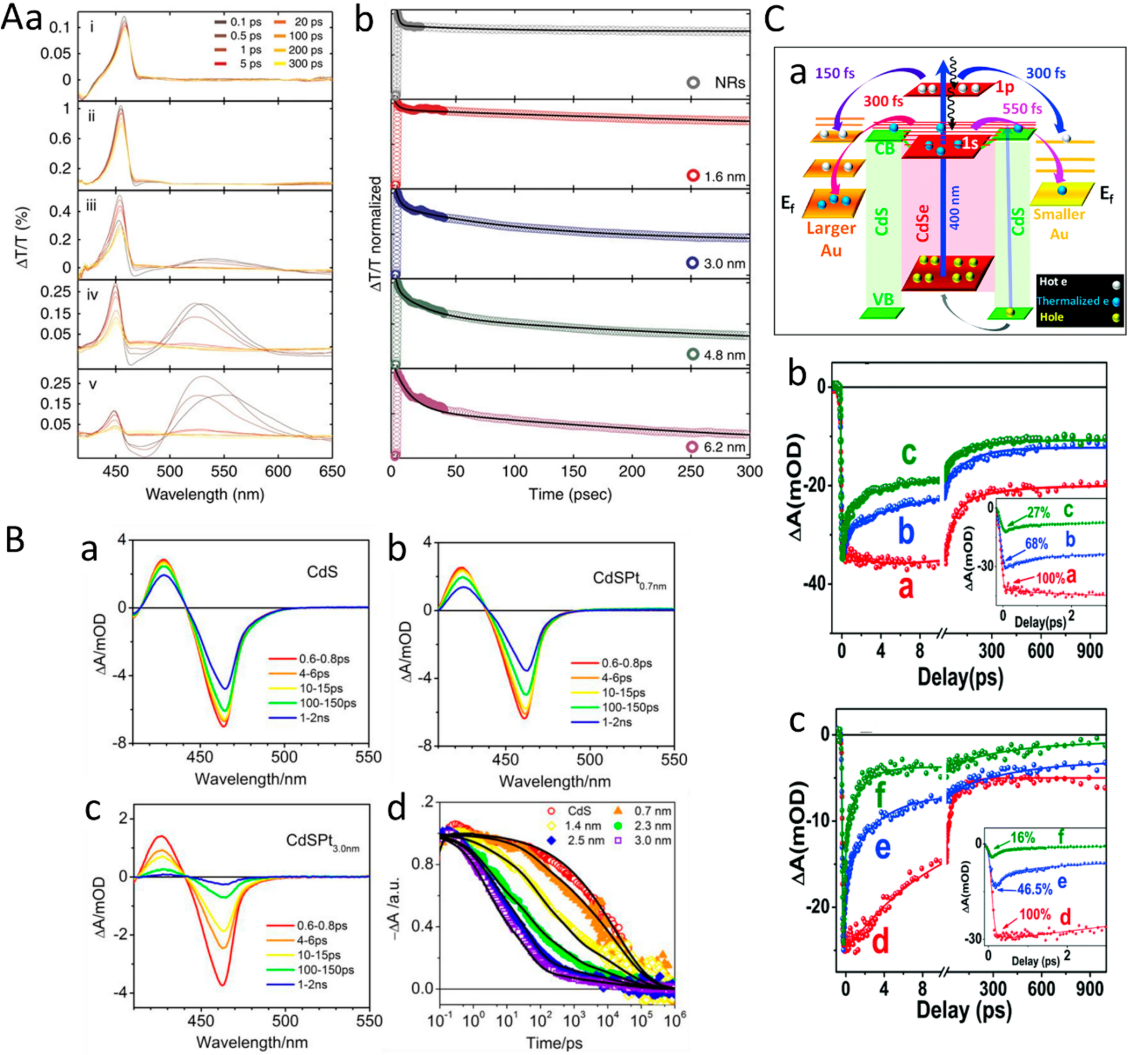
Light-induced charge separation—metal domain size dependence. (A) (a) TA spectra of CdS NRs (i) and CdS–Au hybrid nanoparticles for different Au metal tip sizes including 1.6 (ii), 3.0 (iii), 4.8 (iv), and 6.2 nm (v) at 450 nm excitation. (b) Corresponding normalized TA dynamics of the bleach recovery at 450 nm, attributed to the first excitonic transition of the CdS NR component for CdS NRs and CdS–Au hybrid nanoparticles with different Au metal tip sizes. (B) TA spectral evolution of (a) CdS, (b) CdS–Pt 0.7 nm, and (c) CdS–Pt 3.0 nm at indicated delay times after 400 nm excitation. (d) Comparison of the normalized 1Σ exciton bleach recovery kinetics of CdS and 5 CdS–Pt HNP samples (colored symbols) and their fits (solid lines). Faster bleach recovery at larger average Pt sizes is attributed to increased ET rates. (C) (a) Photoexcited hot and thermalized electron-transfer processes in CdSe–CdS–Au HNPs with different sizes of Au NPs, showing shorter time constants (150 and 300 fs) that represent the faster hot and thermalized electron transfer from core–shell NCs to the larger size Au NP as compared to smaller Au NP (300 and 550 fs). (b) Normalized bleach recovery kinetics at the 1S excitonic wavelength for the CdSe/CdS core–shell NCs (red), CdSe/CdS–Au (1.25 nm Au diameter) (blue), and CdSe/CdS–Au (2.5 nm Au diameter) (green) after 400 nm laser excitation. (Inset) Un-normalized bleach recovery kinetics at a shorter time scale at the same concentration to monitor the change in intensity after coupling with Au NPs with different sizes. (c) Normalized bleach recovery kinetics at the 1P excitonic wavelength for the CdSe/CdS core–shell NCs (red), CdSe/CdS–Au (1.25 nm Au diameter) (blue), and CdSe/CdS–Au (2.5 nm Au diameter) (green) after 400 nm laser excitation. (Inset) Un-normalized bleach recovery kinetics at a shorter time scale at the same concentration to monitor the change in intensity after coupling with Au NPs with different sizes. (A) Adapted with permission from ref (28). Copyright 2016 Nature Publishing Group. (B) Adapted with permission from ref (116). Copyright 2022 American Chemical Society. (C) Adapted with permission from ref (205). Copyright 2017 Royal Society of Chemistry.

Another key parameter that allows one to control the charge-separation efficiency and dynamics is the size of both HNP components, the metallic segment and the semiconductor light absorber. TA measurements of matchstick-like hybrid structures such as CdS–Au^28^ and CdS–Pt^116^ with different metal size domains between 1.5 and 6.2 nm and 0.7 and 3 nm, respectively, showed monotonic increased electron charge-transfer rates with increased metal tip sizes (Figure 25A and 25B). This behavior was explained by consideration of the increased density of states of the metal component with larger size using Fermi’s golden rule, which hold steep dependence of the density of states on the tip volume (*r*^3^), along with weaker contribution through the dependence of the Fermi energy and work function on the metal domain radius (*r*).

Kinetic measurements of charge separation in CdSe/CdS–Au HNPs with different Au size decoration showed that increasing the Au NP size from 1.25 to 2.5 nm caused the transfer time of hot electrons to decrease from 300 to 150 fs (Figure 25C).^205^ The charge-transfer rates of thermalized electrons from the conduction band to the free energy level at the metal domain were measured as 300 and 550 fs for large and small Au NPs, respectively, consistent with the previous reports (Figure 25C-b). Interestingly, the charge-separation dynamics of CdSe/CdS–Ni NRs with different metal domain sizes (2.3–10.1 nm) was found to be independent of the metal tip sizes.^117^ Noteworthy, the authors reported the nonmonotonic behavior of the pre-exponential factor of the TA measurements multiexponential fit which is indicative of the relative weight of rod intrinsic relaxation versus charge-separation processes.

**Figure 26:**
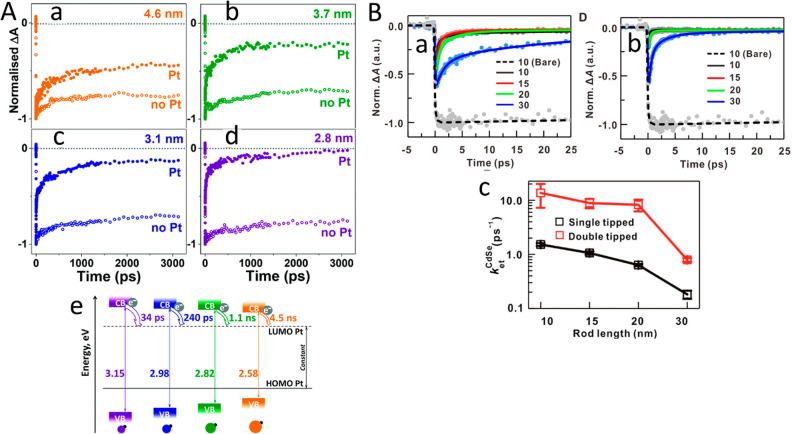
Light-induced charge separation—semiconductor component size dependence. (A) (a–d) TA bleach recovery following excitation of CdS NCs with four sizes without and with decorated Pt. (e) Schematic representation of the electron-transfer rate related to the quantum confinement-induced energetic offset between the electronic states of the semiconductor with respect to the cocatalyst. (B) TA bleach recovery of the (a) single- and (b) double-tipped NRs in the picosecond range. Rod lengths are given in nanometers. (c) Electron-transfer rate (*k*_et_^CdSe^) of single-tipped (black) and double-tipped nanorods (red). (A) Adapted with permission from ref (206). Copyright 2018 Royal Society of Chemistry. (B) Adapted with permission from ref (207). Copyright 2021 American Chemical Society.

The size of the semiconductor component was also shown to influence the charge carrier dynamics. Jackel and co-workers reported on a variation of over 2 orders of magnitude in the transfer rates of photogenerated electrons in different sizes of spherical CdS–Pt HNPs in the range of 2.8–4.6 nm in particle diameter. In small NPs (2.8 nm), charge transfer to Pt sites has an average time scale of ∼30 ps, giving a transfer rate of 2.9 × 10^10^ s^–1^, while in significantly larger particles (4.8 nm), the transfer rates decrease to 1.4 × 10^8^ s^–1^ (average lifetime of 4500 ps) (Figure 26A).^206^ This size-dependent electron-transfer rate is attributed to the tunable electron-transfer driving force, which is the result of the size-tunable band gap of the nanocrystals due to quantum confinement effects of the semiconductor NPs. Since the conduction band edge shows an increasing energy offset with smaller NP size relative to the Pt sites, a larger driving force for electron transfer in the smaller nanocrystals is gained (Figure 26A-e). A different aspect of the semiconductor size was observed in Pt-tipped CdSe NRs (both single and doubled tips) with different rod lengths. In this case, the rate constants for the electron transfer decrease with the rod lengths (Figure 26B).^207^ The origin of this trend, according to the authors, is a diffusion-controlled regime in the one-dimensional nanorod system. The comparable transfer rates for 30 nm double-tipped NRs with 15 and 20 nm single-tipped NRs support this explanation (Figure 26B-c).

**Figure 27:**
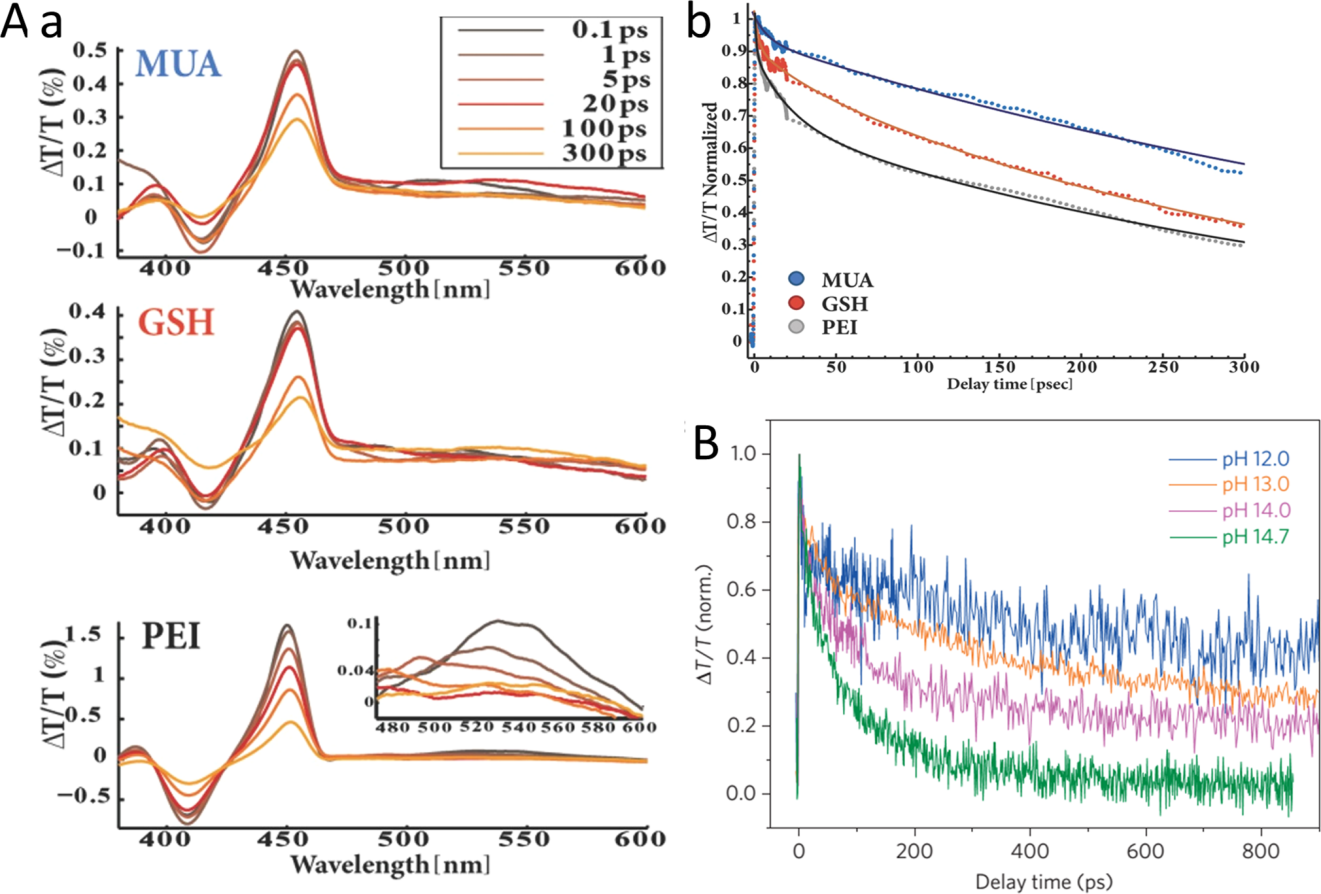
Light-induced charge separation—surface and hole removal effects. (A) (a) TA spectra of CdS–Au HNPs for different surface coatings at 450 nm excitation. Expansion of the Au plasmon region for the PEI coating is presented in the inset (lower left frame). (b) Normalized TA of the bleach recovery at 460 nm, corresponding to the first excitonic transition of the CdS NRs component, along with exponential fits (continuous lines) for CdS–Au HNPs with different surface coatings. Blue color, MUA coating; red, GSH; black, PEI. (B) TA traces taken for CdS–Ni HNPs show an accelerated decay of the ground-state bleach at high pH. (A) Adapted with permission from ref (32). Copyright 2015 Wiley. (B) Adapted with permission from ref (71). Copyright 2014 Nature Publishing Group.

Surface ligands have long been recognized for their numerous roles and functionalities dictating the characteristics of semiconductor NPs and HNPs. Light-induced charge separation was also studied in this context. Comparison between the TA dynamics of CdS–Au NRs with different surface coatings reveals two substantial and related differences, as can be seen in Figure 27A.^32^ First, the fastest charge-transfer dynamics is seen in the case of the polyethylenimine (PEI)-coated HNPs, slower with l-glutathione (GSH), and slowest with mercaptoundecanoic acid (MUA)-passivated HNPs with measured half-lives of 100 ps for PEI and 160 and 330 ps for GSH and MUA, respectively. A related second difference is that the decay amplitude over the measurement time scale in these experiments is also largest in the PEI-coated system. The trend of decay dynamics is attributed to an improved surface passivation leading to lower electron–hole trapping which promoted enhanced electron transfer. Moreover, this electron–hole trapping due to Coulombic interactions in the vicinity of multiple Pt sites deposited on CdS NRs promoted faster charge-transfer rates (0.4 ns) in comparison to the same HNPs in the presence of hole scavengers (8 ns).^198^ Extracting the holes eliminates the Coulomb interactions, which leads to the loss of the electron wave function localization near the hole trap sites and therefore the transfer is delayed. Conversely, fast removal of holes populations in CdS–Ni HNPs by introducing high alkaline environment conditions resulted in faster electron transfer to the metal sites due to reduced Coulomb interactions which allowed increased overlap of the electron wave function with increased growth of the metal sites (Figure 27B).^71^

**Figure 28:**
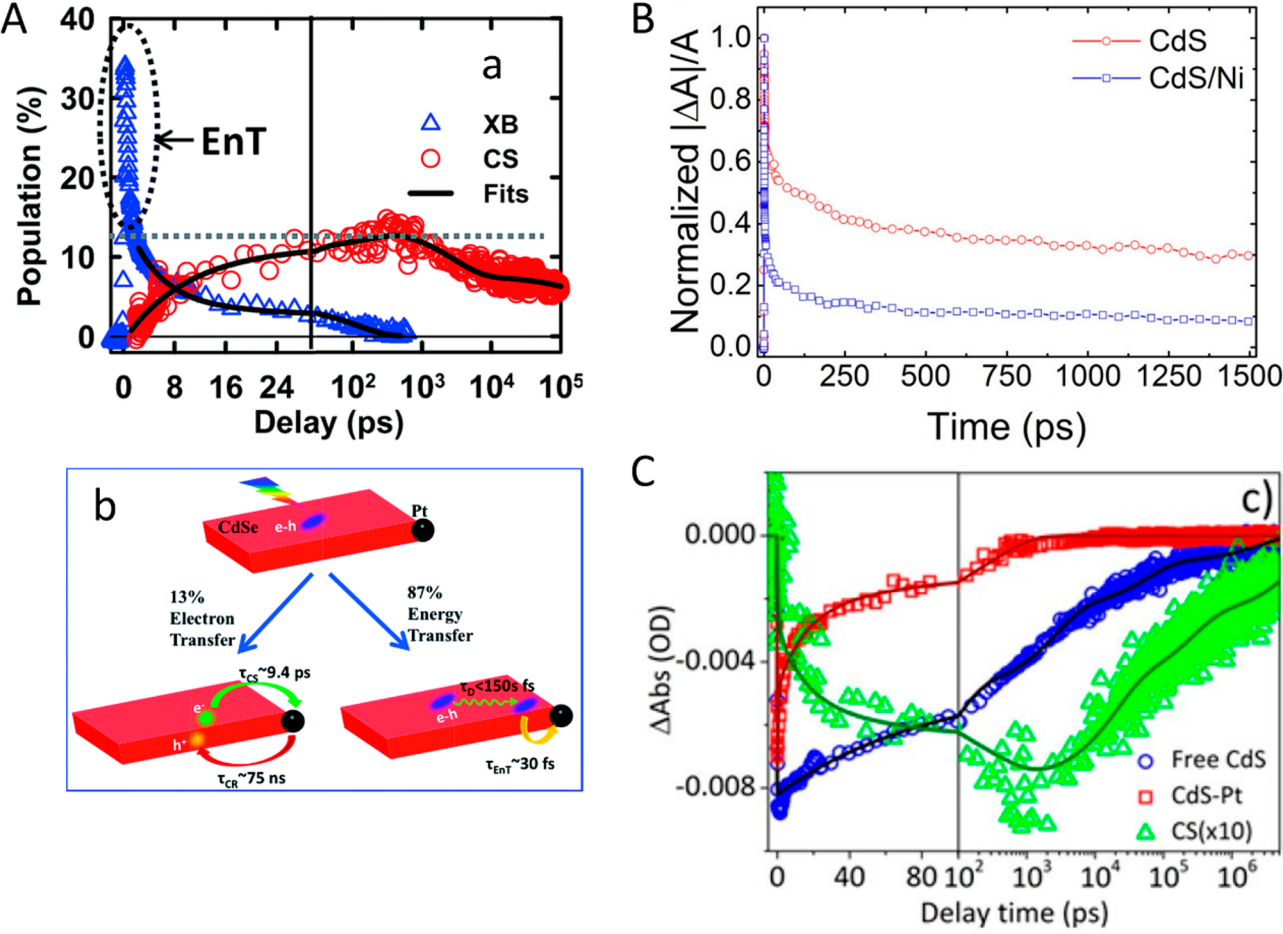
Light-induced charge separation—energy and electron transfer in hybrid NPLs. (A) (a) Time-dependent population of exciton bleach (XB, blue triangles) and charge-separated states (CS, red circles). Black solid lines are multiexponential fits to them. Gray dashed line indicates the efficiency of exciton dissociation (13.4 ± 0.5%). Gray dashed circle indicates ultrafast exciton quenching by energy-transfer pathways. Delay time axis is in linear scale at early delay times and in logarithmic scale at longer decay times. (b) Competition between energy- and electron-transfer pathways in CdSe–Pt hybrid NPLs. Fast in-plane exciton mobility leads to fast energy-transfer quenching. Trapped excitons can be dissociated by electron transfer to Pt. (B) Band-edge TA bleach kinetics for CdS and CdS–Ni hybrid NPLs. (C) Comparison of exciton bleach (XB) kinetics at 416 nm of CdS NPLs (blue circles) and CdS–Pt hybrid NPLs (red squares), and scaled charge-separated-state (CS) kinetics (green triangles) along with their fits shown in solid lines. (A) Adapted with permission from ref (181). Copyright 2015 Royal Society of Chemistry. (B) Adapted with permission from ref (74). Copyright 2015 American Chemical Society. (C) Adapted with permission from ref (61). Copyright 2018 American Chemical Society.

Structural characteristics of HNPs such as their components morphology can affect the excited charge carrier’s relaxation mechanism as well as their dynamics. Two-dimensional HNPs (NPLs) compared to 0D and 1D HNPs (dots and NRs, respectively) are expected to promote faster charge transfer and a longer charge-separation distance due to more uniform quantum confinement and a large surface area, respectively.^193^ Interestingly, while CdS–Pt NRs exhibit excitons relaxation solely through charge separation by charge transfer of electrons to the metal domain, in the case of CdSe–Pt NPLs, exciton quenching takes place mostly via fast diffusion to the semiconductor–metal interface followed by rapid energy transfer to Pt (∼87%). Only a small fraction of the excitons (∼13%) can undergo charge separation (Figure 28A).^181^ The key differences between CdSe NPLs and CdS NRs are that in the latter, exciton quenching through diffusion and energy transfer is slow compared to hole trapping, leading to a near unity yield of charge separation in their hybrid form. This difference can be attributed to an atomically precise thickness in the quantum-confined dimension in NPLs, which minimizes energy disorder and enables ultrafast exciton diffusion. However, a charge carriers relaxation study on CdS–Ni NPLs indicates excitons quenching via a charge-transfer pathway with a time constant of ∼165 ps (Figure 28B).^74^ This difference can be elucidated by the presence of a greater number of trap states in CdS in comparison to CdSe NPLs.^208^ This may prevent ultrafast energy transfer and facilitates charge separation due to exciton trapping. This hypothesis was further supported by charge carrier dynamic measurements of CdS–Pt NPLs showing the growth of the charge-separation signal along with the recovery of the bleaching TA signal (Figure 28C).^61^

#### 3.3.2 Plasmon-Induced Charge Separation

**Figure 29:**
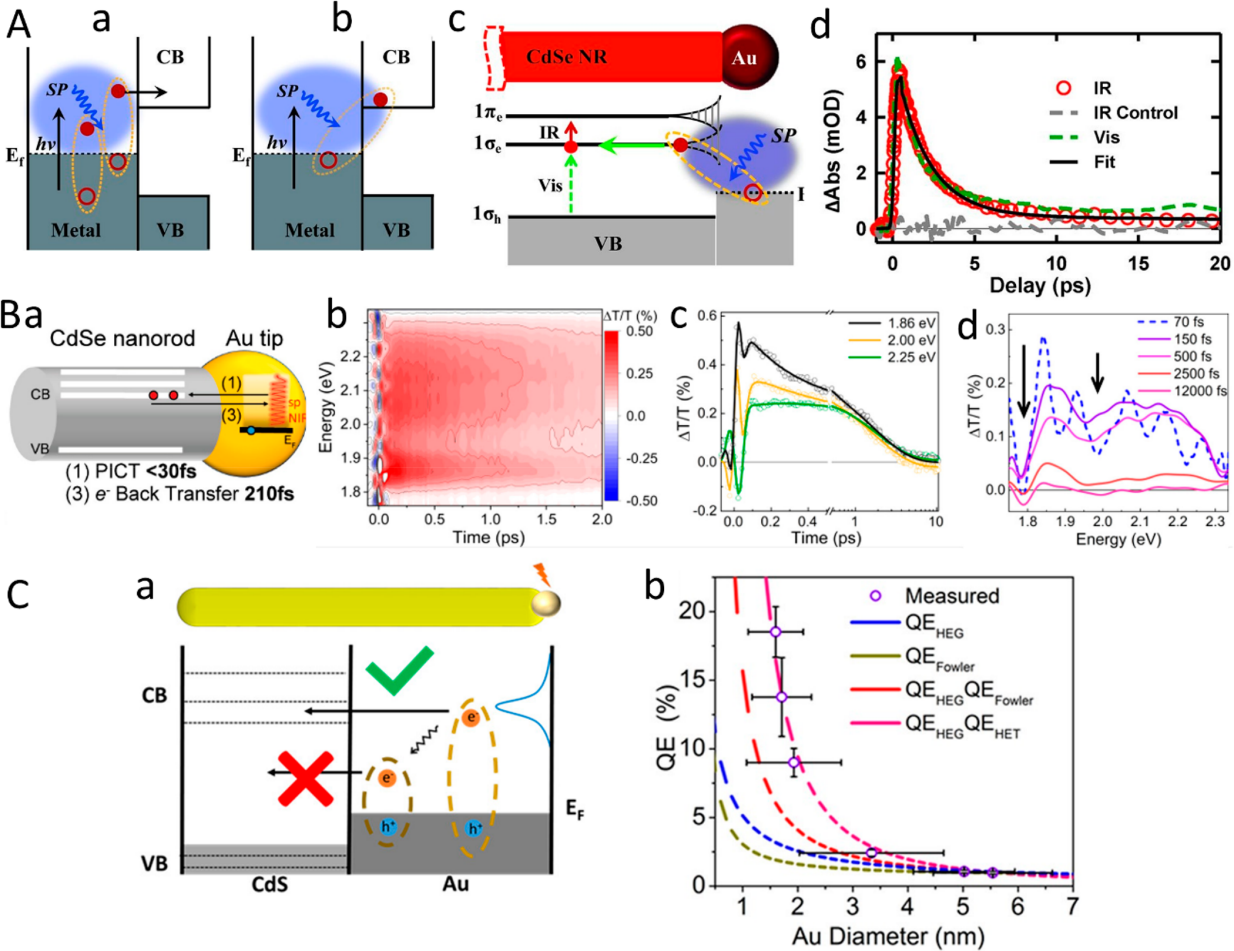
Plasmon-induced charge separation. (A) (a) Conventional PHET mechanism in which a photoexcited plasmon (SP, blue ellipsoid) in the metal decays into a hot-electron–hole pair (solid and open red circles in the dotted ellipsoids) through Landau damping, followed by injection of the hot electron into the CB of the semiconductor. Electron–hole pair has a broad distribution of initial electron and hole energies; only two are shown for clarity. (b) PICTT pathway, where the plasmon decays by directly creating an electron in the CB of the semiconductor and a hole in the metal. VB is the semiconductor valence band, and *hv* indicates the excitation photons. (c) Schematic electronic structure of a CdSe–Au nanorod composed of a strongly damped Au tip with broadened electronic levels and a central region with relatively unperturbed discrete levels (1σ_e_, 1π_e_, 1σ_h_). Green dashed arrow indicates the interband transition in the visible (Vis) spectrum, red arrow indicates the intraband transition in the IR spectrum, and green solid arrow indicates electron transport in the nanorods (d) Intraband absorption (red circles) and 1S-exciton-bleach (green dashed line) kinetics of CdSe–Au NRs after 800 nm excitation. Negligible intraband absorption signal is apparent in a control sample of a mixture of CdSe NRs and Au nanoparticles (gray dashed line). Black solid line is a multiexponential fit of the kinetics. (B) (a) Photophysics of CdSe–Au HNPs following NIR excitation; PICT generates electrons in the CdSe domain with a ≤30 fs time constant. This is followed by a back-transfer to the gold in 210 fs. (b) TA map of CdSe–Au HNPs using a near-infrared pulse centered at 1.3 eV as a pump (fluence of 135 μJ/cm^2^) and the visible one as a probe. (c) TA kinetics at selected photon energies (dots), and fits to the data (lines). (d) TA spectra at selected time delays. (C) (a) Schematic of sequential plasmon decay and hot carrier transfer at the Au nanoparticle/CdS nanorod interface. (b) Measured Au to CdS PHET quantum efficiency as a function of Au sizes (violet circles) and its fit by functions that account for different mechanisms, revealing the best fit to a combination of enhanced hot-electron generation and transfer efficiencies models. (A) Adapted with permission from ref (148). Copyright 2015 AAAS. (B) Adapted with permission from ref (149). Copyright 2021 American Chemical Society. (C) Adapted with permission from ref (213). Copyright 2020 American Chemical Society.

Indeed, the majority of the charge-separation studies in HNPs have been focused on the photoinduced charge transfer from the semiconductor to the metal domain. However, combination of the unique properties of an excited plasmonic material, such as enhanced local fields near the metal nanostructures, broad spectral tunability, large absorption cross sections, and superior long-term stability, with excitonic sensitization through the semiconductor has interesting potential for SPR-driven hot-electron photochemistry applications. This promising route motivated the investigations of plasmon-induced hot charge carrier transfer across the interface of the heterostructured system. Within this process, hot carriers generated from plasmon decay can transfer into a nearby semiconductor through two mechanisms as illustrated in Figure 29: the conventional indirect plasmon-induced hot-electron transfer (PHET)^29,152,209,210^ and the recently discovered direct plasmon-induced charge transfer (PICT).^148,211,212^ In PHET, hot carriers are initially generated in the metal by plasmon decay and then undergo interfacial transfer to the acceptor component. PICT occurs when there is strong interdomain coupling and mixing of the metal and acceptor energy levels. In PICT, plasmon excitation is directly accompanied by a rapid charge-separation process that creates an electron in the acceptor region and a hole in the metal.

Indication of the PICT mechanism was demonstrated by TA measurements of the CdSe–Au NRs interband transition and mid-IR intraband absorption which showed corresponding decay rates following near-IR excitation at the metal plasmon absorption region, indicating the formation of semiconductor conduction band electrons (Figure 29A-d). The estimated plasmon-induced hot-electron-transfer and charge-recombination times were found to be ∼20 fs and ∼1.45 ps, respectively, with a quantum efficiency that exceeds 24%.^148^ By applying ultrafast spectroscopy with high temporal resolution on similar CdSe–Au NRs, an upper limit of 30 fs for electron transfer could be determined, and further fast back transfer of electrons to the metal domain within 200 fs time scale was observed (Figure 29B).^149^ Hence, for exploiting the electrons generated in the semiconductor via PICT, their extraction (by surface chemistry reactions) needs to be extremely rapid to compete with the fast back-transfer to the metal. In addition, complementary measurements of the charge transfer to the metal following visible-light excitation allowed one to present the complete relaxation pathways of excited charge carriers in this nanosystem, signifying that the PICT mechanism mostly generates band-gap electrons in CdSe since no hot-electron back-transfer to the gold at the relevant time scale was observed. Nevertheless, in the case of CdS–Au NRs, the Au plasmon band is weakly perturbed and the plasmon-induced hot-electron transfer occurs through the conventional PHET mechanism. Hence, the hot-electron-transfer rate is noticeably slower (97 fs) due to the rapid plasmon decay and the efficiency is reduced (∼2.75%) owing to the competition of hot-electron transfer with ultrafast relaxation.^29^ However, Liu et al. presented an improved quantum efficiency of hot-electron transfer in CdS–Au NRs via control over the metal domain sizes. The quantum efficiency of this process increases from ∼1% to ∼18% as the particle size decreases from 5.5 to 1.6 nm (Figure 29C).^213^ This trend is attributed to a dual effect of enhanced surface damping contribution and decreased barrier height and possibly enhanced electronic coupling at the semiconductor–metal interface.

The effect of other structural parameters on the hot-electron transfer from the metal site to the semiconductor components was investigated. Weng and co-workers recently studied the effect of the semiconductor shell thickness in Au–CdS core/shell HNPs with different thicknesses between 3.6 and 14 nm. A nonmonotonic trend of hot-electron-transfer efficiency was observed with maximum efficiency at 8.2 nm shell thickness.^142^ This trend was assigned to two opposing factors: the initial hot-electron energy distribution in the Au core and the Schottky potential at the interface of the Au core and the CdS shell. Both factors are reduced with increased shell thickness, and therefore, an intermediate shell thickness can balance these trends. Energy band alignment was also reported to influence the electron–phonon relaxation processes. Varying the size and type of the semiconductor segment in semiconductor–metal dimers resulted with an acceleration of the electron–phonon scattering rate for HNPs owing to the well-aligned semiconductor conduction band and metal Fermi level.^214^

#### 3.3.3 Charge Separation in Multiexciton Regime

**Figure 30:**
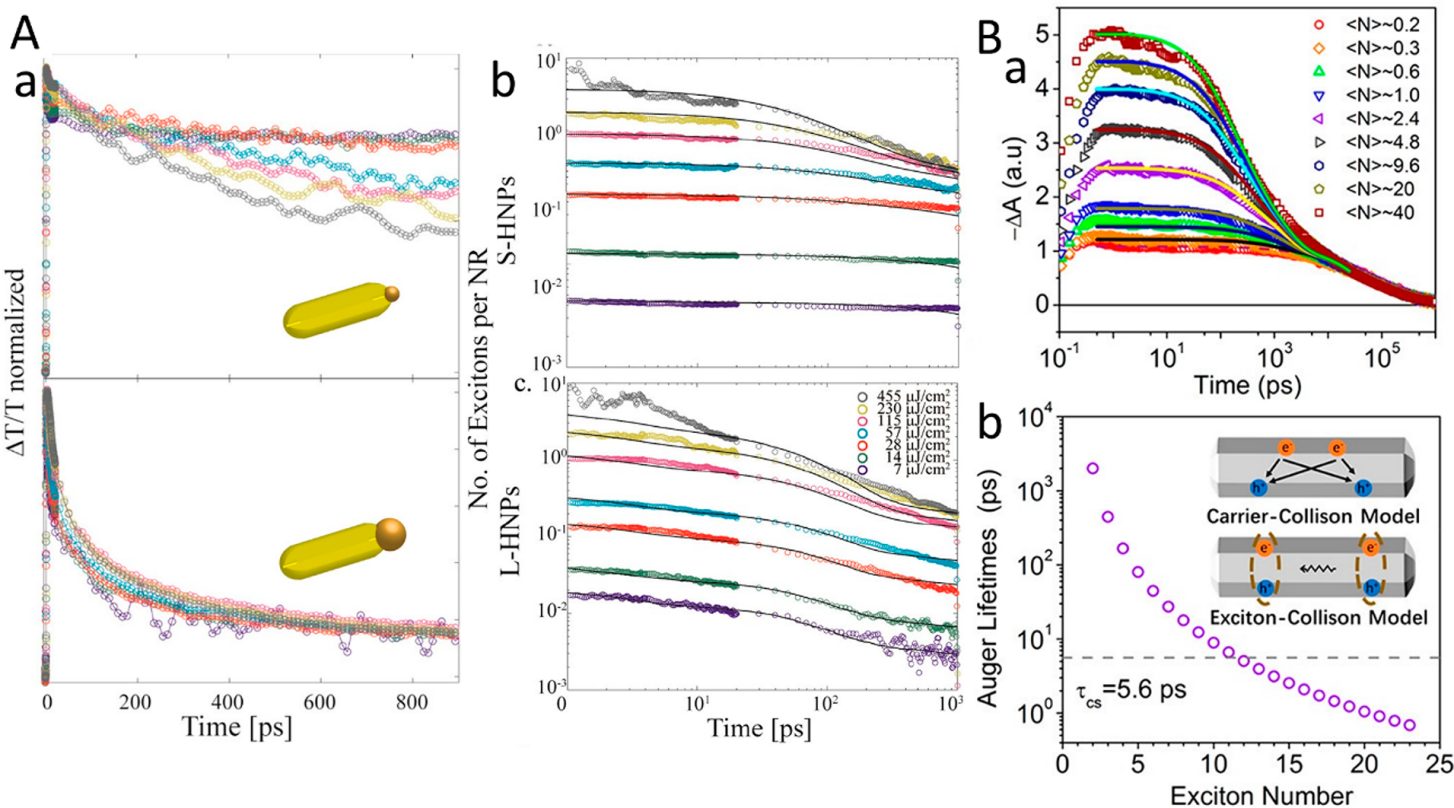
Charge separation in multiexciton regime. (A) (a) Normalized dynamics of the bleach recovery at 450 nm at different fluences, attributed to the first excitonic transition of the CdS NR component for CdS–Au hybrid nanoparticles with different Au metal tip sizes. (b) Experimental dynamics of the number of excitons per rod for small and large metal-tipped CdS–Au HNPs (upper and lower panels, respectively) at different fluences, along with the fitted Markov chain Monte Carlo simulation curves (solid black lines). (B) (a) Comparison of normalized 1Σ exciton bleach recovery kinetics measured under various fluences (⟨*N*⟩ ≈ 0.2–40) and their fit according to the carrier-collision model. This bleach kinetics has been normalized to the same amplitude at 100 ns and shows excellent agreement from 50 ns to 1 μs when only single exciton states remain in these NRs. (b) Auger lifetime of N exciton states in CdS NRs obtained through the fit shown in a according to the carrier-collision model (purple open circles) and charge-separation half-lifetime of a single exciton state in CdS–Pt (gray dashed line). (Inset) Scheme of the carrier-collision (top) and exciton-collision (bottom) models for multiexciton Auger recombination. (A) Adapted with permission from ref (218). Copyright 2018 American Chemical Society. (B) Adapted with permission from ref (219). Copyright 2021 American Chemical Society.

Most of the studies on excited charge carrier dynamics in general and specifically on charge separations have focused on a single exciton excitation regime. As was described in the previous sections, a single electron–hole pair is generated following light excitation. The excited-state relaxation routes and its dynamics were extensively reviewed in this manuscript and others. The ability of semiconductor NPs to generate and accommodate multiple excitons (MXs) through optical or electrical stimulation holds potential in utilizing HNPs in various applications that require multielectron reactions such as water splitting and CO_2_ reduction. Typically, MXs in a semiconductor nanocrystal are generated by the absorption of multiple photons through high-intensity irradiation.^31,215^ Generation via multiexciton generation processes in which the absorption of one high-energy photon creates two or more lower energy excitons was also demonstrated and studied.^216,217^ MX decay dynamics in semiconductor NCs is governed by Auger recombination in which an exciton recombines nonradiatively and transfers its energy to another charge carrier which then rapidly dissipates it, resulting in the effective annihilation of one exciton. Interestingly, in HNPs, the multiexciton state can also dissipate through charge transfer from the semiconductor to the metal domain. Ben-Shahar et al. reported on a metal domain size effect in CdS–Au NRs governing MX dissociation. It was shown that large metal domains which promote faster charge-transfer rates in comparison to small Au-tipped NRs (45 ps vs 5 ns) can outcompete the Auger recombination rate (180 ps), while the relaxation process of semiconductor NRs and small Au-tipped NRs is dominate by Auger recombination (Figure 30A).^218^

This competitive process can be influenced also by the material combination of HNPs. CdS–Pt NRs were reported to present slow Auger recombination rates due to trapped holes and spatially separated excitons varying between 2 ns and <1 ps for biexcitons and multiexcitons (>20 excitons), respectively (Figure 30B).^219^ Hence, under the MX state, the efficiency of multiple electron transfer to the metal site was set to ∼41% compared to 100% of the MX dissociation efficiency calculated for the biextonic state. Note that the differences between these two nanosystems can also originate from the different stabilizing ligands and solvent environment. Additionally, heterostructured semiconductor–metal HNPs such as CdSe/CdS–Pt NRs reveal fluence-dependent band-edge exciton dissociation. In this case, the first electron-transfer rate was measured with a time constant of 192 ps, while the second electron transfer is slowed by the trion Coulombic interaction down to 1700 ps.^220^ The Coulomb barrier that arises following trion formation was estimated to increase by ∼60 meV, causing a slower transfer rate, and the transfer efficiency decreased by 1 order of magnitude.

### 3.4 Photocatalytic Properties

In addition to the former described properties that involve intraparticle characteristics, the photocatalytic ability of such semiconductor–metal HNPs stems from interparticle processes. The advantages of enhanced optical properties along with the synergistic light-induced charge separation form the basis for this unique feature. The efficient charge separation across the semiconductor–metal interface allowed the utilization of these charge carriers, electrons and holes, at the surface of each of the hybrid components for additional surface chemistry reactions.

**Scheme 5:**
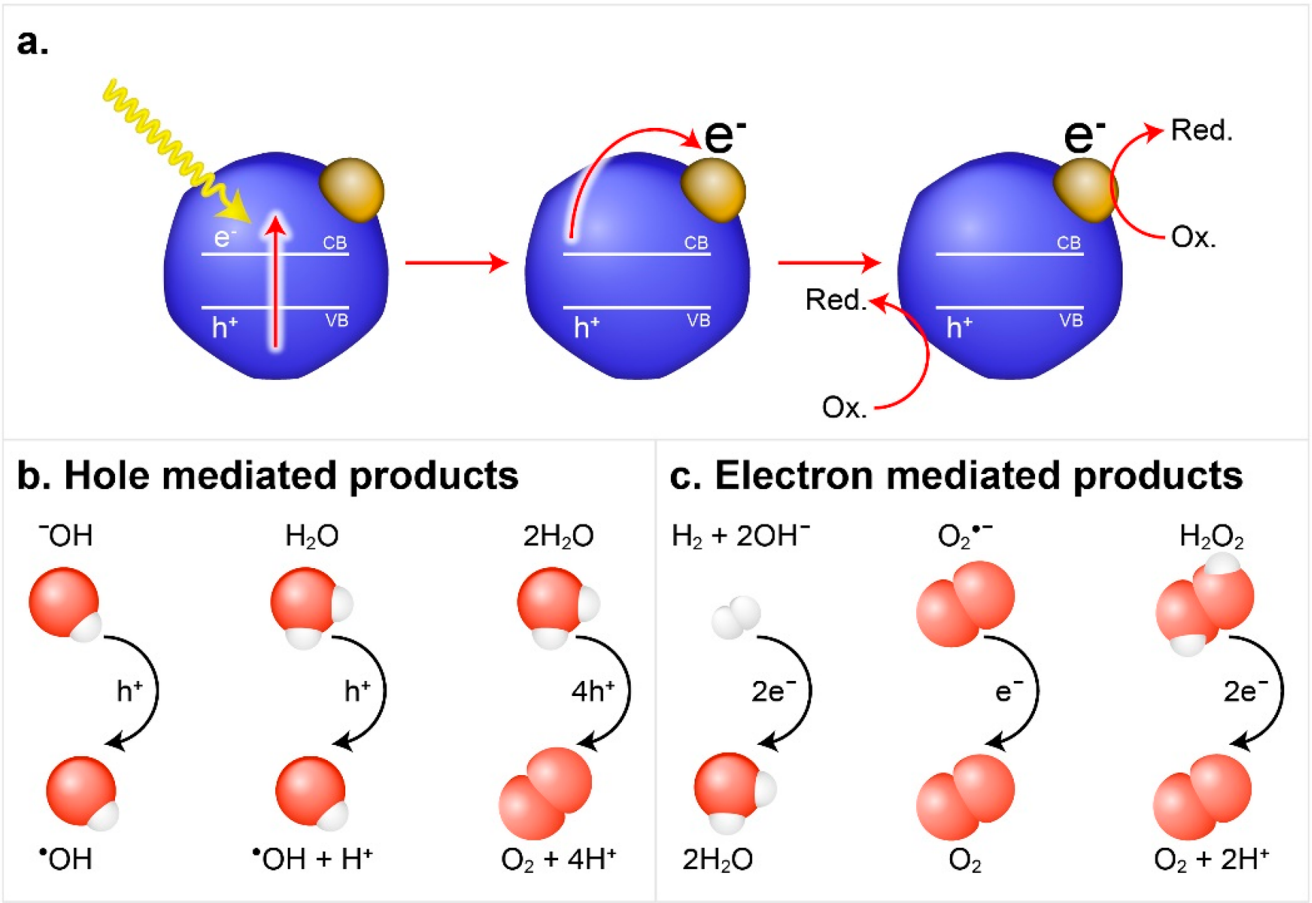
(a) Schematic of the Photocatalytic Process and Products of a Semiconductor–Metal Hybrid Nanoparticle, (b) Hole-Mediated Oxidized Entities, and (c) Electron-Mediated Reduced Entities

Typically, the majority of such chemistry are redox reactions. Scheme 5 illustrates the available reduction and oxidation routes following light absorption by HNPs. For the excited electrons, typically transferred to the metal component, water reduction to hydrogen is possible, while under aerobic conditions, the electrons may reduce molecular oxygen to form hydrogen peroxide and/or superoxide radicals. Simultaneously, the excited holes, in some of the illustrated scenarios, are capable of oxidizing water molecules to form hydroxyl radicals. All of these routes have been utilized and demonstrated in various applicable fields including alternative energy generation, biomedical, environmental, and industrial applications. These will be discussed in detail in the next sections.

**Figure 31:**
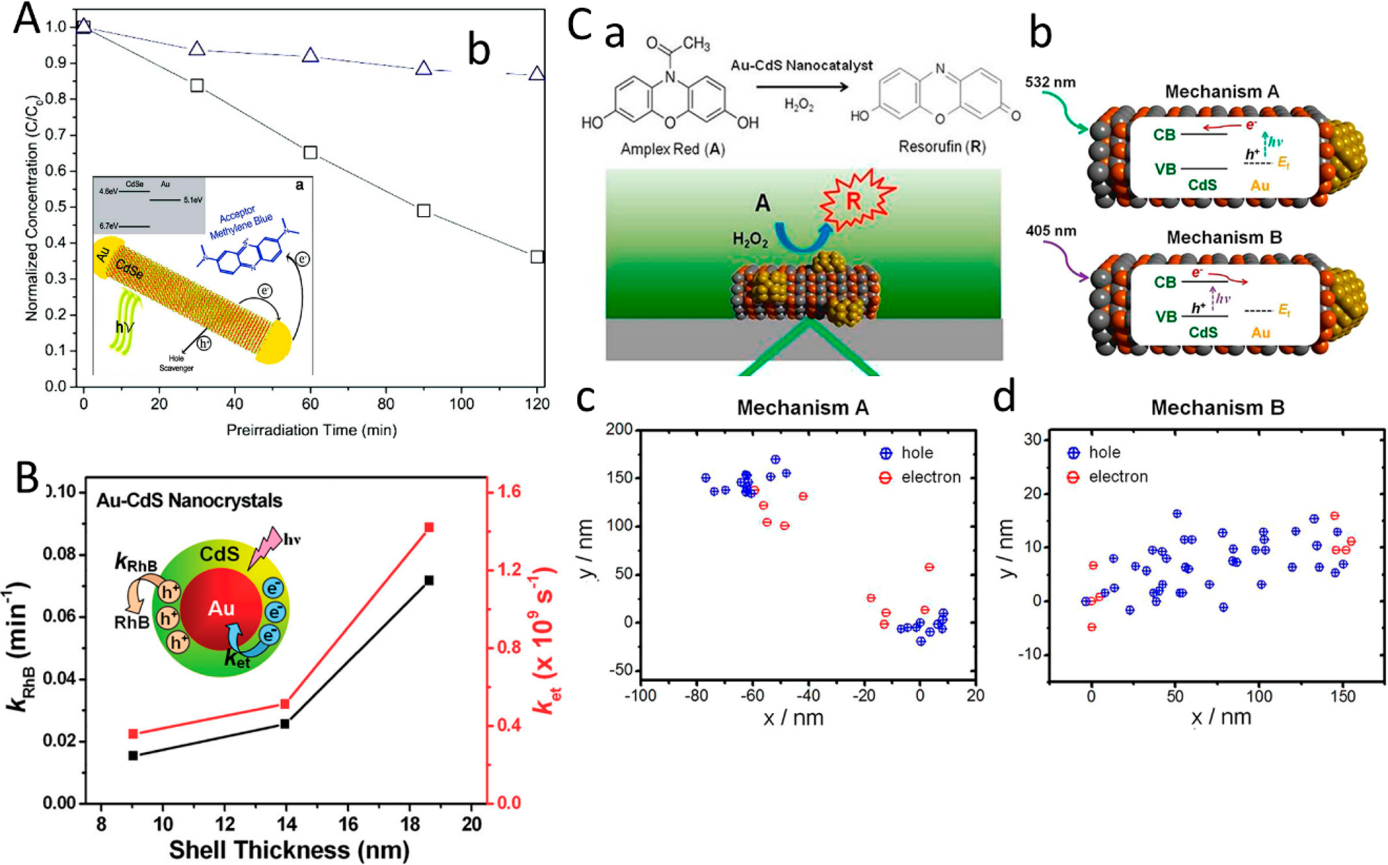
Photocatalytic properties of HNPs. (A) (a) Normalized concentration of MB dye reduced by a CdSe nanorods–gold nanoparticles mixture (open blue triangles) and hybrid CdSe–Au nanodumbbells solution (open black squares) vs preirradiation time. High efficiency of the charge retention in NDBs is demonstrated, leading to activity toward MB reduction. (Inset) Schematic of a light-induced charge-separation mechanism in a nanodumbbell. Scheme depicts the transfer of the hole to the scavenger and the reduction of the MB molecule upon electron transfer from the gold tip. (Inset) Energy band alignment between CdSe and Au. (B) Correlations of electron-transfer rate constant (*k*_et_) and rate constant of RhB photodegradation (*k*_RhB_) with the shell thickness of Au–CdS nanocrystals. (C) (a) Experimental scheme using total internal reflection fluorescence microscopy to probe the fluorogenic oxidation reaction of nonfluorescent Amplex red (A) to highly fluorescent resorufin (R) by a CdS–Au HNP. (b) Schematic illustrating two distinct photocatalysis mechanisms with the opposite direction of energy flow (opposite polarity after photoinduced charge separation). In mechanism A at 532 nm, the photogenerated energetic electrons in the gold metal are injected to the conduction band (CB) of the semiconductor. In mechanism B at 405 nm, the photogenerated electrons in the CB of the semiconductor are rapidly trapped by the gold metal. (c) Super-resolution mapping of single reactive sites on an Au-tipped CdS nanorod heterostructure during the oxidation reaction at 532 nm (mechanism A). (d) Super-resolution mapping of single reactive sites on an Au-tipped CdS nanorod heterostructure during the same oxidation reaction after turning on the 405 nm laser (in addition to the 532 nm laser, needed to excite the resorufin product) (mechanism B). (A) Adapted with permission from ref (177). Copyright 2008 American Chemical Society. (B) Adapted with permission from ref (227). Copyright 2010 American Chemical Society. (C) Adapted with permission from ref (221). Copyright 2014 American Chemical Society.

One of the most common methodologies to investigate and demonstrate the photocatalytic characteristics of HNPs is via organic dye photoreduction/oxidation. This photocatalytic process, which also bears relevance for various photocatalytic purification applications, can be mediated by either excited electrons or holes at both the semiconductor and the metal components. In each of the pathways, degradation of the organic dyes is occurring directly by charge carriers at the hybrid surface or through the formation of radicals (mainly in aerobic environments). First demonstration of the photocatalytic performance of HNPs was reported for CdSe–Au nanodumbbells showing efficient methylene blue (MB) reduction (up to 64%) following visible-light excitation via electron reduction of the dye molecules (Figure 31A).^177^ In addition to in situ degradation measurements, charge retention of electrons on the metal tips was observed by using preirradiated experiments. Similar reports of photoinduced dye degradation by numerous different HNPs were reported using various dye molecules including MB, methyl orange (MO), rhodamine B (RhB), and rhodamine 6G (R6G). Cd–chalcogenide-based HNPs such as CdSe–Au NPs,^175^ CdS–Au NRs,^221,222^ CdSe/CdS–Pt NRs,^75,223^ Au–CdS yolk/shell,^224^ CdS–Au nanorings,^225^ CdS–Au,^226^ and CdSe/CdS–Au^42^ NPLs were all used to explore the mechanism of the photocatalytic reaction and the HNPs structural and chemical effects on this specific property. A different mechanism was suggested by some of these reports describing a radical-mediated photocatalysis of dye molecules. Where following light absorption and charge carriers excitation, superoxide and hydroxyl radicals are generated by electron reduction of molecular oxygen and hole oxidation of H_2_O molecules, respectively. Consequently, these radicals can further degrade the dye molecules in their surroundings.

As was described for the other mentioned properties, the photocatalytic property is also influenced by the structural effect of the HNPs. In this context of dye degradation, it was reported that the increased shell thickness of Au–CdS core/shell NPs enhances its photocatalytic activity due to increased absorption by the larger semiconductor component that provides generation of more charge carriers (Figure 31B).^227^ The yolk/shell architecture of Au–CdS was shown to be more efficient in photocatalytic R6G degradation in comparison to core/shell HNPs where R6G was almost completely degraded in the former form while the later achieved 68% bleaching.^224^ The authors attributed this trend to several factors, among them the increase in hole trapping sites that can promote more hydroxyl radicals along with the larger surface area of the yolk structure. Other types of HNPs, such as Au–Bi_2_S_3_,^98^ Au–SnS,^95^ Au–CZTS,^25,36,53^ Pt–CZTS,^25,53^ Pd–CZTS,^53^ and Cu_2–*x*_S–CdS shells on Au NRs^228^ also revealed an improved photocatalytic ability in comparison to their semiconductor NP component form, exemplified via organic dye degradation.

Moreover, this photocatalytic dye transformation has been applied to identify the spatial distribution of the catalytic activity on HNPs. Ha et al. has reported two distinct, incident energy-dependent charge-separation mechanisms that result in opposite energy flows and polarities on a single CdS–Au NR. By using Amplex red dye (nonfluorescent) that turns into resorufin (fluorescent) following light irradiation of HNPs in the presence of H_2_O_2_, the reaction turnover events can be monitored and localized its position by applying super-resolution mapping.^221^ As seen in Figure 31C, under plasmon excitation (532 nm), the holes reactive sites are positioned at the gold tips on both ends of the HNPs while the electron reactive sites are located along the inner length of the CdS nanorods within a distance of a few tens of nanometers from the gold tips. This indicates that excited hot electrons are transferred from the metal domain to the semiconductor component at this energy excitation. However, under excitonic excitation (405 nm), an opposite polarity after photoinduced charge separation has been observed. In this case, the hole’s reactive sites are distributed along the inside length of the CdS NR, while the electron’s reactive sites are located at both ends; this map can be assigned to charge separation originating from excited electron transfer from the semiconductor to the metal. An additional means of probing charge separation and energy transfer across the semiconductor–metal nanojunction was demonstrated by measuring the quenching kinetics of ATTO dyes in the presence of excited CdS–Au NRs.^222^ Similar trends were reported, showing more efficient catalytic performance under excitonic excitation in comparison to plasmon-induced photocatalysis.

## 4 HNPs at Work: Emerging Applications

Utilization of the above-described properties of HNPs is optimally manifested via photocatalytic applications, perhaps the most notable of which are alternative clean solar-to-fuel conversion in the form of hydrogen generation via photocatalytic water splitting and also toward photocatalytic CO_2_ reduction. A complementary photocatalytic application route under aerobic conditions generating radicals and reactive oxygen species (ROS) was also realized in the fields of biomedicine along with environmental and industrial science. The advancement in synthesis control along with the ability to tune the optical and chemical properties of HNPs allows a judicious design of a predetermined HNPs that address the specific physical and chemical requirements of a given photocatalytic reaction.

### 4.1 Clean Energy–Photocatalytic Water Splitting

One of the most challenging photocatalytic reactions in the field of alternative energy harvesting is the water splitting reaction for hydrogen generation.^229−232^ This reaction pathway opens the way for direct conversion of sustainable solar energy to chemical energy stored in chemical bonds of hydrogen as an alternative fuel. Utilization of hydrogen in fuel cells to generate electricity produces back water in a zero emissions cycle.

To perform complete water splitting, the equilibrated Fermi level of the electrons must be at least more negative than the redox potential of H^+^/H_2_ (0 V versus NHE) while the edge of the valence band that accumulate the holes should be more positive than the redox potential of O_2_/H_2_O (1.23 V).^231^ The actual band positions that are needed are in fact larger due to the condition to overcome the overpotential. Honda and Fujishima were the first to show water splitting by a photoelectrochemical reaction in the early 1970s by using a TiO_2_ electrode under UV irradiation and a Pt counter electrode with applied bias to form a closed circuit.^233^ Both wide-gap metal–oxide semiconductors and narrow-gap metal sulfide semiconductors can in principle address the minimal energetic requirements. However, the difference in the band-gap width determines the absorbance region. Hence, in the pursuit for increasing the solar spectral coverage, the use of narrower gap semiconductors which can absorb in the visible region accounting for about one-half of the solar spectrum is more favorable over the wide-gap common oxide semiconductor absorbing the UV range comprising less than 5% of the solar spectrum.^234^

**Figure 32:**
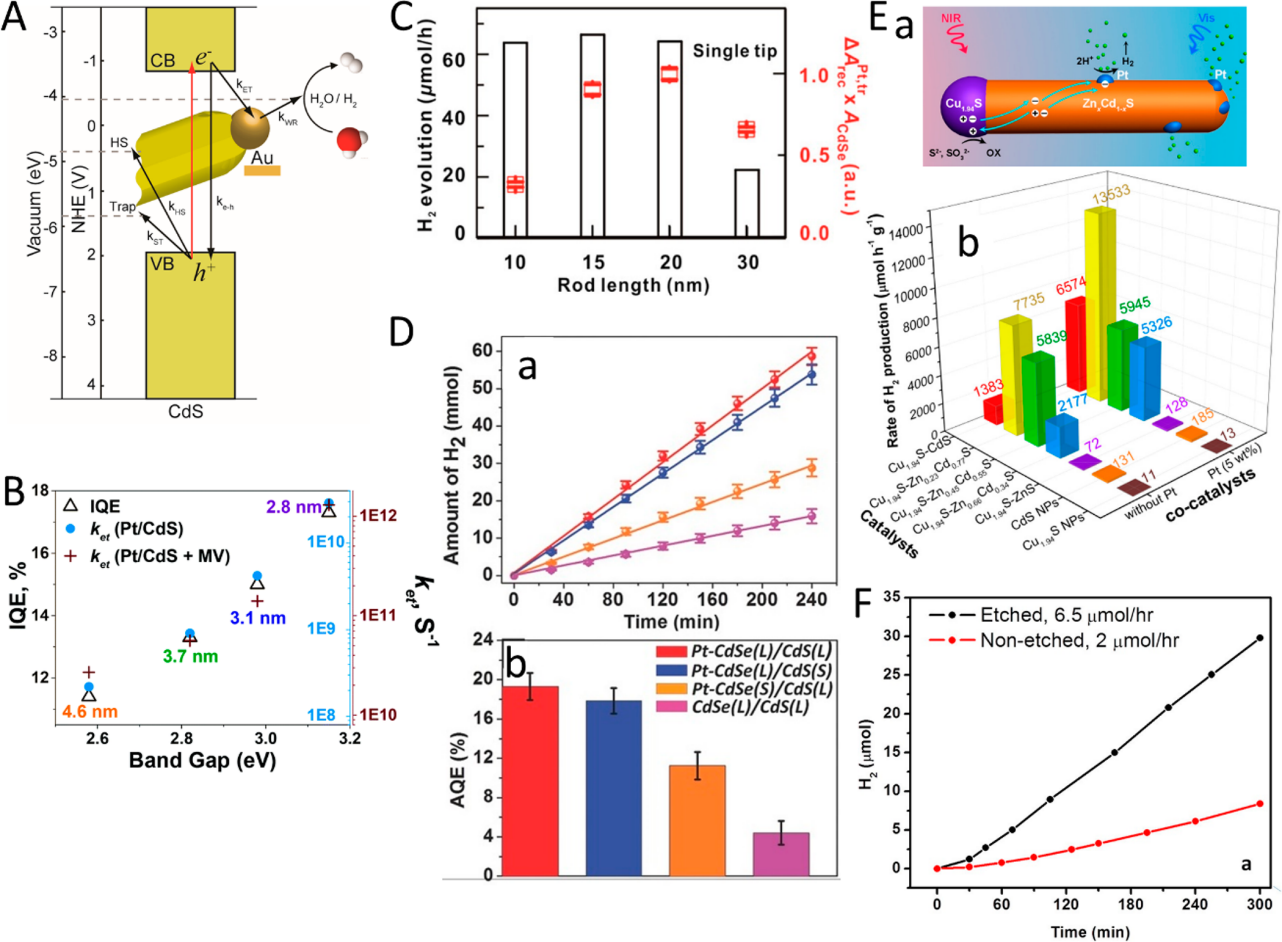
Photocatalytic properties of HNPs. (A) Illustration of photocatalytic hydrogen generation in the presence of sacrificial hole scavengers along with a typical CdS–Au HNPs band alignment diagram with additional recombination and relaxation routes of excited-state charge carriers. (B) Dependence of H_2_ efficiency and electron-transfer rates on the energetic offsets between the CdS NCs conduction band edges and the LUMO position of the Pt clusters. (C) Observed H_2_ evolution rate (black bar) and scaled product of the CdSe absorption and Δ*A*_rec_^Pt,tr^ (%) from the TA measurements (red square) of the single-tipped CdSe NRs. (D) Comparison of photocatalytic activity of CdSe/CdS–Pt core/crown NPLs with different core and crown sizes. (a) Kinetic measurements of total amount of H_2_ generated in the course of reaction and their (b) complementary apparent quantum yields. (E) (a) Schematic of the Pt-decorated Cu_1.94_S–Zn_*x*_Cd_1–*x*_S HNPs. Cu_1.94_S relays the holes from Zn_*x*_Cd_1–*x*_S to the hole scavengers (S^2–^, SO_3_^2–^); electrons are extracted by Pt to transfer rapidly in the single-crystalline 1D structure and eventually diffuse to the surface to carry out water-splitting reactions. (b) Photocatalytic H_2_ production activities of different semiconductor material compositions with and without Pt decoration under visible-light irradiation. (F) Comparison of total amounts of produced H_2_ between etched (black) and nonetched (red) CdSe/CdS–Pt HNPs. (B) Adapted with permission from ref (206). Copyright 2018 Royal Society of Chemistry. (C) Adapted with permission from ref (207). Copyright 2021 American Chemical Society. (D) Adapted with permission from ref (43). Copyright 2017 Wiley. (E) Adapted with permission from ref (132). Copyright 2016 American Chemical Society. (F) Adapted with permission from ref (243). Copyright 2013 American Chemical Society.

HNPs are considered promising photocatalysts for alternative energy harvesting in this form of hydrogen generation.^7,16,88,90,235^ Their advantage over semiconductor NCs related to the ability to tune their photophysical and chemical properties through material combination and dimensions has raised great interest toward their utilization in such application. Yet, only a handful of reports have been able to demonstrate full water splitting HNPs as a single system photocatalyst which can effectively promote both reduction and oxidation of water. Therefore, the use of sacrificial agents such as sulfide/sulfite pair, alcohols, amines, and other electron-donating molecules is commonly imposed where the counter charge carrier cannot be exploited to a complementary redox reaction. Figure 32A presents an illustration of photocatalytic hydrogen generation in the presence of sacrificial hole scavengers along with a typical CdS–Au HNPs band alignment diagram with additional recombination and relaxation routes of excited-state charge carriers.

The significant dependence of the charge carrier’s dynamics on both the HNPs structural properties and the chemical-medium conditions, discussed in section 3.3, was found to directly affect the photocatalytic performance of the HNP nanosystems. These influencing factors, which are critical for progressing toward a viable water splitting HNP, are discussed in the following sections.

#### 4.1.1 Structural Effects

The ability to control the size and morphology of both HNP components along with their material combination and their architectures was addressed in previous sections. This allows a systematic and in-depth investigation of the influence of these structural parameters on the overall photocatalytic efficiency.

##### 4.1.1.1 Semiconductor Component Structural Effects

From the semiconductor component point of view, their size was shown to affect the hydrogen generation activity in different manners. Optical properties along with charge-separation and e–h recombination processes should be considered in this respect. For smaller nanoparticles, a larger driving force for electron transfer is observed due to the quantum confinement-induced increase in the energetic offset increasing between the electronic states of the semiconductor with respect to the metal, as shown for Pt-decorated CdS HNPs in various sizes (Figure 26A) in which 2.8 nm HNPs displayed a H_2_ generation quantum efficiency of 17.3%, much higher than the 11.4% for their 4.6 nm diameter counterpart (Figure 32B).^206^ Additionally, the distance that the charge carriers have to migrate to the active site decreases, and therefore, the probability of loss through the competing electron–hole recombination route decreases accordingly.^236^ An opposite size effect was reported by Amirav et al. showing higher hydrogen production quantum yields for longer rod structures of heterostructured type II HNPs, CdSe/CdS–Pt NRs.^237^ This trend was explained as due to better spatial charge separation along the rod length (where holes are localized to the seed region) and reduced back-recombination from the metal.

Different considerations were demonstrated for CdSe–Pt NRs with single- and double-tipped structures. In this case, an optimal intermediate rod length of 15–20 nm was found to maximize the hydrogen-evolution rate (Figure 32C). The two opposing factors setting this nonmonotonic behavior are the electron-transfer rate, which decreases with increased length (Figure 26B), and the absorption cross section, which increases with the rod length.^207^ A similar nonmonotonic photocatalytic hydrogen generation activity was reported for Au–CdS core/shell HNPs with optimal 8.2 nm shell thickness correlating with its charge-transfer dynamics as was discussed in section 3.3.2.^142^ Additional size effects of the semiconductor component were demonstrated on CdSe/CdS–Pt core/crown NPLs with different core and crown sizes. Kinetic measurements of hydrogen generation showed higher photocatalytic water reduction rates for hybrid NPLs with both a large core and crown followed by a large core with small crown and small core with large crown with calculated apparent quantum efficiencies of 19.3%, 17.8%, and 13.2%, respectively (Figure 32D).^43^

Comparing the influence of morphology on the photocatalytic performance of different semiconductor components indicates that NPLs are favored over NRs and spherical HNPs structures. The vicinity of both hybrid components in 0D structure in comparison to the other 1D and 2D morphologies leads to inferior charge separation and faster charge recombination.^238^ Quantum efficiencies of hybrid NPLs such as CdS–Ni^74^ and CdS–Pt^61^ were reported to exceed complementary NR structure efficiency values^71,197,223,239^ (64% and 42% versus 3–27%). The advantage of the 2D structure over the 1D morphology is attributed to the elongated lifetime of the charge-separated state in the 2D platelet. Applying the hole hopping model to simulate charge recombination indicates that many more random walk steps are required in the NPL case before the hole finds the recombination (Pt) site.^61^

Another aspect of the semiconductor structure that can influence the photocatalytic performance is the electronic profile of the semiconductor. HNPs with a type II or quasi-type II heterostructured semiconductor component, such as CdSe/CdS–Pt^237,240^ and ZnSe/CdS–Pt^201^ NRs, have been reported to exhibit enhanced photocatalytic hydrogen generation in comparison to HNPs with single-phase semiconductor components due to improved charge separation. A different strategy suggested by Li and co-workers includes the alloying of different elements in the semiconductor component, presented by Cu_1.94_S–Zn_*x*_Cd_1–*x*_S–Pt HNPs. In this manner, two light-absorbing regions are synthetically achieved with a tunable energy band gap as a function of Zn mole fraction that can modify the hybrid band alignment to attain a material combination that manifests more efficient charge transfer and photocatalytic activity (Figure 32E).^132^ Similar trends were demonstrated on Zn_1–*x*_Cd_*x*_Se–Pt NRs with various compositions of the semiconductor segment. The optimal composition for efficient hydrogen generation was found to significantly depend on the presence of the Pt domain.^241^ The interplay between the band alignment and the absorption capacity that dominated the pristine semiconductor photocatalytic activity was altered by charge transfer and the electron’s mobility properties that accompanied the deposition of the Pt site.

The electronic profile can also determine the overall energy band alignment and therefore the actual overpotential for proton reduction. This was manifested by core–shell CdSe/CdS nickel-decorated HNPs in which the hydrogen generation rate increased with decreased core size and increased with the increasing shell thickness.^242^ The former trend is attributed to the quantum confinement effect of raising the lowest conduction band level, while the latter originated from a large barrier for electron–hole recombination formed by the thicker shell. As important as the electronic profile is, in a comparative study of two types of HNPs with a type II band structure ZnSe/CdS–Pt and ZnTe/CdS–Pt NRs, the latter showed negligible photocatalytic activity.^46^ The authors assigned this different behavior to a mismatch of the ZnTe conduction band compared with the highest occupied molecular orbital (HOMO) of the surface ligand (MUA), and thus, holes were not scavenged effectively in this case, unlike the behavior for the structure with a ZnSe core.

An additional approach for accelerating hole scavenging was suggested via surface etching of the rod shell in CdSe/CdS–Pt NRs, thereby increasing the hydrogen production rate by 3–4-fold (Figure 32F).^243^ A different approach suggested improved charge transfer to the metal sites by passivating surface traps through postsynthesis addition of a CdS layer over CdSe–Pt nanodumbbells resulting in an increase of 6.5 times in the photocatalytic hydrogen generation rate compared to bare CdSe–Pt HNPs.^244^

Importantly, as part of the quest for an alternative nontoxic and earth-abundant material to replace the heavy-metal semiconductor in general and Cd-based HNPs in particular, HNPs such as ZnSe–Au,^30^ ZnSe–Pt,^76^ and CZTS–Au^25,36,53^ NRs were synthesized and shown to achieve photocatalytic hydrogen evolution activity that exceeded their pristine semiconductor complementary NPs.

##### 4.1.1.2 Metal Component Structural Effects

The metal component, often named the cocatalyst in the context of the photocatalytic activity of HNPs, also plays an important role in the overall photocatalytic water reduction efficiency. As described in section 2.3, synthetic developments allowed the control of the HNPs morphology and architecture. Control over the cocatalyst size and material composition was achieved. Moreover, either selective deposition of the metal domain in the form of a single to several sites on the semiconductor segment or random multiple metal islands across the entire semiconductor surface (assigned to surface defect growth) may be compared. These different decorations along with different metal combinations were shown to have a significant impact on the photocatalytic hydrogen generation performance.

**Figure 33:**
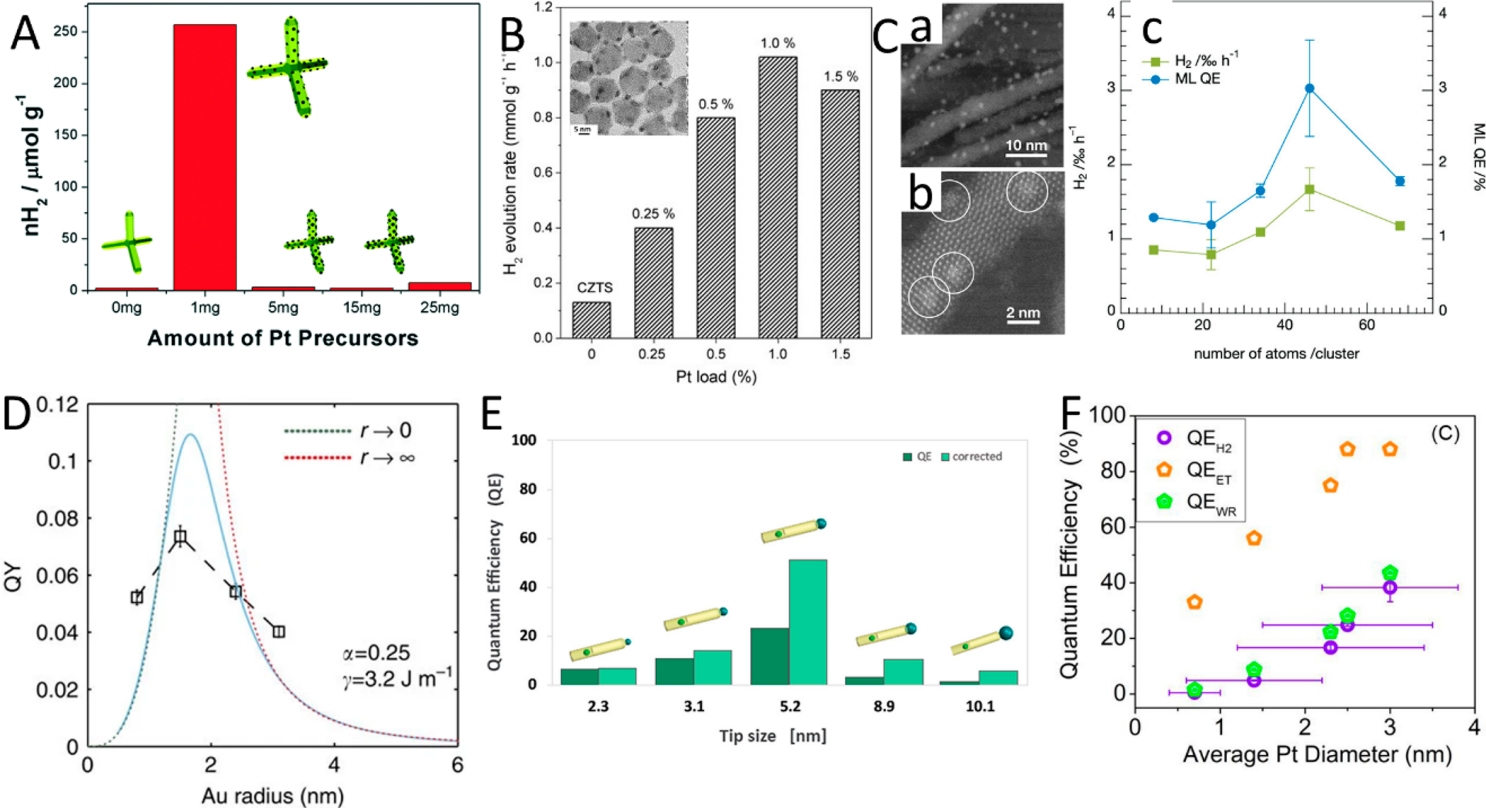
Metal domain size effect on photocatalytic activity of HNPs. (A) Photocatalytic hydrogen generation of the Pt-decorated CdSe tetrapods with different sizes of Pt nanoparticles as a function of the amount of Pt precursors used for the decoration. (B) Dependence of the H_2_ evolution rate on the Pt load on CZTS–Pt heterostructures. (C) (a, b) HAADF-STEM micrographs of Pt_46_ clusters size with monodispersity coverage deposited onto CdS nanorod thin films. Clusters are homogeneously distributed over the CdS nanorods with an average number of clusters per NR, about 23 clusters per rod. (c) Photocatalytic activity of CdS NRs decorated with size-selected Pt clusters as a function of cluster size. Average H_2_ production rate clearly changes with the size of the clusters deposited. (D) Measured photocatalytic H_2_ production quantum yield (black squares connected by dashed line) along with the nonmonotonic kinetic model behavior (blue solid line). Green and red dotted lines represent limiting behaviors of the model for zero and infinite metal domain sizes, respectively. (E) Photocatalytic quantum efficiency for H_2_ evolution obtained with CdSe/CdS–Ni HNPs decorated with different-sized Ni tips. Experimental quantum efficiency in dark green bars, and quantum efficiency corrected for metal absorption in light green bars. (F) Photocatalytic H_2_ production internal quantum efficiency (QE_H2_) of CdS–Pt NRs as a function of the average Pt tip size. Also shown is the electron-transfer quantum efficiency (QE_ET_) and the water reduction quantum efficiency (QE_WR_). (A) Adapted with permission from ref (245). Copyright 2015 Royal Society of Chemistry. (B) Adapted with permission from ref (25). Copyright 2014 American Chemical Society. (C) Adapted with permission from ref (114). Copyright 2013 American Chemical Society. (D) Adapted with permission from ref (28). Copyright 2016 Nature Publishing Group. (E) Adapted with permission from ref (117). Copyright 2018 American Chemical Society. (F) Adapted with permission from ref (116). Copyright 2022 American Chemical Society.

The size is a well-known essential parameter in many different properties of HNPs. The size effect of the metal cocatalyst domain on the hydrogen generation efficiency was addressed in various HNPs systems. HNPs with random metal decoration including CdSe–Pt tetrapods,^245^ CZTS–Pt HNPs,^25^ and CdS NRs decorated with multiple Pt clusters^114^ (8–68 atoms) showed optimal hydrogen evolution at an intermediate metal size or metal loading (Figure 33A–C). However, in this metal decoration form, the ability to isolate the size effect from other contributing effects and to control the actual size of the cocatalysts rather than the weight percentage loading of the metal is quite limited. This effect may be better addressed via single-metal deposition in various metal sizes with narrow size distributions as was demonstrated for CdS–Au NRs^28^ and in following studies on CdSe/CdS–Ni^117^ and CdS–Pt^116^ nanorod structures. Systematic investigation of the cocatalyst effect in the former hybrid nanosystem revealed a nonmonotonic dependence in which an intermediate Au tip size provides the optimal hydrogen evolution rate. This behavior is in contrast to measured charge-transfer rates which increased with increased metal domain size as was described in section 3.3.1. This essential behavior was captured by a minimal kinetic model. This model included all optional decay routes of the excited charge carriers, while charge separation was accounted for by applying a Fermi golden rule formula along with the water reduction reaction at the metal domain surface considered to behave as a Butler–Volmer equation. The kinetic model simulation for hydrogen generation efficiency is presented in Figure 33D and manifests the nonmonotonic dependence of the overall efficiency of photocatalytic hydrogen production on the Au tip size, consistent with the experimental measurements.^28^

A similar nonmonotonic trend was reported in CdSe/CdS–Ni^117^ and CdSe/CdS–Pd^127^ NRs in which an optimal diameter of the Ni site of around 5.2 nm was observed within the range 2.3–10.1 nm (Figure 33E) and Pd diameter site of 2.2 nm within the range of 1.5–4.5 nm. Interestingly, experimentally, in CdS–Pt NRs, increasing the metal site size from 0.7 to 3 nm led to an increase in the quantum efficiency of H_2_ production by nearly 2 orders of magnitude, consistent with the previous studies (Figure 33F).^116^ The authors pointed out that in contrast to the earlier reports, the photocatalytic efficiency is predicted to increase with the increased Pt sizes according their kinetic calculations. Note that the morphology and the evolving facets reactivity changes upon increased metal domain, which were not included in that study, can also influence the photocatalytic performance. Nevertheless, these divergences can originate from the inherent differences of different metal types.

Interestingly, this trend of metal domain size effect was not preserved under nonlinear excitation conditions that promote MX generation. Under high-energy fluences, an advantage of larger tipped HNPs over small-tipped HNPs was revealed for photocatalytic water reduction and hydrogen generation.^218^ This change in the size effect trend is due to the presence of an additional competing relaxation route of Auger recombination at this excitation regime which can be outcompeted by the faster electron charge transfer of large-tipped HNPs, while for a smaller metal domain, the Auger process dominates the relaxation resulting in less free charges at the metal domain available for the water reduction reaction.

**Figure 34:**
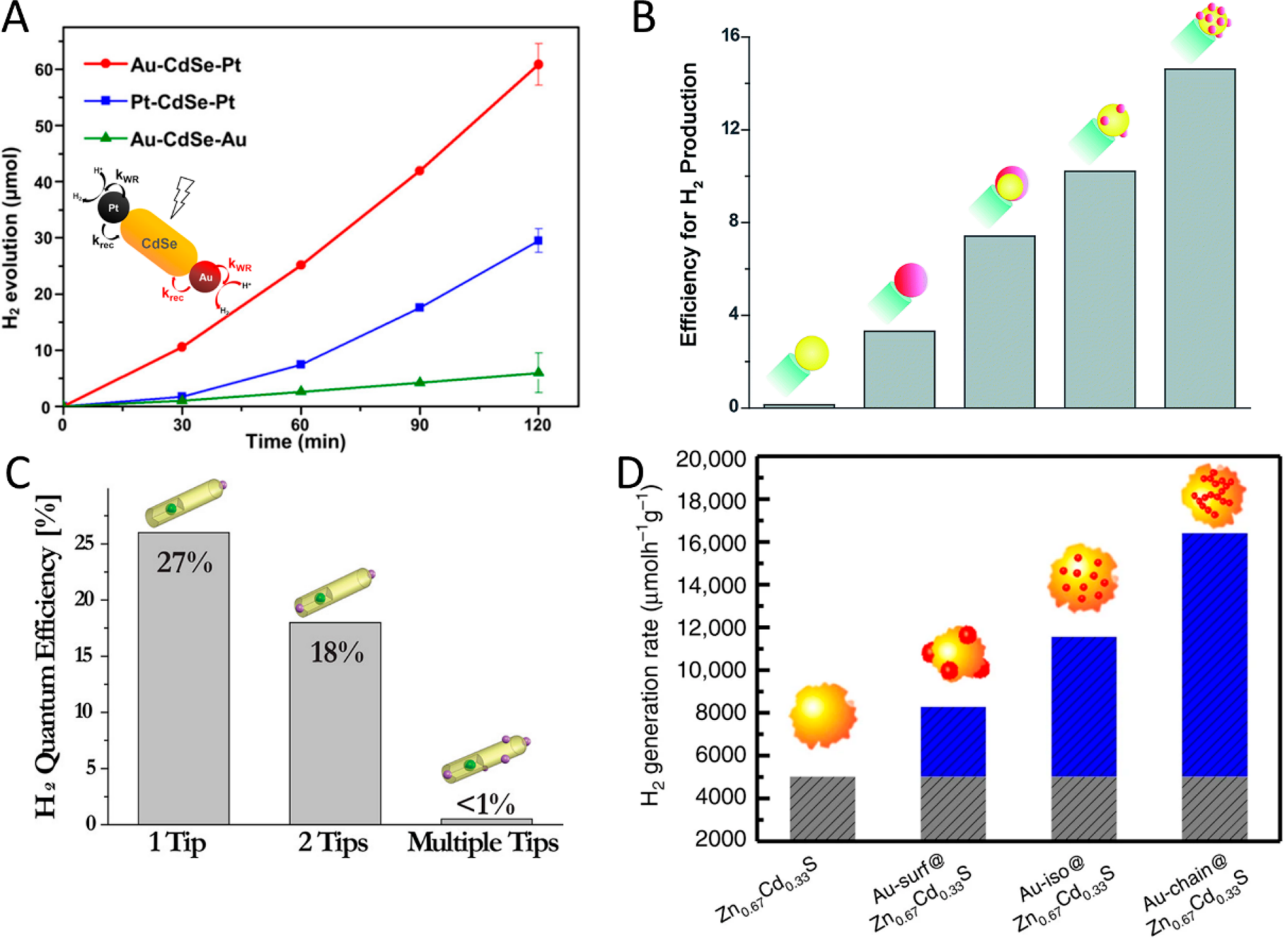
Metal type and decoration architecture effects on photocatalytic activity of HNPs. (A) Photocatalytic H_2_ evolution by Au–CdSe–Au (green), Pt–CdSe–Pt (blue), and Au–CdSe–Pt (red) nanodumbbells in a 0.35 M Na_2_SO_3_/0.25 M Na_2_S aqueous solution. (B) Relative efficiency for the photocatalytic H_2_ production utilizing CdSe/CdS NRs decorated with different metal cocatalysts serving as reduction sites: Au (yellow), Pt (red), Au–Pt core–shell, and Au–Pt. (C) Photocatalytic quantum efficiency for H_2_ reduction obtained with CdSe/CdS NRs decorated with a single, double, or multiple Pt metal domains. (D) Photocatalytic H_2_ evolution activity of pure Zn_0.67_Cd_0.33_S and Zn_0.67_Cd_0.33_S–Au with different spatial arrangements of Au nanoparticles under visible-light irradiation; blue columns represent the enhancement promoted by plasmonic–Au nanostructures. (A) Adapted with permission from ref (134). Copyright 2017 American Chemical Society. (B) Adapted with permission from ref (68). Copyright 2015 Royal Society of Chemistry. (C) Adapted with permission from ref (223). Copyright 2015 American Chemical Society. (D) Adapted with permission from ref (157). Copyright 2019 Nature Publishing Group.

The type of the cocatalyst was shown to have a substantial effect on both charge separation and photocatalytic activity. The photocatalytic efficiencies of HNPs with Au and Pt decorations, which are two of the most common noble metals that serve as cocatalysts in the HNP system, present a characteristic behavior that is derived from their charge-separation and -transfer processes on top of the well-known superior catalytic activity of Pt.^17,25,68,134,146^ Photocatalytic activity measurements comparing CdSe–Pt and CdSe–Au NRs showed the higher photocatalytic efficiency of the Pt-decorated HNPs due to the efficient depletion of excited electrons.^147^ This advantageous performance was assigned to their ohmic behavior, which enables the full extraction of electrons from the lowest excited states in the semiconductor component, while Au-tipped HNP’s photocatalytic capacity may be restricted by Fermi-level equilibration and charge accumulation associated with gold metal NPs.^18^ Note that although Pt is considered more effective toward water reduction, a higher efficiency of aerobic reduction processes such as H_2_O_2_ formation by dissociation of the oxygen molecules was obtained by Au as a cocatalyst site.^246^ An approach that aimed to exploit the two different behaviors of Au and Pt was manifested in the formation of asymmetric Au–CdSe–Pt nanodumbbells which presented higher hydrogen generation rates over Pt–CdSe–Pt and Au–CdSe–Au nanodumbbells. Combination of the slow electron–hole recombination behavior of the Au with the efficient water reduction reaction rate obtained by Pt decoration promoted the more effective nanosystem of the three (Figure 34A).^134^ A similar strategy of asymmetric HNPs was employed in the synthesis of Au–CdSe/CdS–PdS NRs. Again, the energy band alignment of this structure, in which energy band gaps of CdS and PdS are 2.4 and 1.6 eV, respectively, promotes efficient charge separation resulting in a hydrogen generation rate of over 2 orders of magnitude greater than the production achieved by CdSe/CdS and Au–CdSe/CdS.^131^ Larger asymmetry was also reported as a favorable structure comparing the Au–CdS core/shell and heterodimers with different phase separation.^85^ In addition, similar factors of Fermi-level shifting and enhanced electron–hole recombination have been reported to affect the photocatalytic activity of CdS and ZnS–CuInS_2_ NRs with various Pd_4_S and PdO cocatalysts compared to Pt deposition.^50,247^

An understanding of the different photocatalytic characteristics of various cocatalyst metal types led to their combination in order to maximize the hydrogen generation efficiency. Several studies have demonstrated the pivotal effect of the metal site composition. Kalisman et al. showed enhanced photocatalytic H_2_ generation by a cocatalyst consisting of Au tips decorated by Pt islands deposited on the apex of CdSe/CdS NRs compared to Au/Pt core/shell-tipped NRs and single-phase Au- or Pt-based HNPs (Figure 34B).^68^ This trend was attributed to the formed Au/Pt interfaces that promote higher reactivity toward surface bonding interactions. A similar trend was reported for Pt–CdSe–Au and Au–CdSe–Au nanodumbbells where the latter was coated with Pt and a higher photocatalytic activity was measured compared to the original hybrid forms.^134^ A significant improvement in the photocatalytic water reduction rate was also reported for Au/Pd alloy-tipped CdSe/CdS NRs compared to core/shell bimetal sites and single-phase metal deposition, showing an approximately 5-fold increase, which was attributed to the synergistic electronic effects of bimetal catalysts such as new surface rearrangement that decreases the absorbance strength and therefore promotes product dissociation.^67^ Moreover, the photostability of such alloyed sites exceeded that of Pd-tipped NRs due to suppression of the cation exchange of Cd atoms in the rod structure with the presence of Au that serves as a barrier for Pd migration.^248^ The advantage of bimetallic cocatalysts over a homogeneous metal cocatalyst was also reported for Zn_0.5_Cd_0.5_S decorated with Ni/Co alloy NPs,^128^ CdS decorated with several different noble metal–PdS cocatalysts,^249^ and Pt–Pd hybrid NPs deposited on CdS NRs,^129^ all exhibiting higher photocatalytic hydrogen generation rates compared to the single-metal phase-deposited nanosystems due to the lower Fermi energy of the alloyed tipped HNPs or the higher activity of the bimetal interface, respectively. Nevertheless, measurements of the photocatalytic efficiency of Ag/Pd core/shell and alloy cocatalysts deposited on CdSe/CdS NRs showed inferior efficiency compared to the monometallic Pd-tipped NRs.^127^

In addition to the material features of the cocatalyst sites, the decoration architecture also has a critical impact on the photocatalytic hydrogen generation performance. The superior photocatalytic activity of a single cocatalyst domain selectively deposited on the semiconductor absorber was established through several studies in different HNP systems. In the case of CdSe–Pt NRs, a single tip revealed ∼50% higher hydrogen generation rates in comparison to dumbbell CdSe–Pt NRs.^146^ CdS–Pt,^197^ CdSe/CdS–Pt,^223^ and ZnSe–Au^30^ HNPs have shown a similar trend of reduced H_2_ generation quantum efficiency for the case of random multiple cocatalyst sites on the semiconductor surface (Figure 34C). Although excited charge carrier transfer in these HNPs was faster and more efficient, as was described in section 3.3.1, effectively, the vicinity of the trapped hole and the electron on the metal domain results in a faster recombination rate, outweighing the faster electron transfer and therefore dominating the kinetics of the overall photocatalytic process. Alternatively, it was suggested that the probability of harvesting two electrons following two sequential photon absorption incidents, which are required for photoreduction of water to hydrogen, is more likely to occur in the presence of a single cocatalyst site, whereas multiple sites randomly share the excited electrons, hence practically demanding more successive charge-transfer events. Interestingly, a recent report by Liu and co-workers presented the enhancement of photocatalytic hydrogen evolution by multiple plasmonic Au NP chain-like structures embedded in Zn_0.67_Cd_0.33_S NPs in comparison to Au metal domains deposited on the surface of the semiconductor component and even embedded separated Au NPs within the semiconductor (Figure 34D).^157^ This improvement is attributed to two main factors. One is the shorter distance between the semiconductor and the plasmonic Au that can induce more energy transfer. Second, the chain structure holds a short distance between adjacent NPs, which gives rise to highly intense and localized electromagnetic fields. Consequentially, the collective excitation of plasmonic metal facilitates much more plasmonic energy transfer from the metal to the semiconductor that eventually increases exciton formation and the following photocatalytic reaction.

#### 4.1.2 Surface Effect on Photocatalytic Hydrogen Production

In colloidal HNPs, due to their nanometric characteristics such as a large surface to volume ratio, the nature of the organic capping layer has a central influence on the photocatalytic performance via several optional mechanisms. Besides their well-known stabilizing function that allows their dispersion in both aqueous and organic media, the dynamics of the charge-separation process can be altered by different surface coatings via a direct influence on hole removal and charge transfer, as described in section 3.3.1. The specific dependence of the overall photocatalytic activity is derived from the type of organic molecules that are in the stabilizing layer and the nature of the chemical bond between the atoms at the HNP surface and the molecular ligands.

**Figure 35:**
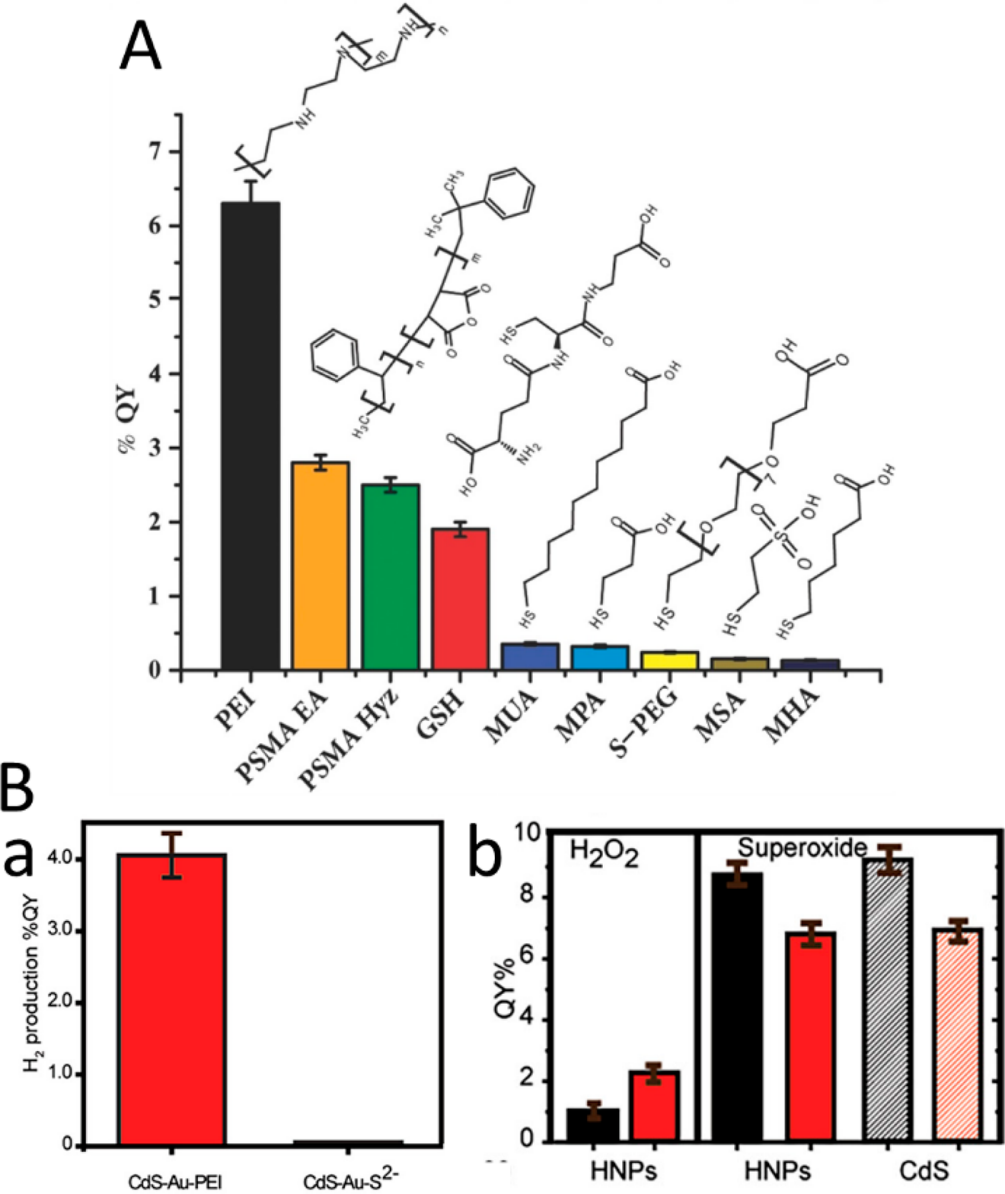
Surface-capping ligands effect on photocatalytic activity of HNPs. (A) Apparent photocatalysis H_2_ evolution quantum yield values for a wide range of surface coatings including thiolated alkyl ligands, glutathione (GSH), and polymer coating. PEI exhibits the highest quantum yield. (B) (a) Percent quantum yield of photocatalytic H_2_ production of PEI and sulfide-coated CdS–Au HNPs. (b) Calculated quantum yield for H_2_O_2_ (left) and superoxide (right) production by HNPs (solid bars) and pristine NRs (checkered bars). (A) Adapted with permission from ref (32). Copyright 2015 Wiley. (B) Adapted with permission from ref (256). Copyright 2021 American Chemical Society.

The effect of the surface capping ligand on the efficiency of hydrogen generation was investigated in various hybrid and semiconductor nanosystems.^250−253^ The implications of this effect on semiconductor–metal HNPs were reported by Banin and co-workers on a single Au-tipped CdS NRs. Investigation of the photocatalytic activity and efficiency of HNPs with different types of surface capping ligands has revealed a significant surface effect.^32^ Comparison of commonly used thiolated alkyl ligands with polymer encapsulation by amphiphilic or branched polymers revealed the superior photocatalytic performance by the polymer coating represented by PEI followed by the amphiphilic polymer coating in the form of poly(styrene-*co*-maleic anhydride) which exhibited an intermediate quantum yield, while the thiolated alkyl ligand showed inferior activity, as presented in Figure 35A. Although several possible mechanisms can be assigned to the surface effect including colloidal stability or photostability that can be hampered in the presence of thiol-based ligands and alternatively the nature of the diffuse electric double layer that may attract or withdraw sacrificial hole scavenger agents, these mechanisms were shown to have minor contributions. Instead, the degree of surface traps passivation was found to dominate this effect. Improved passivation PEI led to reduced hole trapping and thus limited electron transfer to the metal domain due to electron–hole Coulomb interactions. The surface ligand effect was also reported for CdS–Pt HNPs showing a slightly different hydrogen evolution photocatalytic capacity between MUA and cysteine-capped HNPs.^254^ However, for HNP-based photocatalysts where the photocatalytic reactions take place at the semiconductor segment via defect surface states, an inverse trend is expected, where better surface passivation caused inferior photocatalytic activity, as was reported for spherical CdS/CdS core/shell NPs.^255^

It is worth noting that capping surface ligands can also dictate the photocatalytic reaction that can be executed by the HNPs. Demonstration of such orientation was obtained by sulfide treatment of Au-tipped CdS HNPs.^256^ Post-treatment HNPs revealed negligible H_2_ production compared to similar PEI-coated HNPs. Yet, oxidation processes on the semiconductor surface toward radicals (OH^•^ and O_2_^–•^)) were significantly enhanced (Figure 35B).

#### 4.1.3 Chemical Sacrificial Effects

The above sections discussed the photocatalytic activity and efficiency dependence by inherent HNP parameters; nevertheless, the hole removal process and excited charge-transfer routes and therefore the overall photocatalytic performance can be affected by external parameters such as the sacrificial hole scavenger agents or the chemical conditions. Typically, these external factors are aimed to enhance the hole extraction process following light-induced charge separation given the consideration of this pathway as the bottleneck for an efficient photocatalytic hydrogen generation reaction.

**Figure 36:**
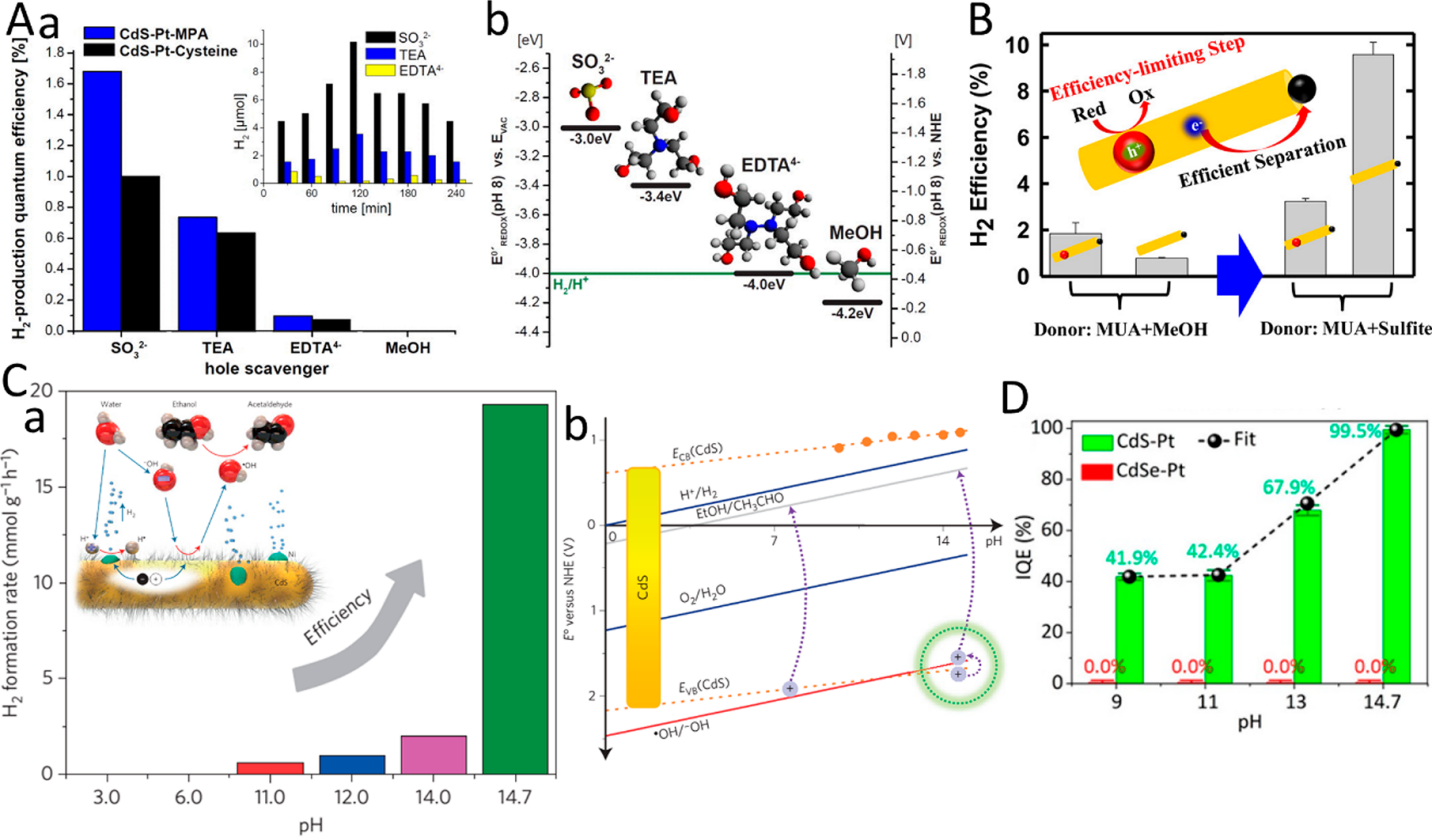
Chemical sacrificial effects on photocatalytic activity of HNPs. (A) (a) Comparison of H_2_ production quantum efficiencies for CdS–Pt HNPs using SO_2_^3–^, TEA, EDTA^4–^, and MeOH as hole scavengers. (Inset) H_2_ evolution as a function of time for MPA-stabilized NRs and SO_2_^3–^, TEA, and EDTA^4–^ as hole scavengers. (b) Schematic plot of the energy levels of the electrons of SO_2_^3–^, TEA, EDTA^4–^, and MeOH that are involved in the reduction of holes vs vacuum and on normal hydrogen electrode (NHE) scale. (B) Steady-state H_2_ photogeneration efficiency using MUA-capped CdSe/CdS–Pt and CdS–Pt nanorods. (C) (a) Comparison of H_2_ formation rates over 6 h showing strong enhancement at high pH. (Inset) Scheme of the photocatalytic water reduction by CdS–Ni NRs (cysteine stabilized) at alkaline conditions. Photoexcited holes oxidize hydroxyl anions that, as a radical, carry away the positive charges and in turn oxidize ethanol to acetaldehyde. Blue arrows denote movement of the species; red arrows denote a redox reaction. (b) Electrochemical potentials of water reduction and oxidation, hydroxyl anion, and ethanol oxidation follow Nernstian behavior (slope −59 mV/pH), but dependence of CdS conduction and valence bands on pH is only −33 mV/pH. Orange dots correspond to measured conduction band minima. Green circle marks the region where the energy of the valence band (CdS) is more positive than *E*°(^•^OH/^–^OH); violet arrows denote the possibility of a two-step oxidation pathway at high pH versus a single-step process at lower pH. (D) pH-dependent H_2_ generation quantum efficiency of CdS–Pt (green column) and CdSe–Pt (red column) hybrid NPLs. (A) Adapted with permission from ref (254). Copyright 2012 AIP Publishing. (B) Adapted with permission from ref (240). Copyright 2014 American Chemical Society. (C) Adapted with permission from ref (71). Copyright 2014 Nature Publishing Group. (D) Adapted with permission from ref (61). Copyright 2018 American Chemical Society.

The effect of the type of the hole scavenger on photoinduced H_2_ evolution was investigated by Berr et al. on Pt–CdS NRs showing improved photocatalytic efficiency as a function of the negativity of the hole scavenger redox potential. As presented in Figure 36A, a comparison of different electron-donating molecules including methanol, EDTA^4–^, triethanolamine (TEA), and SO_3_^2–^ showed increased hydrogen generation quantum efficiency in the presence of a hole scavenger within this order.^254^ A more negative oxidation potential leads to a larger driving force for hole removal by sacrificial electron–excited hole recombination. Moreover, a low hole reduction rate was correlated with a loss of stability, attributed to photooxidation of the semiconductor rod itself that also hinders the photocatalytic performance. However, these thermodynamic considerations also should include the nature of the HNPs, structure, and material composition as was demonstrated by testing the photocatalytic water reduction activity of CdS–Pt and CdSe/CdS–Pt NRs with two different hole scavengers, methanol and sulfite. As shown in the former described report, the use of sulfite indeed allowed relatively higher efficiencies for both HNP systems in comparison to methanol. Still, comparing these different HNPs in the presence of each hole scavenger reveals an inverse photocatalytic behavior where when using methanol a higher quantum efficiency was observed for CdSe/CdS–Pt, while a higher quantum efficiency with sulfite was achieved in CdS–Pt (Figure 36B).^240^ This trend was attributed to the interplay between the trapped holes at the HNP surface and the reductive nature of the electron-donating agents. For the strong reducing sulfite case, holes in CdSe/CdS-based HNPs are confined to the core and therefore are harder to access in comparison to surface traps that accumulate holes in the CdS structure. Using weak electron-donating agents such as methanol may not be energetically sufficient (small driving force) for reducing strongly bounded holes at the CdS surface compared to holes at the CdSe–CdS core–shell interface.

A different strategy for improving hole removal is by manipulating the reaction environmental conditions. Several reports demonstrated the influence of conducting the photocatalytic water reduction under extreme alkaline conditions (pH > 13). Simon et al. reported a significant 6-fold increase in H_2_ generation rates by Ni-decorated CdS NRs at pH 13–14 in comparison with pH 11–12. At a pH of 14, a steep increase in the reaction rate appeared and an improvement in the photocatalytic function reached a 53% external and 71% internal quantum yield (Figure 36C-a).^71^ The underlying mechanism of this strategy is the formation and utilization of hydroxyl ions (OH^–^) as an efficient redox shuttle with higher mobility and accessibility to the HNPs surface compared to other common hole scavenger agents, allowing faster hole reduction and formation of hydroxyl radical coupled with a high-rate catalytic oxidation reaction by the formed radicals of different electron-donating molecules in the solution such as methanol or TEA. Yet, as illustrated in Figure 36C-b, at pH < 14, reduction of holes is taking place mainly by relatively slow-rate direct oxidation of methanol to acetaldehydes. However, as illustrated in Figure 36C-b, above pH 14, the redox potential of hydroxyl (OH^•^/OH^–^), which follows Nernstian dependence (59 mV per pH unit), crosses and exceeds the energy level of the semiconductor VB, which has a weaker pH dependence (33 mV per pH unit), therefore promoting the reduction of holes via fast and efficient hydroxyl shuttle agents which now dominate the photocatalytic process. Such pH dependence was observed for CdS–Pt NRs as well, showing a steady increase of H_2_ evolution rates upon increasing the pH values from pH 12 (3.6 mmol h^–1^ g^–1^) to pH 13 (21.8 mmol h^–1^ g^–1^).^257^ Similarly, CdS–Pt NPLs^61^ and CdSe/CdS–Pt^239^ NRs were also used to demonstrate the advantage of high alkaline conditions (Figure 36D). Increasing the pH conditions even higher, up to pH 14.7 or 16 (for the former and latter cases, respectively), resulted in unity conversion of photons absorbed to hydrogen.

#### 4.1.4 Toward Full Water Splitting

The promising utilization of HNPs as photocatalysts for water reduction and H_2_ production is clearly manifested throughout the advanced synthesis control and in-depth investigation of their photocatalytic properties that derived from their material combination and structure as was discussed in the former sections. Yet, to complete a full water splitting catalytic cycle that includes both H_2_ and O_2_ formation by water reduction and oxidation, respectively, a hybrid system that can exploit in a single excitonic cycle both charge carriers, electrons and holes, to promote these two catalytic pathways is desired. Generally, semiconductor–metal HNPs until now did not demonstrate efficient activity toward the second half-cell reaction of water oxidation. This is due to the inherent complexity of this multiple-charge carrier reaction that requires four hole charges to generate molecular oxygen from water along with its slow kinetics and high redox potential. As was addressed above, a long-lived charge-separated state ought to be to obtained to enable the four-hole oxidation process required to form the O–O bond of molecular oxygen and to effectively compete with intraparticle fast relaxation routes that lead to electron–hole recombination or surface oxidation.

**Figure 37:**
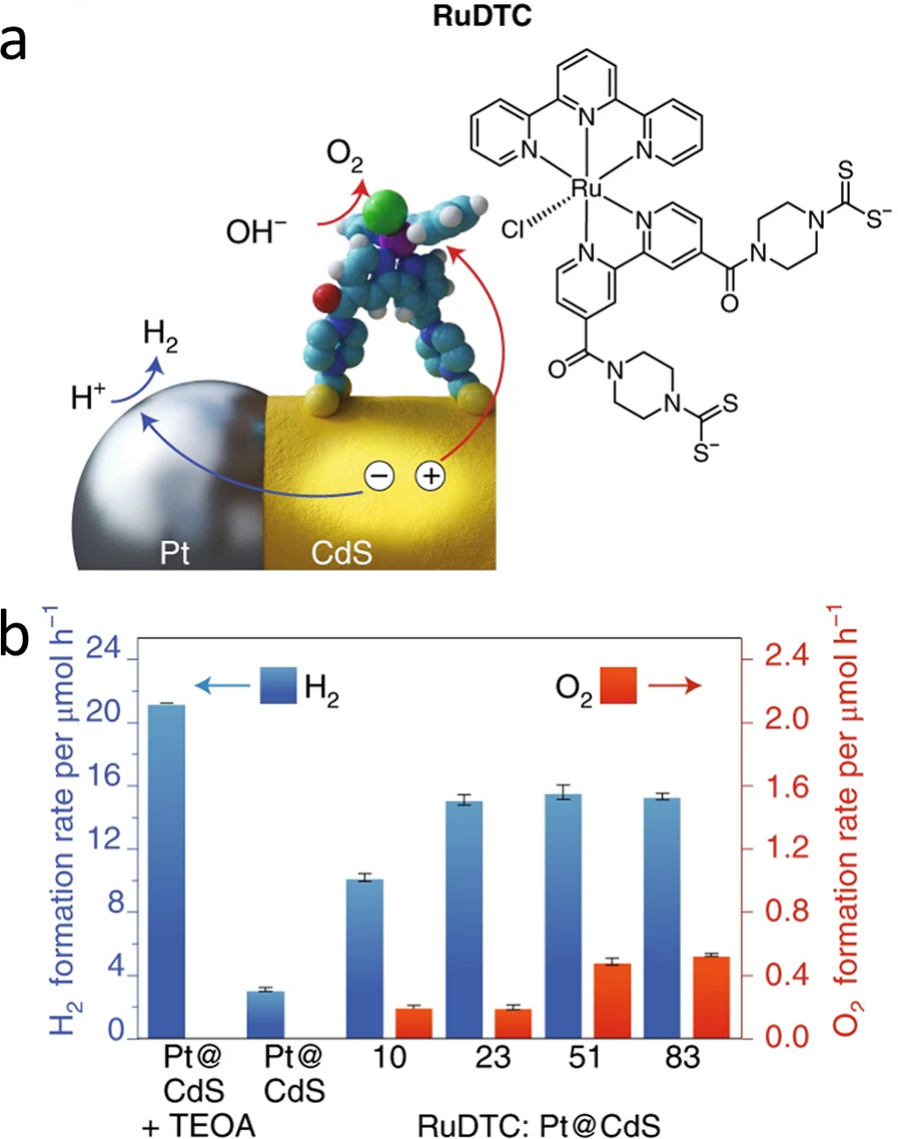
Toward full water splitting by HNP photocatalysts. (a) Schematic of the photocatalytic water-splitting reaction cycle by the modified CdS–Pt photocatalyst. Structure of the Ru-based oxidation cocatalyst, RuDTC, is also shown. (b) Comparison of average H_2_ and O_2_ generation rates over the first hour of illumination by CdS–Pt without RuDTC (with and without the hole scavenger triethanolamine, labeled as TEOA) and CdS–Pt with the additional Ru-based complex as an oxidation cocatalyst in different concentrations. Averages were calculated from at least two measurements for each system. Error bars represent the standard deviation of the measured rates. Adapted with permission from ref (258). Copyright 2018 Nature Publishing Group.

A synthetic effort was made by Alivisatos and co-workers presenting of Ru–CdSe/CdS–Pt HNPs in which the Ru and Pt metal sites served as cocatalysts for the water oxidation and reduction reactions, respectively.^133^ However, no actual photocatalytic measurements of water splitting were exemplified. Recently, Stolarczyk and colleagues demonstrated a full water splitting photocatalytic cycle. The use of well-known CdS–Pt NRs that promote efficient charge separation was proven to effectively reduce water to generate hydrogen along with surface modification by a ruthenium-based complex that was shown to harvest excited holes to catalyze oxygen formation; both water reduction and oxidation were achieved and measured simultaneously (15.1 and 0.52 μmol h^–1^, respectively) (Figure 37).^258^ The Ru complex that consisted of a derivative of the molecular oxidation catalyst Ru(tpy)(bpy)Cl_2_ with dithiocarbamates anchors that attach to the HNP surface allowed a fast hole transfer rate within ∼300 fs. This time scale can successfully compete with the above-mentioned relaxation routes that typically hold a lifetime ranging from 0.7 to 1.0 ps.^259^ Yet, the efficiency of the oxidation reaction achieved by this hybrid system was up to 0.27% and a molar ratio of H_2_ to O_2_ (∼20:1) that was much lower than the stoichiometric value of 2 leave plenty of room for further synthetic developments and fundamental investigations of these HNP-based photocatalysts for addressing the highly challenging full water splitting reaction.

#### 4.1.5 HNP-Based Photoelectrochemical Cells

As discussed in the last section, full water splitting is rare in colloidal HNPs and typically requires a wide band gap due to the high over potential needed to drive the catalytic reactions. On the other hand, to maximize the absorption of the solar spectrum, small band-gap materials are favored. In addition, full water splitting in a colloidal solution on a single nanostructure leads to the formation of O_2_ and H_2_ in the same place, raising concerns about the possibility for their reaction and requiring separation processes, altogether challenging the applicability of this approach. Photoelectrochemical (PEC) water splitting has emerged as a path to address these problems. Here, H_2_ and O_2_ are formed on a separated photocathode and photoanode, respectively. As two different semiconductors may be used for water oxidation and reduction, the band gap of each can be smaller to maximize light absorption. The required overpotential for the chemical reaction is supplied by the electrical properties of the semiconductors such as the majority carriers; p-type semiconductors with high overpotential for water reduction are investigated as photocathode materials, while n-type semiconductors are used as photoanode materials due to the high overpotential for water oxidation. The overpotential to drive the catalytic reaction is further increased by applying external electrical bias, which is yet an additional attribute of the PEC approach. Thus far, HNP PEC cells have been demonstrated as photocathodes photoanodes and tandem cells, but the work in this area utilizing the precontrolled colloidal HNPs is still quite limited compared to the studies of their water splitting performance in suspensions.

Photocathodes made from gold-tipped HNPs have been reported to show superior photoelectrocatalytic performance for the water reduction reaction; for example, Au–Cu_2–*x*_Te in comparison to Cu_2–*x*_Te with photocurrent densities being ∼10 times higher.^260^ Au–CuInS showed enhancement of water splitting of four times in comparison to only CuInS nanostructures.^261^ Photocurrent response in Au–CuGaS_2_ over the pure material was also reported to be enhanced.^262^ This can be attributed to a combination of the contributions of the gold catalytic ability and possibly to the plasmonic enhancement of light absorption.

**Figure 38:**
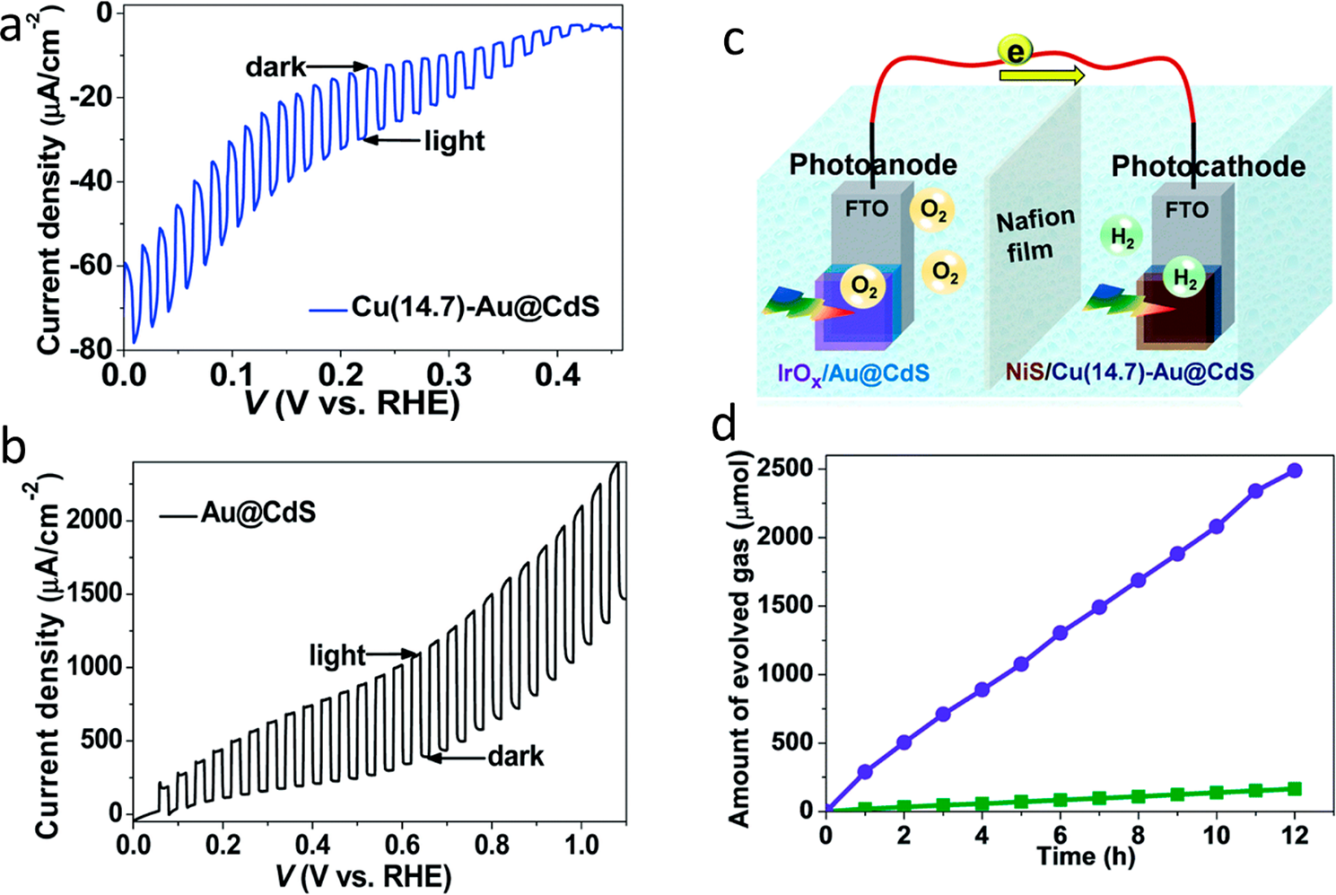
Photoelectrochemical full water splitting from HNPs. Photocurrent density–potential curves for (a) undoped Au–CdS HNPs and (b) Cu–Au–CdS HNPs under simulated sunlight illumination (AM 1.5G, 100 mW cm^–2^) using a three-electrode configuration. (c) Schematic illustration of the configuration of the tandem PEC device. Photoanode made of undoped Au–CdS HNPs on FTO modified with IrOx as the O_2_ evolution cocatalyst, and photocathode was formed by dip coating the Cu(14.7)–Au–CdS HNPs onto FTO with NiS as the H_2_ evolution cocatalyst. (d) Amount of H_2_ (square) and O_2_ (circle) gases evolved from the constructed tandem PEC cell upon 1 Sun light illumination in parallel illumination mode. Adapted with permission from ref (263). Copyright 2019 Royal Society of Chemistry.

Photoanodes with HNPs were also demonstrated. Ag–CdS NW showed a photocurrent density increase by about 4.7 times compared to that of the pure CdS NW photoanode, whereas the H_2_ obtained for Ag–CdS NWs is 1.8 times higher than that for the CdS NW photoanode. As an example of overall water splitting, a tandem cell consisting of an undoped Au–CdS photoanode and a Cu-doped Au–CdS photocathode, respectively, modified with cocatalysts was fabricated and displayed stable H_2_ and O_2_ evolution as demonstrated in Figure 38.^263^

### 4.2 Clean Energy—CO_2_ Reduction

Water splitting is one way to form carbon-free fuel in the form of H_2_, while another clean energy source is the reduction of CO_2_, such that this greenhouse gas is sequestered while no new carbon is emitted to the atmosphere. The energy barrier of the first step in the reduction of CO_2_ is very high, and therefore, even two-electron reduction to CO is challenging. If CH_4_ and CH_3_OH are the main target products, the generation of CH_4_ and CH_3_OH requires the transfer of eight and six electrons, respectively. Due to the high energy barrier and the multielectron reduction process, this reaction requires tight control on the chemistry of the catalytic metal to favor this reaction over other competitive reactions such as water reduction. A common strategy to address this requirement is by coupling the semiconductor to a metal complex to enhance reaction selectivity toward CO_2_. For example, CdS QDs with Ni(cyclam),^264^ Ni doping,^265^ and Co_2_L complex^266^ demonstrated selective photocatalytic CO_2_ to CO conversion. Fe and Co complexes with CuInS_2_ were also used to selectively reduce CO_2_ to CO in water.^267−269^ The metal atoms can also change the selectivity of the CO_2_ reduction products. Under full solar spectrum irradiation, CO as the main product with 97.2% selectivity was observed on bare CdS, whereas the intentional introduction of an optimized amount of Ru metal enables CO_2_ reduction to CH_4_ with 97.6% selectivity.^270^

**Figure 39:**
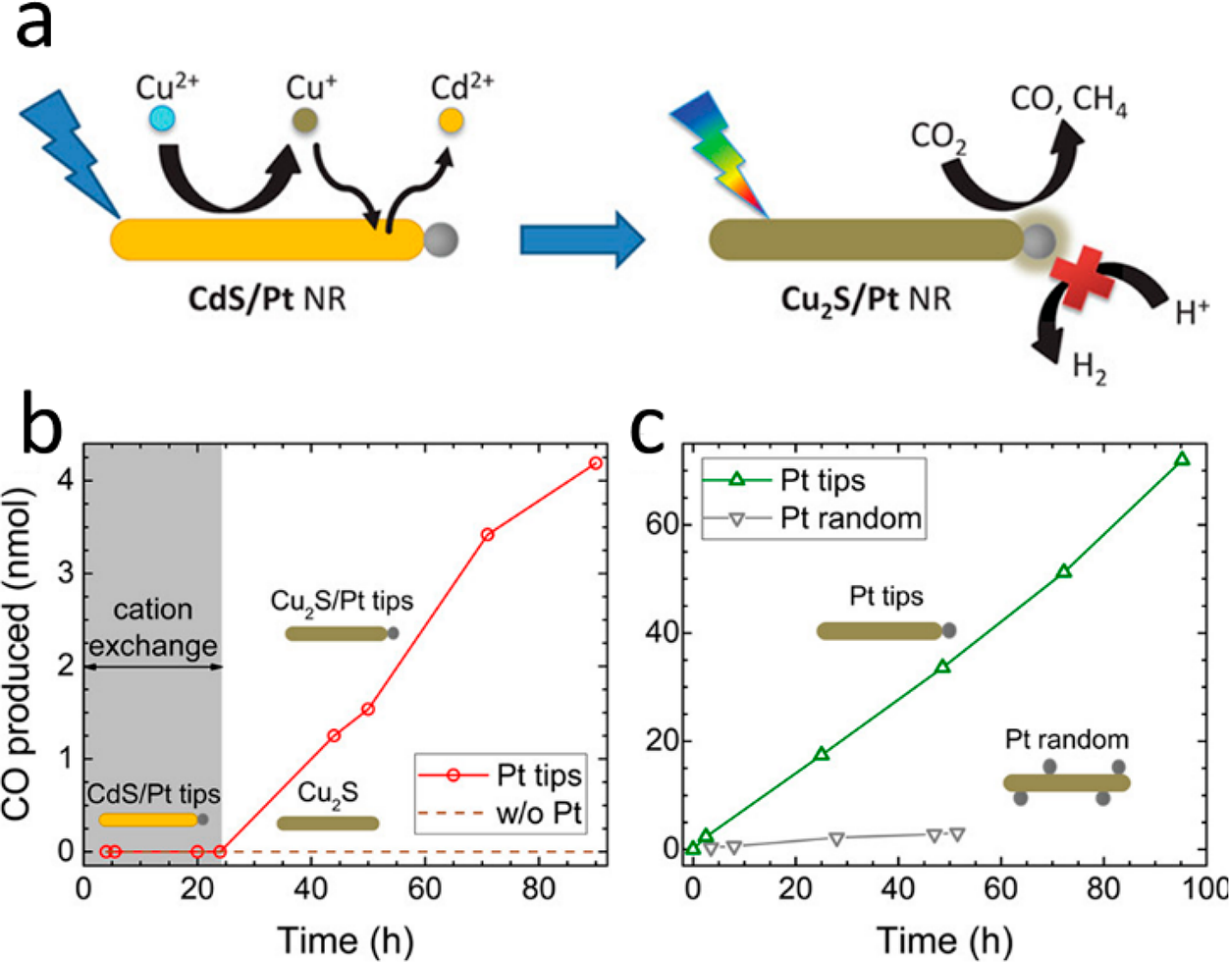
Photocatalytic CO_2_ reduction. (a) Scheme of light-induced cation exchange leading to photocatalytic CO_2_ reduction into CO and CH_4_. CO evolution from CdS–Cu_2_S NRs after illumination (b) with a 447 nm laser or (c) with a broad spectrum Xe lamp. Adapted with permission from ref (271). Copyright 2019 American Chemical Society.

Compared to water splitting, CO_2_ reduction by metal tip deposition is so far less common for chalcogenide-based HNPs. For tip-decorated Cu_2_S–Pt, the CO formation rate was 3.02 μmol h^–1^ g^–1^, 2 orders of magnitude higher than that for random decoration of Pt. Methane evolution was also observed for the tip-decorated sample, albeit with a much smaller formation rate, 0.13 μmol h^–1^ g^–1^ (Figure 39). In this system, both the bare CdS NRs and the NRs with randomly photodeposited Pt NPs did not lead to any gas evolution.^271^ In another report, CdS–Pt was also proven useful for photocatalysis depending on the details of the growth method. In this work, when Pt nanoparticles were reduced onto CdS by ethylene glycol, the CO production rate was 2.99 μmol g^–1^ h^–1^, compared to 0.12 μmol g^–1^ h^–1^ for CdS only and 0.18 μmol g^–1^ h^–1^ for 1 wt % Pt by photoreduction.^272^

Visible-light photocatalysis using Cd–chalcogenide was also demonstrated by combining CdSe with Pt/TiO_2_. In this case, the photocatalytic conversion of CO_2_ to methane and methanol was demonstrated in the presence of water.^273^ This strategy was also reported for a PbS QD used to sensitize Cu/TiO_2_, where the highest total CO_2_ conversion yield was 1.71 μmol g^–1^ h^–1^ (0.82 μmol g^–1^ h^–1^ CO, 0.58 μmol g^–1^ h^–1^ CH_4_, and 0.31 μmol g^–1^ h^–1^ C_2_H_6_), which is >5 times the activity of only Cu/TiO_2_.^274^ Another example for tunable photocatalytic CO_2_ reduction activity by CdS was shown for Au/MoS_2_-tipped CdS NWs. This study reported syngas generation with a H_2_/CO ratio ranging from 0.35 to 3.6 under visible-light irradiation by simply altering the Au particle size.^275^ In this work, CO_2_ reduction to methanol over noble metal-free hybrid semiconductor photocatalyst (Ni_2_P/CdS) under visible light was also reported.^276^

### 4.3 Industrial Applications

The ability to utilize the exciton in HNP for photocatalytic reactions can be further extended to various additional applications. In this section, we show several examples of how these excited charge carriers can be exploited for industrially relevant processes. This includes the use of HNPs for photoinduced catalysis of selective organic reactions, and we also discuss their use as photoinitiators for polymerization. Last, we describe some examples of sensors, including chemical detectors and photodetectors made by HNPs.

#### 4.3.1 Organic Transformations

As shown in the above sections for the reduction of H_2_O, CO_2_, and O_2_, the metal is crucial for the selectivity toward specific reactions. The use of HNPs for selective photocatalysis is also exemplified by using different metal tips to direct the conversion of molecules toward the desired chemical product utilizing a photocatalytic reaction route. Photochemical dehydrogenation and hydrogenolysis of benzyl alcohol was studied on CdS–Pt and CdS–Pd nanorods. CdS–Pt favors dehydrogenation (H_2_) over hydrogenolysis (toluene) with a ratio of 8:1, whereas CdS_0.4_Se_0.6_–Pd favors hydrogenolysis over dehydrogenation by 3:1.^277^

The use of HNPs to catalyze organic chemical reactions was extended also to the near-infrared region, where the Cu_7_S_4_–Pd nanostructure demonstrated photocatalytic activity for Suzuki coupling reactions of iodobenzene with different reagents, selective oxidation of benzyl alcohol, and hydrogenation of nitrobenzene under 808, 980, and 1500 nm irradiation.^278^ Selective reduction of nitroaromatic compounds was also enhanced by Au on CdS NWs.^279^ The importance of the HNPs for selective hydrogenation reactions was also demonstrated in the selectivity of catalytic reaction products. Catalysis of the hydrogenation of cinnamaldehyde shows that while 3-phenyl-1-propanol was the only product over Pt–Cu_2_S HNPs, CuPt–Cu_2_S HNPs exhibited a higher conversion rate and selectivity toward hydrocinnamaldehyde which was also enhanced compared to CuPt only.^280^

#### 4.3.2 Polymerization

**Figure 40:**
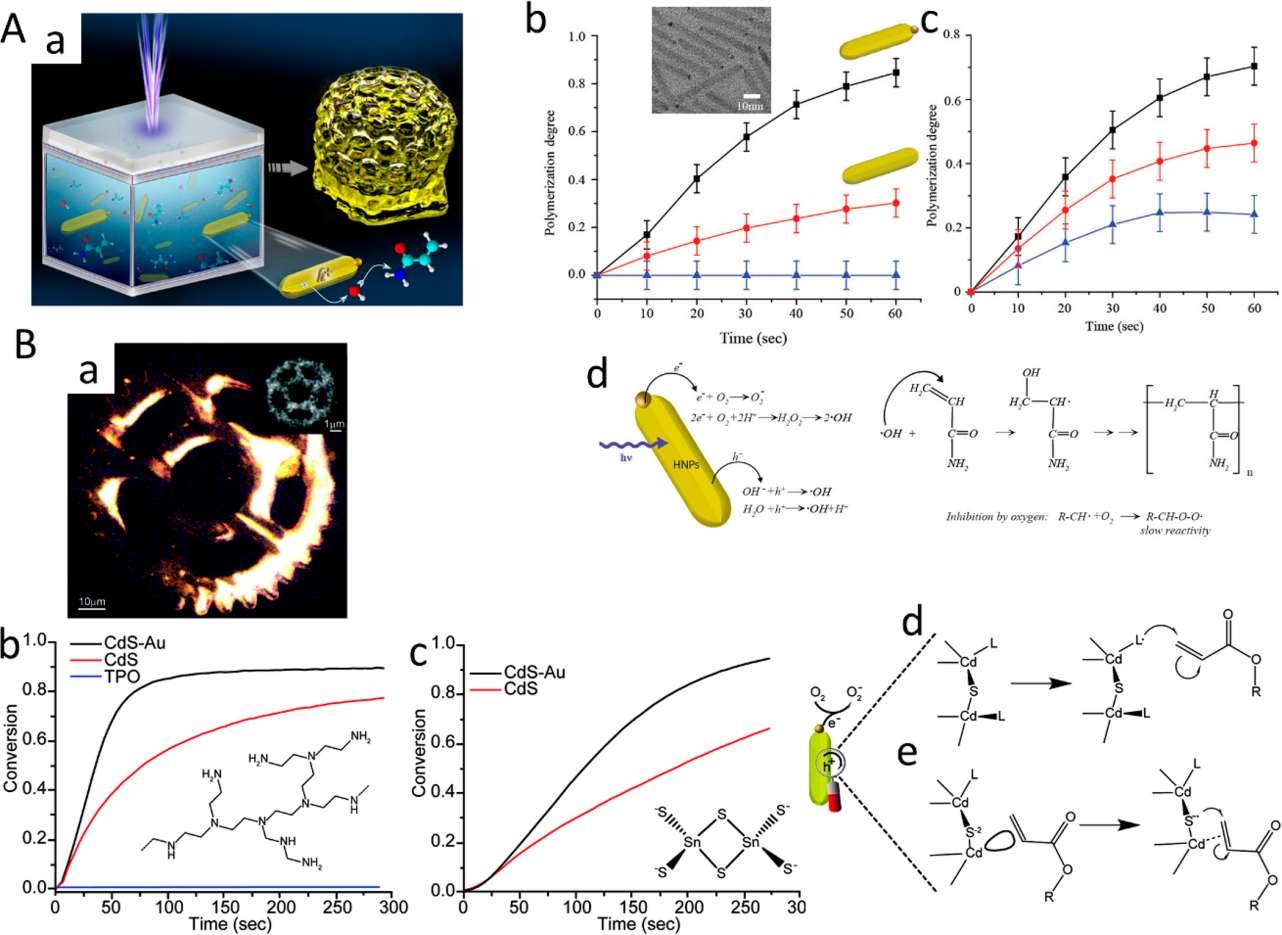
(A) Photopolymerization by HNPs in aqueous solution. (a) Buckyball structure printed in a commercial DLP printer using a water-based formulation with HNPs as photoinitiators. (b) Polymerization degree under UV light at 385 nm using CdS–Au (black), CdS (red), and I2959 (blue) as photoinitiators. (Inset) TEM image of CdS–Au NRs with a 1.5 nm diameter gold tip. (c) Polymerization degree of acrylamide versus time using HNPs as photoinitiators in water (black), in the presence of sulfide/sulfite (red), or in ethanol (blue) as hole acceptors. Addition of both leads to a decay in the polymerization rate of the monomers, indicating that the holes and hydroxyl radicals are essential to the polymerization. (d) Mechanism of photopolymerization by HNPs in water-based formulations. Excited electrons in HNPs reduce O_2_, and holes oxidize H_2_O, producing hydroxyl radicals which initiate a polymerization reaction by attacking the acrylic monomers. (B) Solvent-free polymerization with HNPs. (a) Fluorescence microscopy image of a two-photon polymerized gear printed using the quantum PIs. (Inset) Fluorescence image of a higher resolution structure. (b) Conversion of hydroxyethyl acrylate monomers initiated by polyethylenimine (PEI)-coated NRs (red) and HNPs (black). Diphenyl(2,4,6-trimethylbenzoyl)phosphine oxide (blue) showed negligible conversion. (Inset) Structure of branched PEI. (c) Conversion by Sn_2_S_6_^4–^-coated NRs (red) and HNPs (black). (Inset) Structure of Sn_2_S_6_^4–^. Proposed mechanisms: (d) initiation is carried out by hole transfer from the semiconductor to the monomers via surface coating mediation, (e) double bond is coordinated by the cation followed by a hole transfer from the anion-localized state. (A) Adapted with permission from ref (284). Copyright 2017 American Chemical Society. (B) Adapted with permission from ref (285). Copyright 2019 American Chemical Society.

The excitons in semiconductor NPs emerge as an alternative for organic photoinitiators for polymerization initiation, where the presence of holes can form radicals to initiate a polymerization chain reaction.^5^ The mechanism is either by direct reduction/oxidation of the monomer itself or by some mediator such as amine or hydroxyl radicals.^281−283^ The enhanced charge separation in HNPs and the formation of ROS can also be harnessed for this application. The effect of metal tip in the photopolymerization of polyacrylamide was studied in CdS–Au HNPs. The kinetics of the polymerization degree was followed by the FTIR signature of the acrylamide monomer double bond. CdS–Au demonstrated three times faster polymerization compared to CdS NRs without a metal tip (Figure 40A).^284^

The superior performance of CdS–Au is attributed to the electron–hole charge separation induced by the metal tip accompanied by its favored catalytic functionality toward reduction of O_2_, enhanced rate of electron removal, and enhanced hole removal to the monomer by hydroxyl radicals (see scheme in Figure 40A). This was supported by comparing the polymerization with the presence of the hole scavenger ethanol and sulfide, which led to a significant decrease in the polymerization rate. This observation is explained as the presence of these hole scavengers competes with the monomers on the radicals formed.^284^ In a later work, hydroxyethyl acrylate was used as a monomer in performing the photopolymerization without solvent (solvent free) and in the absence of water. The suggested initiation mechanism in this case was by surface-mediated hole transfer from the semiconductor to the monomers, either directly to the double bond of the monomers or via the surface ligands, polyethylenimine (Figure 40B).^285^

The facile radical generation leading to photopolymerization, accompanied by the ability to tune the semiconductor absorption to the common blue wavelengths used in 3D printers, allowed the generation of a 3D-printed hydrogel structure utilizing such “quantum photoinitiators”. Moreover, the enhanced two-photon absorption cross section akin to semiconductor nanorods allowed the HNPs to be used as photoinitiators for high-resolution 3D printing utilizing a two-photon absorption process. This was demonstrated both for water-based printing and for solvent-free formulation.

#### 4.3.3 Sensing

**Figure 41:**
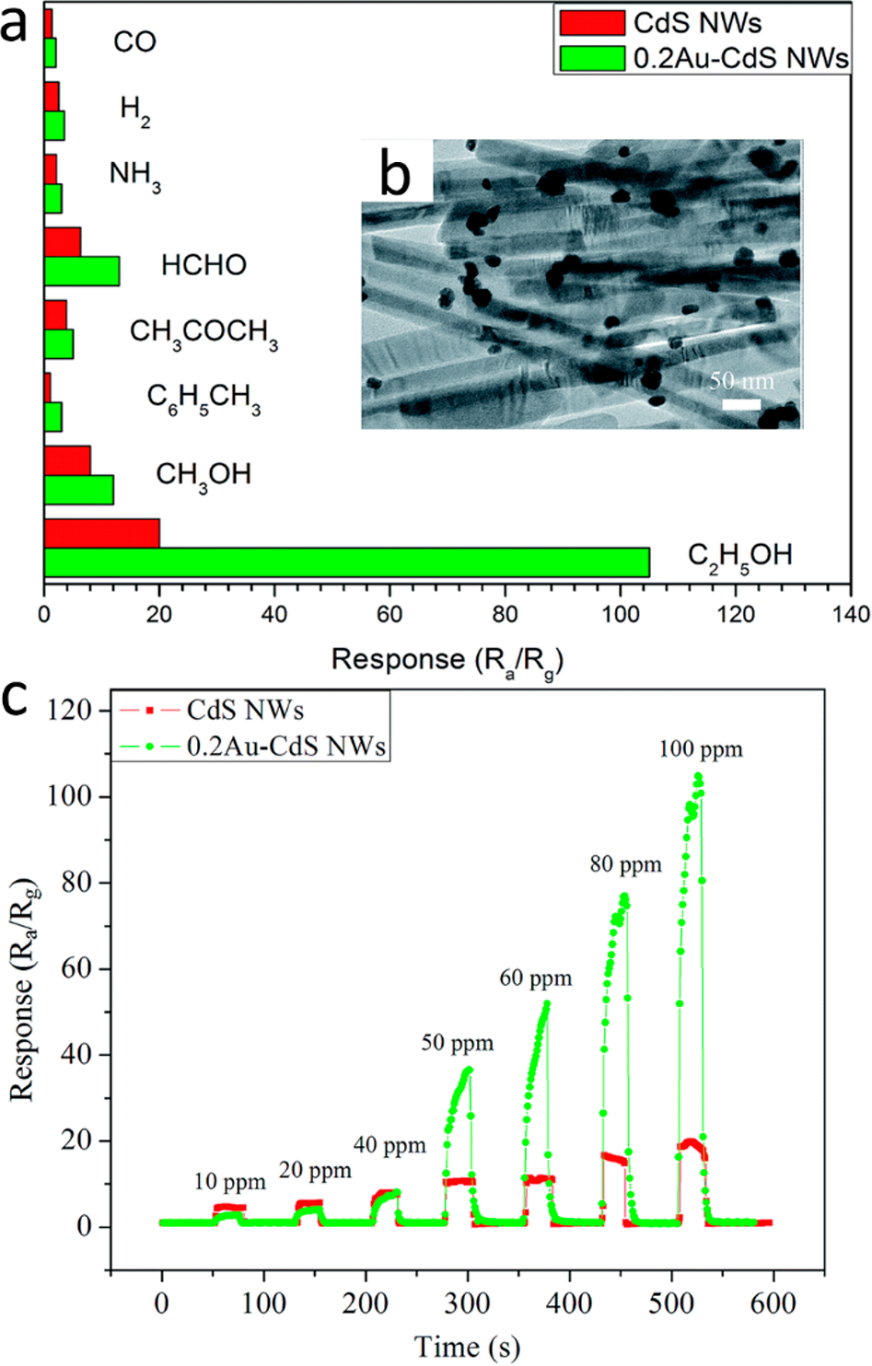
Chemical gas sensing. (a) Selectivity of the sensors to 100 ppm hydrogen (H_2_), carbon monoxide (CO), acetone (CH_3_COCH_3_), ammonia (NH_3_), formaldehyde (HCHO), toluene (C_6_H_5_CH_3_), methanol (CH_3_OH), and ethanol (C_2_H_5_OH). (b) TEM images of 0.2Au–CdS NWs. (c) Dynamic response curves of the sensors versus different concentrations of ethanol. Adapted with permission from ref (286). Copyright 2019 Royal Society of Chemistry.

Combination of the optical and catalytic properties of the semiconductor and metal components was also suggested to be utilized for sensing applications. Photoelectrochemical detectors for Cu^2+^ ions were studied by the deposition of Au tips on CdS NPs. The signal of CdS–Au was enhanced three times upon Au deposition. The suggested mechanism of enhancement was explained by hot-electron transfer from gold to CdS which hinders the recombination of electron–hole pairs in the CdS NPs. Gas sensing was demonstrated on Au-decorated CdS NWs, where the response to ethanol was enhanced 5 times compared to that in nondecorated CdS NWs (Figure 41).^286^ This enhancement was attributed to the better catalytic oxidation of organic molecules in the presence of gold, consistent with the results of enhanced ROS formation by CdS–Au in solution.^246,287^

In the area of light sensors, infrared photodetectors were prepared by the addition of Ag NPs to a film of PbS quantum dots, and the responsivity in the devices increased from 1.5 to 3.8 mA/W for 1% of Ag NPs.^288^ Similar results were reported by the addition of Au NPs to PbS film.^289^ An ultraviolet photodetector was prepared by layers of CdS and Au NPs.^290^ The enhancements of the photodetection were attributed to the suppression of charge carrier recombination by the presence of Au NPs.

### 4.4 Biomedical Applications

#### 4.4.1 Photodynamic and Photothermal Therapies

**Figure 42:**
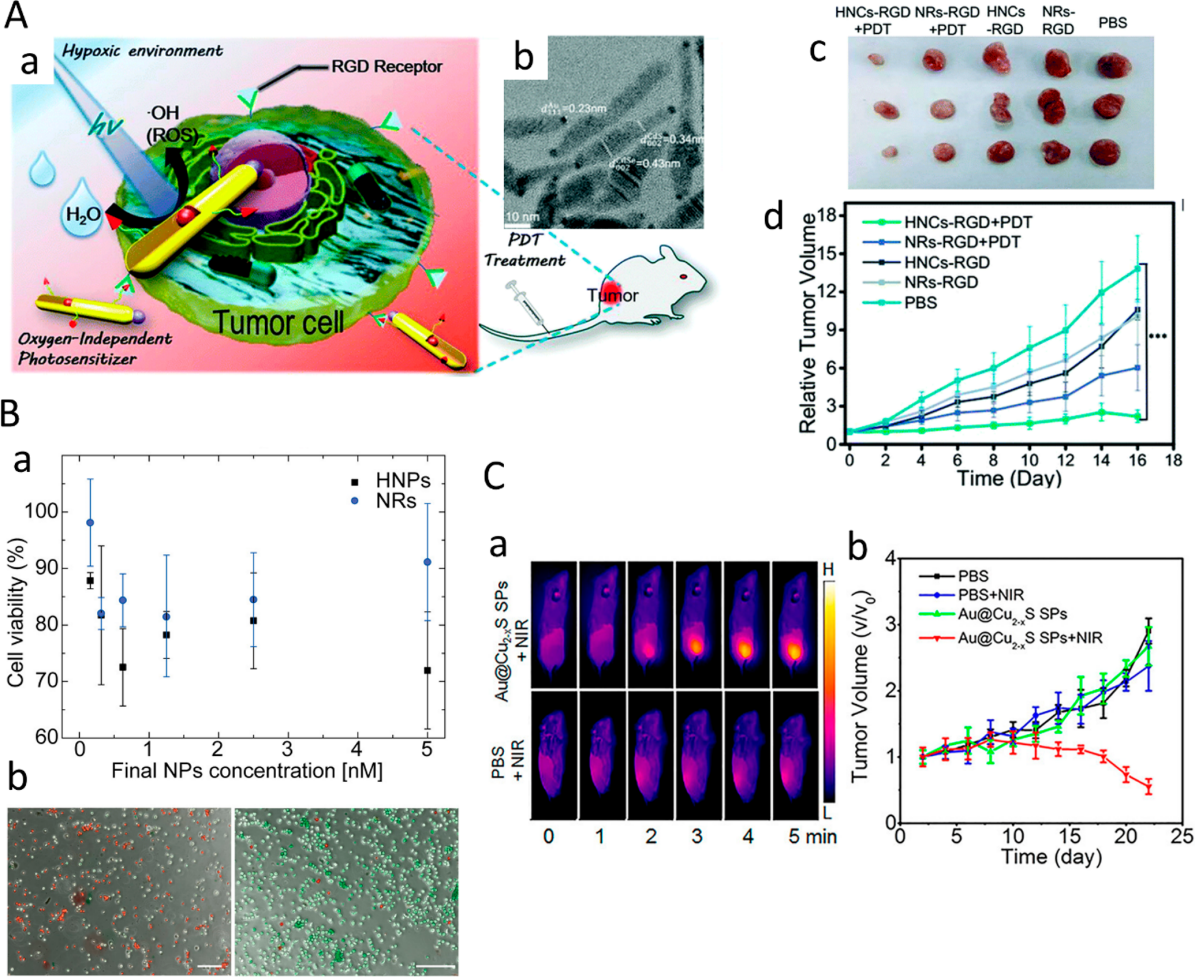
HNPs for photodynamic therapy. (A) (a) Schematic diagram of visible-light-driven water splitting to generate ROS for photodynamic-therapy (PDT) treatment. (b) HR-TEM image of the HNPs (scale bar 10 nm); *d*-spacing values 0.23, 0.34, and 0.43 nm are assigned to Au (111), CdS (002), and CdSe (002), respectively. (c) Representative tumor digital photos after various treatments for 16 days. (d) Relative tumor volumes from the different treatment groups: compared with CdSe/CdS nanorods (NRs), the CdSe/CdS–Au (HNCs) exhibited enhanced light-triggered PDT tumor growth suppression after being modified with an Arg-Gly-Asp (RGD) peptide sequence. (B) (a) MTT viability assay with cultured K-562 cells shows that their incubation for 24 h with different concentrations of NRs and HNPs did not significantly affect their viability. (b) Live/dead assay 24 h after illumination on cells incubated with 2.5 and 0.5 nM HNPs (left and right, respectively). Higher HNPs concentration shows significant cells death (red stained cells), an outcome that indicates a potential use for photodynamic therapy; scale bar 100 μm. HNPs for photothermal therapy. (C) (a) Infrared thermal images of 4T1 tumor after mice were intravenously injected with the HNPs solution (200 μL, 2.0 mg mL^–1^) and then laser irradiated (808 nm, 1.5 W cm^–2^) for 5 min. PBS injection is employed as control. (d) Tumor growth inhibition profiles of four groups of mice with time post-treatments. Relative tumor volume was normalized to their initial sizes for buffer solutions (PBS) and Au–Cu_2–*x*_S SPs solutions with and without NIR irradiation. (A) Adapted with permission from ref (296). Copyright 2017 Royal Society of Chemistry. (B) Adapted with permission from ref (287). Copyright 2016 American Chemical Society. (C) Adapted with permission from ref (292). Copyright 2017 American Chemical Society.

As mentioned above, excitation of metal–semiconductor HNPs by light in aerobic and aqueous environments results in ROS formation. They can be used for biomedical treatments of photodynamic therapy. This was demonstrated in vivo by the decreased volume of tumors and in vitro by the decreased cancer cell viability (Figure 42A). Another way of treatment by local heating via the plasmonic response of a metal or a semiconductor in photothermal therapy, which requires a high absorption coefficient and the coupling of the semiconductor to plasmonic metal, can enhance the absorption cross section (Figure 42C).

Utilization of HNPs for photothermal therapy was shown on Au–Cu_2–*x*_S, demonstrating enhancement of the extinction coefficient by a factor of 2 compared to the mixture of Cu_2–*x*_S and Au NPs in the relevant spectral regime. This factor was reported to yield an increase in the temperature achieved by Au–Cu_2–*x*_S by up to 70 °C at a high concentration of ∼0.1 mg/mL.^291,292^ This was also shown to correlate with the plasmon intensity of the gold NPs.^293^ The photothermal effect of Au–Cu_2–*x*_S showed antitumor activity in vitro, where only 10–20% of cervical cancer HeLa cells remained alive after NIR illumination for several minutes.^291,294^ Murine breast cancer 4T1 cells were killed by laser irradiation for 24 h but not normal 3T3 cells.^292^ Similar results were also reported for AuPt–CuS.^295^ In an in vivo experiment, a decrease in tumor volume was demonstrated in the presence of Au–Cu_2–*x*_S and AuPt–CuS with NIR irradiation.^292,293,295^ The survival of the mice of control groups was around 40 days post-treatment, whereas the lifetime of the Au–Cu_2–*x*_S-treated mice can be substantially prolonged, to 60 days post-treatment.^292^

In photodynamic therapy, HNPs can contribute to the antitumor activity via the photocatalytic ROS formation. The ROS can be formed either by oxidation of water or by the reduction of O_2_ as detailed in previous sections. It was described for the above Au/CuS and AuPt/CuS HNPs that resonant energy transfer from the excited plasmon in the metal core to excitons in the Cu_2–*x*_S shell supplies holes for water oxidation and electrons for O_2_ reduction.^293,295^ Another way that Au metal on a semiconductor can contribute to the antitumor activity is via its catalytic activity. The catalytic efficiency of Au is known to appear in a small size regime of <3 nm of the Au. The cell viability of leukemia cells K-562 was studied in the presence of visible 1.6 nm Au-tipped CdSe/CdS nanorods and demonstrated enhancement in cell death compared to bare CdSe/CdS nanorods.^287^ Cell viability test of similar CdSe/CdS–Au structures on 4T1 cells demonstrated almost complete cell death of 95% at a concentration of 0.1 mg mL^–1^ under hypoxia, while without the Au tip; less than 70% of the cells died.^296^ In this small size of gold there is no plasmonic response, and this activity was attributed to the generation of ROS. Specifically, CdSe/CdS–Au demonstrated better photocatalytic activity toward the formation of H_2_O_2_ and OH radicals and the consumption of oxygen compared to bare nanorods.^287,296^ This functionality of HNPs as an antibacterial agent was also demonstrated by Au–Bi_2_S_3_ core–shell structures under the illumination of NIR light, which showed superior antibacterial activities against both *E. coli* and *S. aureus* due to the synergistic photothermal and photodynamic killing.^297^

#### 4.4.2 Bioimaging

**Figure 43:**
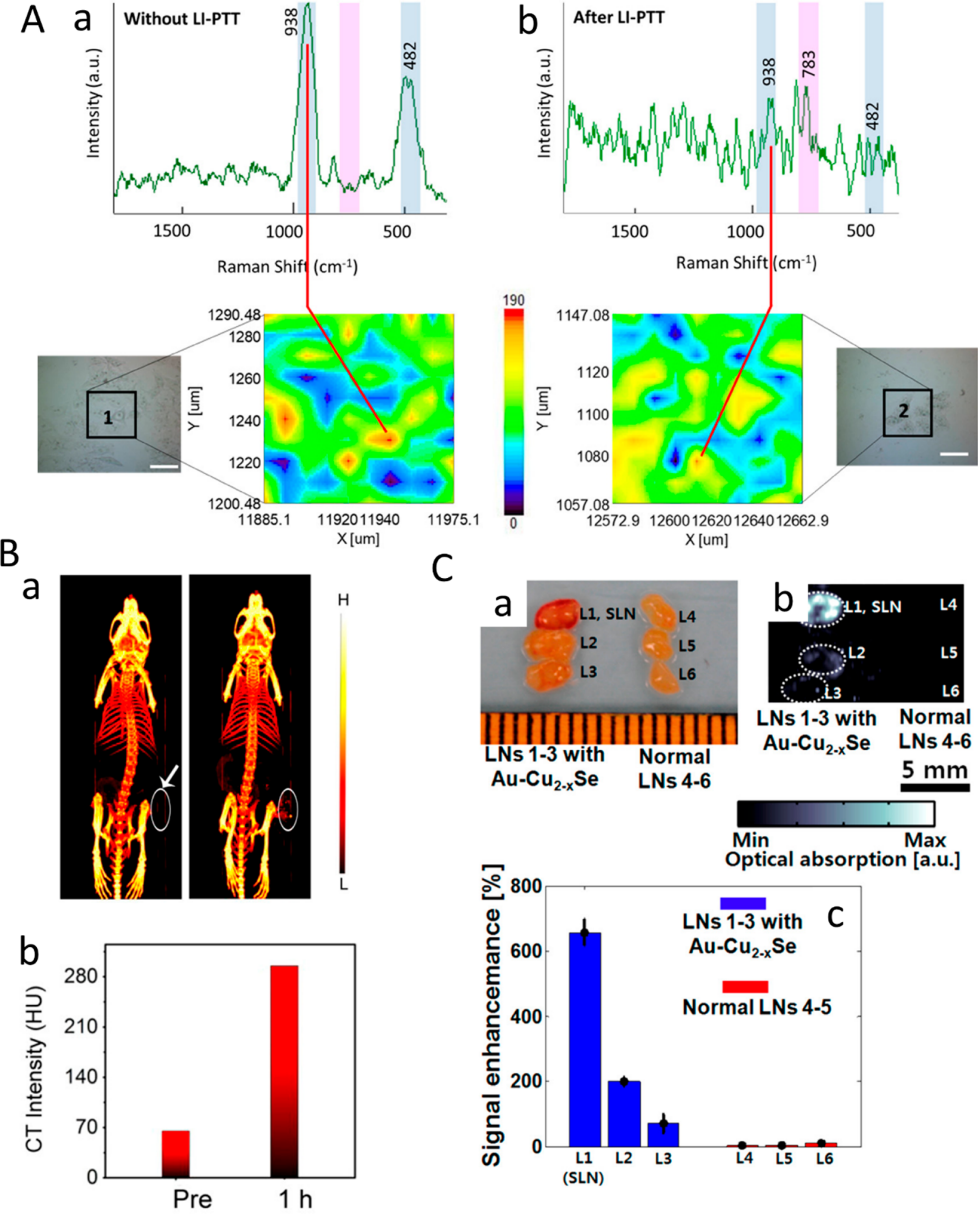
Surface-enhanced Raman scattering imaging by HNPs. (A) Bright-field image (scale bar 50 μm), corresponding SERS mapping image, and spectra of HeLa cells incubated with 330 μg/mL Au–Cu_2–*x*_S–Au NPs: (a) without LI-PtT and (b) after LI-PTT. Raman maps were collected from an area of 90 × 90 μm^2^ for the 938 cm^–1^ Raman shift using 785 nm laser irradiation. Points 1 and 2 represent the location from where the inset SERS spectra were recorded. Computed tomography imaging by HNPs. (B) (a) Three-dimensional in vivo CT mouse images of a tumor (indicated by the white circles) before (left) and after (right) intratumoral injection of the SPs. (b) Intensities of CT signals corresponding to a. Photoacoustic imaging by HNPs. (C) (a) Photograph and (b) PA MAP image of the excised lymph nodes (LNs) after injection of Au–Cu_2–*x*_Se NCs (L1–3) and the normal LNs (L4–6) ex vivo. L1 is the sentinel lymph node (SLN). (C) Quantification of the PA amplitudes measured from the excised LNs. (A) Adapted with permission from ref (294). Copyright 2021 American Chemical Society. (B) Adapted with permission from ref (292). Copyright 2017 American Chemical Society. (C) Adapted with permission from ref (299). Copyright 2013 American Chemical Society.

The enhanced absorption of the semiconductor induced by the metal plasmon was investigated as a contrasting agent in bioimaging applications. Surface-enhanced Raman spectroscopy (SERS) imaging was demonstrated on hybrid Au–Cu_2–*x*_S. The characteristic Raman peaks of the rhodamine B dye molecule were enhanced by a factor on the order of 10^4^ for trilayer core–shell NPs (Figure 43A).^294^ In these cases, the benefit from the semiconductor comes from the plasmonic rather than the band-gap properties. Nonplasmonic CdSe nanowires decorated with gold were also used to enhance the Raman signal of cresyl violet dye, but in this system, CdSe has only a structural role.^298^ X-ray-computed tomography (CT) is another imaging technique studied in the context of HNP activity. High image contrast at the tumor sites could be observed after Au–Cu_2–*x*_S or AuPt–CuS HNP injection, significantly larger than that of iopromide or iohexol (Figure 43B).^291,292,295^ The hybrids of Au–Cu_2–*x*_S and Au–Cu_2–*x*_Se were investigated also as contrast agents for photoacoustic (PA) imaging (Figure 43C).^291,292,299^ In another work, such HNPs were further combined with magnetic Fe_3_O_4_ to form Fe_3_O_4_–Au–Cu_2–*x*_S trimers for dual-heating agents based on photothermal and magnetic hyperthermia actuation. An intercalation protocol with radioactive ^64^Cu ions on the Cu_2–*x*_S domain reached high radiochemical yield and specific activity, making these HNP trimers suitable as carriers for ^64^Cu in internal radiotherapy and traceable by positron emission tomography.^300^

In the above examples of imaging techniques, there is no band-gap emission of the semiconductor in the HNPs and their contribution is the plasmon absorption or structural characteristic. This is due to the quenching of the emission when the metal is in direct contact with the semiconductor. Growth of an insulating layer between the semiconductor and the metal can prevent this quenching. In this way, the presence of the metal plasmon can contribute to the enhancement of the semiconductor emission. Such structures were demonstrated as labels for immunoassay by the structures of Au and CdSe/CdS/ZnS NPs embedded in SiO_2_. Bimodal labels with colorimetric and fluorescent readout were fabricated via a layered sequential assembly strategy. This resulted in amplified signals from the assemblies of individual single nanoparticles and allowed colorimetric and fluorescent detection of cystatin C (Cys C) after surface conjugation with antibodies.^301^

## 5 Outlook and Perspective

This review surveyed the remarkable accumulated research on semiconductor–metal HNPs, from their synthesis to properties to emergent photocatalytic applications. Yet, there is a prominent future for such HNPs that hinges on continued further research and innovation to meet the requirements for efficient photocatalytic reactions. Among the challenges, it is important to consider the introduction of novel synthetic approaches toward the formation of more complex HNP architectures. There is also a need to expand the HNP family to green and environmentally friendly semiconductor–metal systems. For example, the earth-abundant copper– and zinc–chalcogenide metal combination has not yet reached the full potential in their development owing to limited spectral coverage of the visible spectrum, the electronic properties, the charge extraction efficiency, or the chemical stability in aqueous solutions. III–V semiconductors as components in HNPs also hold promise due to their compliance with regulatory restrictions and their potential also as photocatalytic systems but have yet to be fully studied in these contexts. Recent progress in the synthesis of anisotropic shapes of InP nanocrystals and their surface control opens the way for development of new HNPs based on these materials.^302,303^ Moreover, addressing the stability of HNPs in diverse demanding photocatalytic scenarios is also important. In this context, expanding the functionality while controlling the HNP surface coating while also utilizing inorganic ligands may be further addressed.

Open questions concerning the fundamental nature of the nanoscale semiconductor–metal interplay in HNPs remain and call for further experimental and theoretical investigations. With the development of plasmonic photocatalysis, HNPs could offer a unique platform for dual combined excitonic and plasmonic photocatalysis in a single nanosystems. Yet, following this avenue motivates the study of electronic structure and charge carrier dynamics and relaxation routes across the excitonic–plasmonic interface. Harnessing the plasmonic excitation route requires one to extract the charge carriers on an ultrafast time scale from either side of the HNPs, which can only be achieved via further multidisciplinary efforts combining synthesis, theory, and advanced experiments. An additional interesting avenue is to integrate HNPs as building blocks in new photocatalytic applications such as photoelectrochemical solar to fuel conversion schemes, which also constitutes a highly promising direction of further research.
